# Supplementary material for: 1,5-Diarylidene-4-Piperidones as Promising Antifungal Candidates Against Cryptococcus neoformans
Source: Antibiotics (Basel). 2025 Sep 1;14(9):883. doi: 10.3390/antibiotics14090883 (PMC12466757; doi:10.3390/antibiotics14090883)
Supplement: Supplementary file 1 [file antibiotics-14-00883-s001.zip › antibiotics-3801119-supplementary.pdf]

1,5-Diarylidene-4-piperidones As Promising Antifungal Candidates Against *Cryptococcus neoformans*

Elise Courvoisier-Dezord[1], Hugo Ragusa[2], Axelle Grandé[1], Louise Denudt[1], Yolande Charmasson[1], Frédéric Dumur[1], Didier Siri[2], Marc Maresca[1*], Malek Nechab[2*]

[1] Aix Marseille Univ, CNRS, Centrale Marseille, iSm2, 13013 Marseille, France
[2] Aix Marseille Univ, CNRS, ICR UMR 7273, F-13397 Marseille, France
* Correspondence: m.maresca@univ-amu.fr (M.M.); malek.nechab@univ-amu.fr (M.N.)

1- Spectral data

Spectral data for compounds with MIC<125 µM

3,5-bis((E)-2,4-dimethoxybenzylidene)-1-ethylpiperidin-4-one (2a)[51]

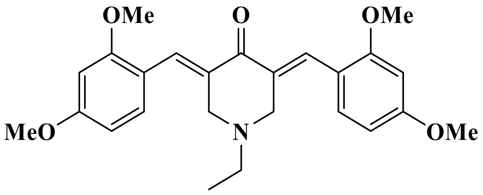

Chemical Formula: $C_{25}H_{29}NO_5$
Molecular Weight: 423.5090

^{1}H NMR (400 MHz, CDCl$_3$) δ 7.78 (d, *J* = 1.8 Hz, 1H), 7.42 – 7.33 (m, 2H), 6.99 – 6.91 (m, 2H), 3.87 – 3.80 (m, 5H), 2.63 (q, *J* = 7.2 Hz, 1H), 1.08 (t, *J* = 7.2 Hz, 1H).

^{13}C NMR (101 MHz, CDCl$_3$) δ 187.40, 160.39, 136.32, 132.44, 128.25, 114.25, 55.49, 54.59, 51.42, 12.60.

Yield : 68%. Yellowish solid : m.p. decomposition (>230°C)

3,5-bis((E)-2,4-dibutoxybenzylidene)-1-ethylpiperidin-4-one (2h)

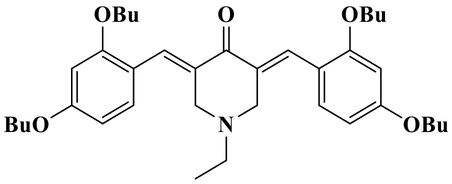

Chemical Formula: $C_{37}H_{53}NO_5$
Molecular Weight: 591.8330

[51] Schmitt, F.; Subramaniam, D.; Anant, S.; Padhye, S.; Begemann, G.; Schobert, R.; Biersack, B. Halogenated Bis(Methoxybenzylidene)-4-piperidone Curcuminoids with Improved Anticancer Activity. *ChemMedChem* **2018**, *13*, 1115–1123, doi:10.1002/cmdc.201800135.

^{1}H NMR (400 MHz, CDCl$_3$) δ 8.01 (d, *J* = 1.6 Hz, 1H), 7.05 (d, *J* = 8.3 Hz, 1H), 6.44 – 6.35 (m, 2H), 3.90 (m, 4H), 3.67 (d, *J* = 1.7 Hz, 2H), 2.49 (q, *J* = 7.2 Hz, 1H), 1.79 – 1.64 (m, 5H), 1.45 – 1.33 (m, 4H), 0.95 – 0.84 (m, 8H).

^{13}C NMR (101 MHz, CDCl$_3$) δ 187.24, 161.47, 159.76, 132.37, 131.39, 131.23, 117.77, 104.90, 99.74, 68.36, 67.90, 54.92, 51.04, 31.40, 31.27, 31.23, 19.39, 19.35, 13.94, 12.61.

HRMS (ESI MS) m/z: theor: 592.3997 found: 592.3996 ([M-H]$^+$ detected).

Yield : 77%. Yellowish solid : m.p. = 86-87°C.

1-ethyl-3,5-bis((E)-2,3,4-tributoxybenzylidene)piperidin-4-one (2m)

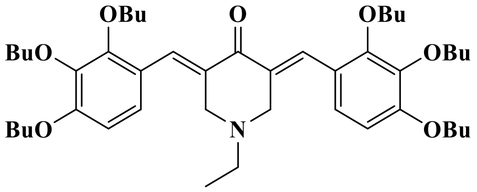

Chemical Formula: $C_{45}H_{69}NO_7$
Molecular Weight: 736.0470

^{1}H NMR (400 MHz, CDCl3) δ 8.02 (d, *J* = 1.9 Hz, 2H), 6.92 (d, *J* = 8.7 Hz, 2H), 6.66 (d, *J* = 8.7 Hz, 2H), 4.07 – 3.92 (m, 12H), 3.74 (d, *J* = 1.7 Hz, 4H), 2.55 (q, *J* = 7.2 Hz, 2H), 1.92 – 1.67 (m, 12H), 1.51 (m, 17H), 0.98 (ddt, *J* = 17.3, 9.0, 7.3 Hz, 20H).

^{13}C NMR (126 MHz, CDCl$_3$) δ 187.03, 154.65, 153.50, 142.12, 132.53, 132.23, 125.07, 122.91, 107.72, 74.42, 73.48, 68.54, 54.79, 50.90, 32.48, 32.44, 31.48, 19.43, 19.36, 19.33, 14.06, 13.97, 12.67.

HRMS (ESI MS) m/z: theor: 736.5147 found: 736.5146 ([M-H]$^+$ detected).

Yield : 59%. Yellowish solid : m.p. = 90-91°C.

3,5-*Bis*((1*H*-pyrrol-2-yl)methylene)-1-benzylpiperidin-4-one (3j)[52]

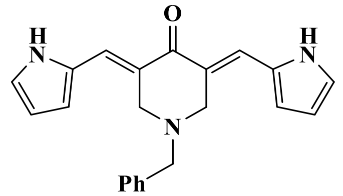

Chemical Formula: $C_{22}H_{21}N_3O$
Molecular Weight: 343.4300

[52] Andreani, A.; Cavalli, A.; 10.1016/j.ejmech.2015.07.014, M.; Guardigli, M.; Leoni, A.; Locatelli, A.; Morigi, R.; Rambaldi, M.; Recanatini, M.; Roda, A. Synthesis and Screening for Antiacetylcholinesterase Activity of (1-Benzyl-4-Oxopiperidin-3-Ylidene)Methylindoles and -Pyrroles Related to Donepezil. *J. Med. Chem.* **2001**, *44*, 4011–4014, doi:10.1021/jm0109356.

^{1}H NMR (400 MHz, CDCl$_3$) δ 8.76 (s, 2H), 7.67 (s, 2H), 7.49 – 7.27 (m, 6H), 6.98 (s, 2H), 6.46 – 6.23 (m, 4H), 3.92 – 3.55 (m, 6H).

^{13}C NMR (101 MHz, DMSO_d$_6$) 54.2, 111.1, 113.3, 122.6, 124.2, 127.1, 127.1, 128.1, 128.2, 128.8, 129.0, 138.0, 184.8

HRMS (ESI MS) m/z: theor: 344.1757 found: 344.1758 ([M-H]$^+$ detected).

Yield : 85%. Brown solid : m.p. decomposition (>190°C)

1-benzyl-3,5-bis((E)-2,4-dibutoxybenzylidene)piperidin-4-one (3h)

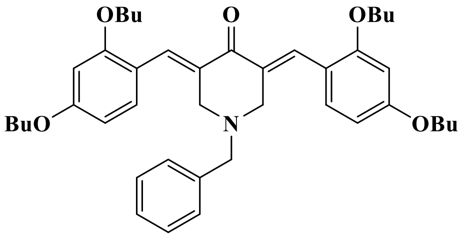

Chemical Formula: $C_{42}H_{55}NO_5$
Molecular Weight: 653.9040

^{1}H NMR (400 MHz, CDCl$_3$) δ 8.10 (s, 2H), 7.24 – 7.15 (m, 5H), 7.04 (d, *J* = 8.5 Hz, 2H), 6.46 – 6.39 (m, 4H), 3.97 (td, *J* = 6.6, 3.1 Hz, 10H), 3.80 (d, *J* = 1.7 Hz, 4H), 3.66 (s, 2H), 1.87 – 1.72 (m, 10H), 1.51 (tt, *J* = 9.5, 6.5 Hz, 10H), 0.99 (t, *J* = 7.4 Hz, 14H).

^{13}C NMR (101 MHz, CDCl$_3$) δ 187.62, 161.43, 159.70, 137.92, 132.33, 131.33, 131.30, 129.19, 128.35, 128.26, 127.17, 117.73, 104.94, 99.73, 68.38, 67.90, 60.96, 60.45, 54.86, 31.39, 31.29, 19.41, 19.34, 13.95, 13.92.

HRMS (ESI MS) m/z: theor: 654.4153 found: 654.4452 ([M-H]$^+$ detected).

Yield : 73%. Yellowish amorphous solid.

1-benzyl-3,5-bis((E)-3,4-dibutoxybenzylidene)piperidin-4-one (3iso-h)

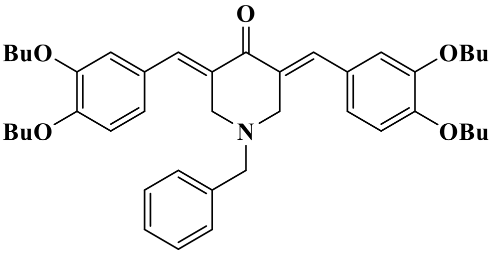

Chemical Formula: $C_{42}H_{55}NO_5$
Molecular Weight: 653.9040

^{1}H NMR (400 MHz, CDCl$_3$) 0.99 (t, 6H, J = 7.4 Hz), 1.00 (t, 6H, J = 7.4 Hz), 1.43-1.56 (m, 8H), 1.77-1.87 (m, 8H), 3.73 (s, 2H), 3.86 (s, 4H), 3.97 (t, 4H, J = 6.5 Hz), 4.03 (t, 4H, J = 6.5 Hz), 6.85-6.95 (m, 6H), 7.18-7.28 (m, 5H), 7.73 (s, 2H).

^{13}C NMR (101 MHz, CDCl$_3$) 13.8, 13.9, 19.2, 19.3, 31.2, 31.3, 54.5, 61.7, 68.8, 69.0, 113.0, 116.1, 124.5, 127.4, 128.2, 128.3, 129.0, 131.4, 136.4, 137.4, 148.7, 150.2, 187.4.

HRMS (ESI MS) m/z: theor: 654.4153 found: 654.4458 ([M-H]$^+$ detected).

Yield : 65%. Yellowish amorphous solid.

Spectral data for Compounds with MIC>125 µM

1-ethyl-3,5-bis((E)-4-methoxybenzylidene)piperidin-4-one (1a)[53]

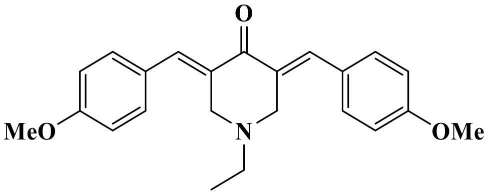

Chemical Formula: $C_{23}H_{25}NO_3$
Molecular Weight: 363.4570

^{1}H NMR (400 MHz, CDCl3) δ 7.78 (d, *J* = 1.8 Hz, 1H), 7.42 – 7.33 (m, 2H), 6.99 – 6.91 (m, 2H), 3.87 – 3.80 (m, 5H), 2.63 (q, *J* = 7.2 Hz, 1H), 1.08 (t, *J* = 7.2 Hz, 1H).

^{13}C NMR (101 MHz, CDCl$_3$) δ 187.40, 160.39, 136.32, 132.44, 131.55, 128.25, 114.25, 55.49, 54.59, 51.42, 12.60.

Yield : 85%. Yellowish solid : m.p. = 158-159°C.

1-ethyl-3,5-bis((E)-4-methylbenzylidene)piperidin-4-one (1b)[54]

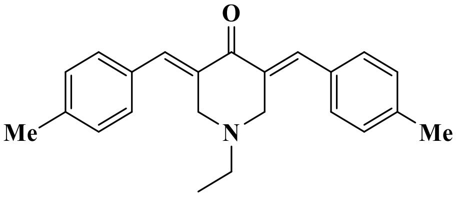

Chemical Formula: $C_{23}H_{25}NO$
Molecular Weight: 331.4590

^{1}H NMR (400 MHz, CDCl$_3$) δ 7.80 (s, 2H), 7.35 – 7.28 (m, 4H), 7.23 (d, *J* = 8.0 Hz, 4H), 3.83 (d, *J* = 1.8 Hz, 4H), 2.61 (q, *J* = 7.2 Hz, 2H), 2.39 (s, 5H), 1.06 (t, *J* = 7.1 Hz, 3H).

^{13}C NMR (101 MHz, CDCl$_3$) δ 187.59, 139.37, 136.59, 132.75, 132.73, 130.63, 129.44, 54.61, 51.41, 21.55, 12.58.

---

[53] Bayomi, S.M.; El-Kashef, H.A.; El-Ashmawy, M.B.; Nasr, M.N.A.; El-Sherbeny, M.A.; Abdel-Aziz, N.I.; El-Sayed, M.A.-A.; Suddek, G.M.; El-Messery, S.M.; Ghaly, M.A. Synthesis and Biological Evaluation of New Curcumin Analogues as Antioxidant and Antitumor Agents: Molecular Modeling Study. *Eur. J. Med. Chem.* **2015**, *101*, 584–594, doi:10.1016/j.ejmech.2015.07.014.

[54] Girgis, A.S.; Panda, S.S.; Farag, I.S.A.; 10.25135/acg.oc.69.19.09.1399, A.M.; Moustafa, A.M.; Ismail, N.S.M.; Pillai, G.G.; Panda, C.S.; Hall, C.D.; Katritzky, A.R. Synthesis, and QSAR Analysis of Anti-Oncological Active Spiro-Alkaloids. *Org. Biomol. Chem.* **2015**, *13*, 1741–1753, doi:10.1039/C4OB02149E.

Yield : 79%. Yellowish solid : m.p. = 124-126°C.

4,4'-((1E,1'E)-(1-ethyl-4-oxopiperidine-3,5-diylidene)bis(methaneylylidene)) dibenzonitrile (1c)[55]

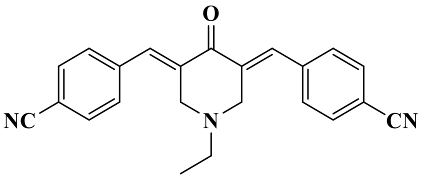

Chemical Formula: $C_{23}H_{19}N_3O$
Molecular Weight: 353.4250

^{1}H NMR (400 MHz, CDCl$_3$) δ 7.77 (s, 1H), 7.75 – 7.69 (m, 2H), 7.48 (d, *J* = 8.1 Hz, 2H), 4.78 – 4.73 (m, 4H), 3.78 (d, *J* = 2.0 Hz, 2H), 2.62 (q, *J* = 7.1 Hz, 1H), 1.05 (t, *J* = 7.1 Hz, 2H).

^{13}C NMR (101 MHz, CDCl$_3$) δ 186.68, 139.84, 139.64, 135.64, 134.62, 132.44, 132.40, 130.70, 130.67, 118.51, 112.56, 54.25, 51.45, 12.41, 12.38.

Yield : 84%. Yellowish solid : m.p. décomposition (>210°C).

1-ethyl-3,5-bis((E)-4-nitrobenzylidene)piperidin-4-one (1d)

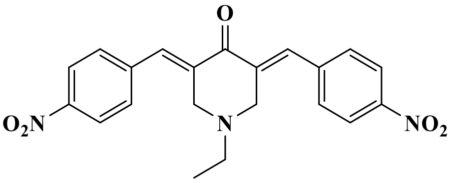

Chemical Formula: $C_{21}H_{19}N_3O_5$
Molecular Weight: 393.3990

^{1}H NMR (400 MHz, CDCl$_3$) δ 8.34 – 8.27 (m, 4H), 7.84 (s, 2H), 7.60 – 7.53 (m, 4H), 3.83 (d, *J* = 1.9 Hz, 4H), 2.65 (q, *J* = 7.2 Hz, 2H), 1.07 (t, *J* = 7.1 Hz, 3H).

^{13}C NMR (101 MHz, CDCl$_3$) δ 186.51, 147.66, 141.42, 135.94, 134.17, 130.83, 123.87, 54.18, 51.38, 12.27.

Yield : 67%. Yellowish solid : m.p. décomposition (>200°C).

HRMS (ESI MS) m/z: theor: 394.1397 found: 394.1396 ([M-H]$^+$ detected).

3,5-bis((E)-4-chlorobenzylidene)-1-ethylpiperidin-4-one (1e)[53]

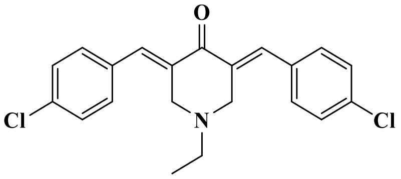

Chemical Formula: $C_{21}H_{19}Cl_2NO$
Molecular Weight: 372.2890

[55] Parlar, S. Synthesis and Cholinesterase Inhibitory Activity Studies of Some Piperidinone Derivatives. Org. Commun. **2019**, *12*, 202–209, doi:10.25135/acg.oc.69.19.09.1399.

^{1}H NMR (400 MHz, CDCl$_3$) δ 7.75 (d, *J* = 1.9 Hz, 1H), 7.44 – 7.34 (m, 2H), 7.37 – 7.29 (m, 2H), 3.78 (d, *J* = 1.8 Hz, 2H), 2.61 (q, *J* = 7.2 Hz, 1H), 1.06 (t, *J* = 7.2 Hz, 1H).

^{13}C NMR (101 MHz, CDCl$_3$) δ 187.13, 135.41, 135.23, 131.70, 129.02, 54.39, 51.43, 12.49.

Yield : 82%. Yellowish solid : m.p. décomposition (>160°C).

1-ethyl-3,5-bis((E)-4-(trifluoromethyl)benzylidene)piperidin-4-one (1f)[56]

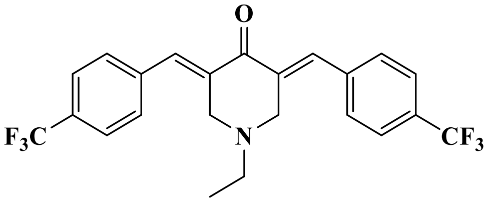

Chemical Formula: $C_{23}H_{19}F_6NO$
Molecular Weight: 439.4014

^{1}H NMR (400 MHz, CDCl$_3$) δ 7.81 (s, 2H), 7.69 (d, *J* = 8.1 Hz, 4H), 7.53 – 7.46 (m, 4H), 3.80 (d, *J* = 1.9 Hz, 4H), 2.62 (q, *J* = 7.2 Hz, 2H), 1.06 (t, *J* = 7.1 Hz, 3H).

^{13}C NMR (101 MHz, CDCl$_3$) δ 187.06, 138.75, 135.16, 135.04, 130.48, 127.07, 127.03, 125.74, 125.70, 125.66, 125.63, 54.33, 51.45, 12.43.

Yield : 88%. Yellowish solid : m.p. décomposition (>140°C).

3,5-bis((E)-2,4-dimethoxybenzylidene)-1-ethylpiperidin-4-one (2a)[51]

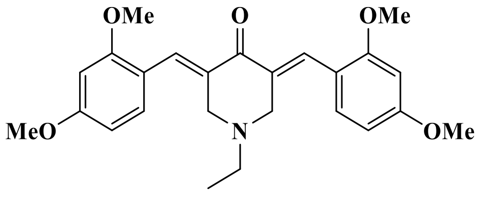

Chemical Formula: $C_{25}H_{29}NO_5$
Molecular Weight: 423.5090

^{1}H NMR (400 MHz, CDCl$_3$) δ 8.04 (d, *J* = 1.7 Hz, 1H), 7.15 (d, *J* = 8.4 Hz, 1H), 6.55 – 6.43 (m, 2H), 3.84 (d, *J* = 4.2 Hz, 6H), 3.75 (d, *J* = 1.7 Hz, 2H), 2.55 (q, *J* = 7.1 Hz, 1H), 1.01 (t, *J* = 7.1 Hz, 2H).

Yield : 79%. Yellowish solid : m.p. décomposition (>250°C).

---

[56] Hanachi, R.; Said, R.B.; Rahali, S.; Tangour, B.; Abdelwahab, S.I.; Farasani, A.; M. E. Taha, M.; Bidwai, A.; Koko, W.S.; Khan, T.A.; et al. P-Trifluoromethyl- and p-Pentafluorothio-Substituted Curcuminoids of the 2,6-Di[(E)-Benzylidene)]Cycloalkanone Type: Syntheses and Activities against Leishmania Major and Toxoplasma Gondii Parasites. *Bioorganic Chem.* **2021**, *114*, 105099, doi:10.1016/j.bioorg.2021.105099.

1-Ethyl-3,5-bis((E)-2,4-bis(allyloxy)benzylidene)piperidin-4-one (2g)[24]

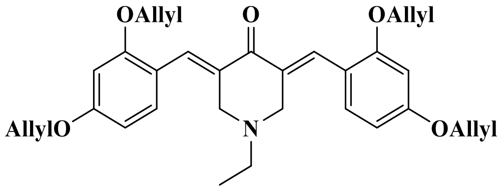

Chemical Formula: $C_{33}H_{37}NO_5$
Molecular Weight: 527.6610

^{1}H NMR (400 MHz, $CDCl_3$) δ : 1.02 (t, 3H, J = 7.1 Hz), 2.56 (q, 2H, J = 7.1 Hz), 3.75 (brs, 4H), 4.54-4.56 (m, 8H), 5.25-5.35 (m, 4H), 5.37-5.47 (m, 4H), 5.99-6.10 (m, 4H), 6.48-6.53 (m, 4H), 7.14 (d, 1H, J = 8.2 Hz), 8.10 (s, 2H),

^{13}C NMR (101 MHz, $CDCl_3$) δ : 12.4, 54.6, 69.0, 69.2,100.3, 105.5, 117.7, 118.0, 118.1, 131.3, 132.8, 132.9, 159.0, 160.7.

Yield : 84%. Orange amorphous solid.

(3E,5E)-3,5-bis(benzo[d][1,3]dioxol-5-ylmethylene)-1-ethylpiperidin-4-one (2i)[57]

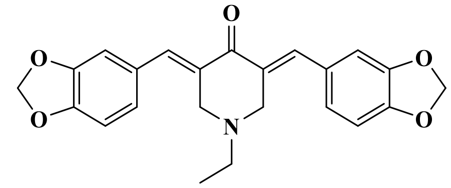

Chemical Formula: $C_{23}H_{21}NO_5$
Molecular Weight: 391.4230

^{1}H NMR (400 MHz, $CDCl_3$) δ 7.71 (d, *J* = 1.7 Hz, 1H), 6.94 (ddd, *J* = 8.1, 1.8, 0.7 Hz, 1H), 6.94 – 6.83 (m, 2H), 6.01 (s, 2H), 3.78 (d, *J* = 1.8 Hz, 2H), 2.62 (q, *J* = 7.1 Hz, 1H), 1.09 (t, *J* = 7.2 Hz, 2H).

Yield : 88%. Yellowish solid : m.p.= 159-160°C.

[24] Xu, Y.; Noirbent, G.; Brunel, D.; Ding, Z.; Gigmes, D.; Graff, B.; Xiao, P.; Dumur, F.; Lalevée, J. Allyloxy Ketones as Efficient Photoinitiators with High Migration Stability in Free Radical Polymerization and 3D Printing. *Dyes Pigment.* **2021**, *185*, 108900. https://doi.org/10.1016/j.dyepig.2020.108900.

[57] Insuasty, B.; Becerra, D.; Quiroga, J.; Abonia, R.; Nogueras, M.; Cobo, J. Microwave-Assisted Synthesis of Pyrimido[4,5-b][1,6]Naphthyridin-4(3H)-Ones with Potential Antitumor Activity. *Eur. J. Med. Chem.* **2013**, *60*, 1–9, doi:10.1016/j.ejmech.2012.11.037.

1-ethyl-3,5-bis((E)-2-fluorobenzylidene)piperidin-4-one (2j)[58]

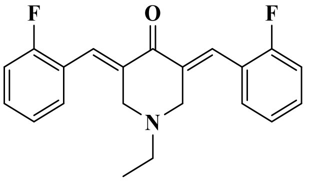

Chemical Formula: $C_{21}H_{19}F_2NO$
Molecular Weight: 339.3858

^{1}H NMR (400 MHz, $CDCl_3$) δ 7.92 – 7.87 (m, 2H), 7.41 – 7.26 (m, 6H), 7.23 – 7.08 (m, 4H), 3.74 – 3.67 (m, 4H), 2.56 (q, *J* = 7.2 Hz, 2H), 1.01 (t, *J* = 7.1 Hz, 3H).

Yield : 78%. Yellowish solid : m.p. décomposition (>230°C).

(3E,5E)-3,5-bis((1H-pyrrol-2-yl)methylene)-1-ethylpiperidin-4-one (2k)[52]

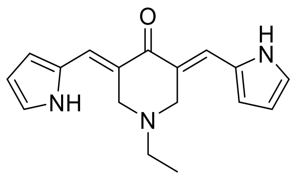

Chemical Formula: $C_{17}H_{19}N_3O$
Molecular Weight: 281.3590

^{1}H NMR (400 MHz, DMSO) δ 11.47 (s, 2H), 7.53 (s, 2H), 7.09 (td, *J* = 2.7, 1.2 Hz, 2H), 6.44 (p, *J* = 1.6 Hz, 2H), 6.28 (dt, *J* = 4.2, 2.3 Hz, 2H),3.65 (s, 4H), 2.64 (q, *J* = 7.1 Hz, 2H), 1.09 (t, *J* = 7.1 Hz, 3H).

^{13}C NMR (101 MHz, DMSO) δ 184.95, 128.23, 127.23, 124.23, 122.68, 113.57, 111.10, 53.85, 50.87, 12.36.

Yield : 88%. Yellowish solid : m.p. décomposition (>190°C).

(3E,5E)-1-ethyl-3,5-bis(quinolin-3-ylmethylene)piperidin-4-one (2l)

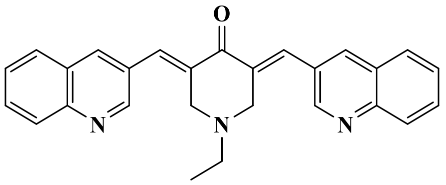

Chemical Formula: $C_{27}H_{23}N_3O$
Molecular Weight: 405.5010

^{1}H NMR (400 MHz, $CDCl_3$) δ 8.99 (d, *J* = 2.3 Hz, 2H), 8.20 – 8.10 (m, 4H), 7.99 (d, *J* = 1.8 Hz, 2H), 7.89 (dd, *J* = 8.1, 1.5 Hz, 2H), 7.78 (ddd, *J* = 8.4, 6.9, 1.5 Hz, 2H), 7.61 (ddd, *J* = 8.1, 6.9, 1.2 Hz, 2H), 3.97 (d, *J* = 1.8 Hz, 4H), 2.67 (q, *J* = 7.2 Hz, 2H), 1.07 (t, *J* = 7.1 Hz, 3H).

---

[58] Schmitt, F.; Gold, M.; Begemann, G.; Andronache, I.; Biersack, B.; Schobert, R. Fluoro and Pentafluorothio Analogs of the Antitumoral Curcuminoid EF24 with Superior Antiangiogenic and Vascular-Disruptive Effects. *Bioorg. Med. Chem.* **2017**, *25*, 4894–4903, doi:10.1016/j.bmc.2017.07.039.

^{13}C NMR (101 MHz, $CDCl_3$) δ 186.53, 151.60, 147.71, 137.25, 135.15, 133.29, 130.76, 129.49, 128.50, 128.44, 127.60, 127.50, 54.47, 51.41, 12.44.

Yield : 65%. Yellowish solid : m.p. décomposition (>160°C).

HRMS (ESI MS) m/z: theor: 406.1914 found: 406.1919 ($[M-H]^+$ detected).

2,4-bis((E)-2,4-dibutoxybenzylidene)-8-methyl-8-azabicyclo[3.2.1]octan-3-one (2n)

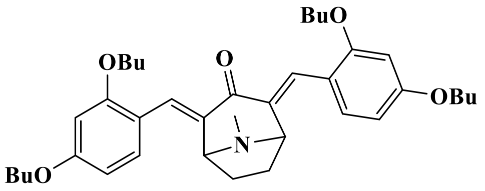

Chemical Formula: $C_{38}H_{53}NO_5$
Molecular Weight: 603.8440

^{1}H NMR (400 MHz, $CDCl_3$ δ 7.97 (s, 2H), 7.06 (d, *J* = 8.2 Hz, 2H), 6.42 (d, *J* = 8.3 Hz, 5H), 4.27 – 4.15 (m, 2H), 3.92 (qd, *J* = 6.6, 2.3 Hz, 10H), 2.47 (s, 2H), 2.23 (s, 3H), 1.92 (d, *J* = 7.1 Hz, 2H), 1.79 – 1.64 (m, 10H), 1.50 – 1.32 (m, 11H), 0.91 (q, *J* = 7.3 Hz, 14H), 0.79 – 0.75 (m, 1H).

^{13}C NMR (101 MHz, $CDCl_3$) δ 188.23, 161.45, 159.61, 136.57, 132.24, 131.44,117.76, 104.94, 99.99, 68.47, 67.98, 61.39, 35.56, 31.23, 30.75, 19.39, 13.96.

Yield : 68%. Yellowish solid m.p.= 97-98°C.

HRMS (ESI MS) m/z: theor: 604.3997 found: 604.3993 ($[M-H]^+$ detected).

1-benzyl-3,5-bis((E)-4-butoxybenzylidene)piperidin-4-one (2o)

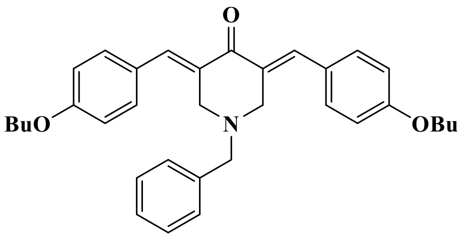

Chemical Formula: $C_{34}H_{39}NO_3$
Molecular Weight: 509.6900

^{1}H NMR (400 MHz, $CDCl_3$) δ 7.76 (s, 1H), 7.36 – 7.15 (m, 5H), 6.93 – 6.83 (m, 2H), 3.99 (t, *J* = 6.5 Hz, 2H), 3.85 (d, *J* = 1.7 Hz, 2H), 3.72 (s, 1H), 1.83 – 1.72 (m, 2H), 1.57 – 1.43 (m, 2H), 0.98 (t, *J* = 7.4 Hz, 3H).

^{13}C NMR (101 MHz, $CDCl_3$) δ 187.82, 159.97, 137.66, 136.31, 132.42, 131.50, 129.14, 128.47, 127.98, 127.44, 114.72, 67.93, 61.68, 54.71, 31.37, 19.36, 13.96.

Yield : 86%. Yellowish solid : m.p. =126-127°C.

1-benzyl-3,5-bis((E)-2,4-dimethoxybenzylidene)piperidin-4-one (3a)[59]

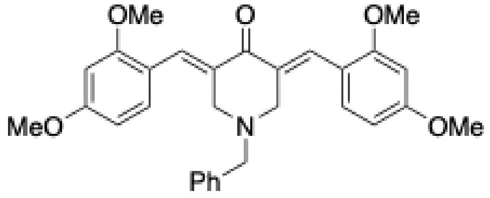

Chemical Formula: $C_{30}H_{31}NO_5$
Molecular Weight: 485,58

¹H NMR (400 MHz, CDCl₃) δ 8.05 (s, 1H), 7.24 – 7.11 (m, 2H), 7.04 (d, *J* = 8.3 Hz, 1H), 6.49 – 6.39 (m, 2H), 3.81 (dd, *J* = 15.2, 1.9 Hz, 9H), 3.64 (s, 1H).

¹³C NMR (101 MHz, CDCl₃) δ 187.94, 161.94, 160.07, 137.92, 132.23, 131.69, 131.32, 129.19, 128.29, 127.21, 117.69, 104.42, 98.42, 60.72, 55.63, 55.53, 54.59.

Yield : 72%. Yellowish solid : m.p. décomposition (>160°C).

1-cyclopropyl-3,5-bis((E)-2,4-dibutoxybenzylidene)piperidin-4-one (4h)

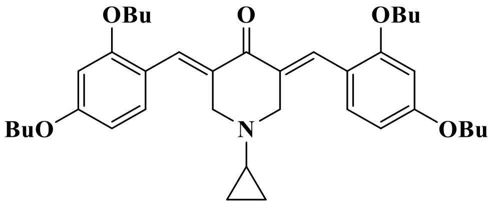

Chemical Formula: $C_{38}H_{53}NO_5$
Molecular Weight: 603.8440

1H NMR (400 MHz, CDCl₃) δ 8.07 (d, *J* = 1.7 Hz, 1H), 7.18 (d, *J* = 8.5 Hz, 1H), 6.54 – 6.44 (m, 2H), 3.99 (dt, *J* = 9.3, 6.5 Hz, 4H), 3.89 (d, *J* = 1.7 Hz, 2H), 1.79 (ddt, *J* = 14.4, 8.9, 6.5 Hz, 5H), 1.58 – 1.43 (m, 5H), 0.98 (dt, *J* = 8.7, 7.4 Hz, 7H).

¹³C NMR (101 MHz, CDCl₃) δ 187.34, 161.48, 159.86, 132.02, 131.48, 117.89, 104.96, 99.77, 68.41, 67.95, 55.78, 38.07, 31.45, 31.31, 19.43, 13.98, 6.86.

Yield : 70%. Yellowish solid : m.p. décomposition (>150°C).

HRMS (ESI MS) m/z: theor: 604.3997 found: 604.3996 ([M-H]⁺ detected).

---

[59] Zhou, D.; Ding, N.; Zhao, S.; Li, D.; Van Doren, J.; Qian, Y.; Wei, X.; Zheng, X. Synthesis and Evaluation of Curcumin-Related Compounds Containing Inden-2-One for Their Effects on Human Cancer Cells. *Biol. Pharm. Bull.* **2014**, *37*, 1977–1981, doi:10.1248/bpb.b14-00477.

tert-butyl 3,5-bis((E)-2,4-dibutoxybenzylidene)-4-oxopiperidine-1-carboxylate (5h)

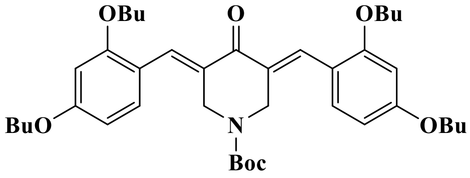

Chemical Formula: $C_{40}H_{57}NO_7$
Molecular Weight: 663.8960

^{1}H NMR (400 MHz, $CDCl_3$) δ 8.03 (s, 2H), 7.18 (s, 2H), 6.46 (s, 7H), 4.62 (s, 4H), 3.98 (t, *J* = 7.0 Hz, 18H), 1.86 – 1.77 (m, 14H), 1.76 (s, 4H), 1.55 – 1.45 (m, 20H), 1.29 (q, *J* = 4.5 Hz, 21H), 1.05 – 0.93 (m, 28H).

^{13}C NMR (101 MHz, $CDCl_3$) δ 187.96, 161.78, 159.82, 154.71, 131.52, 117.41, 105.10, 99.85, 80.19, 68.43, 45.34, 31.40, 28.29, 19.45, 14.00.

Yield : 48%. Yellowish solid : m.p. = 116-118°C.

HRMS (ESI MS) m/z: theor: 664.4208 found: 664.4203 ($[M-H]^+$ detected).

3,5-bis((E)-2,4-dibutoxybenzylidene)-1-(methylsulfonyl)piperidin-4-one (6h)

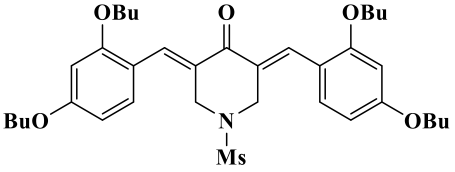

Chemical Formula: $C_{36}H_{51}NO_7S$
Molecular Weight: 641.8640

^{1}H NMR (400 MHz, $CDCl_3$) δ 8.22 – 8.17 (m, 1H), 7.11 (dd, *J* = 8.7, 5.0 Hz, 1H), 6.52 – 6.44 (m, 2H), 4.65 (d, *J* = 1.7 Hz, 1H), 4.00 (q, *J* = 5.9 Hz, 5H), 2.80 – 2.72 (m, 1H), 2.73 (s, 1H), 1.87 – 1.72 (m, 4H), 1.57 – 1.42 (m, 2H), 0.99 (qd, *J* = 7.7, 4.2 Hz, 6H).

^{13}C NMR (101 MHz, $CDCl_3$) δ 185.67, 162.35, 159.91, 135.01, 131.70, 128.16, 116.62, 105.38, 100.00, 68.43, 47.58, 39.14, 31.37, 19.43, 13.97.

Yield : 59%. Yellowish solid : m.p. décomposition (>130°C).

HRMS (ESI MS) m/z: theor: 642.3459 found: 642.3456 ($[M-H]^+$ detected).

benzyl 3,5-bis((E)-2,4-dibutoxybenzylidene)-4-oxopiperidine-1-carboxylate (7h)

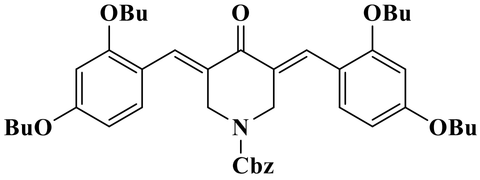

Chemical Formula: $C_{43}H_{55}NO_7$
Molecular Weight: 697.9130

1H NMR (400 MHz, CDCl3) δ 8.11 (s, 2H), 7.48 – 6.92 (m, 9H), 6.72 – 6.31 (m, 5H), 5.09 (d, *J* = 5.1 Hz, 2H), 4.76 (s, 4H), 4.04 (h, *J* = 7.0 Hz, 10H), 1.70 (ddt, *J* = 120.5, 15.4, 6.7 Hz, 21H), 1.19 – 0.84 (m, 15H).

^{13}C NMR (101 MHz, CDCl$_3$) δ 188.54, 187.43, 161.87, 159.82, 155.11, 136.63, 128.48, 127.80, 127.43, 117.20, 105.12, 99.90, 68.44, 67.98, 67.32, 31.41, 31.27, 19.42, 19.39, 13.97.

Yield : 64%. Yellowish solid : m.p. = 93-94°C.

HRMS (ESI MS) m/z: theor: 698.4051 found: 698.4055 ([M-H]$^+$ detected).

2,6-bis((E)-2,4-dibutoxybenzylidene)cyclohexan-1-one (8h)[60]

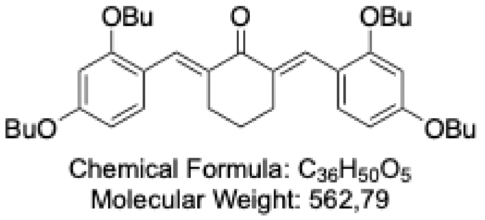

^{1}H NMR (400 MHz, CDCl$_3$) δ 8.01 (d, *J* = 1.9 Hz, 1H), 7.28 (dd, *J* = 9.5, 3.1 Hz, 1H), 6.53 – 6.40 (m, 2H), 3.99 (q, *J* = 7.2 Hz, 5H), 2.88 – 2.80 (m, 2H), 1.88 – 1.70 (m, 6H), 1.59 – 1.49 (m, 3H), 0.98 (td, *J* = 7.4, 2.9 Hz, 7H).

^{13}C NMR (101 MHz, CDCl$_3$) δ 190.36, 161.10, 159.63, 134.70, 132.15, 131.34, 118.40, 104.72, 99.72, 68.39, 67.91, 31.43, 31.31, 29.18, 23.75, 19.42, 19.37, 13.98, 13.96.

Yield : 84%. Yellowish solid : m.p. =79-80°C).

2,6-bis((E)-2,4-dibutoxybenzylidene)-4-methylcyclohexan-1-one (9h)

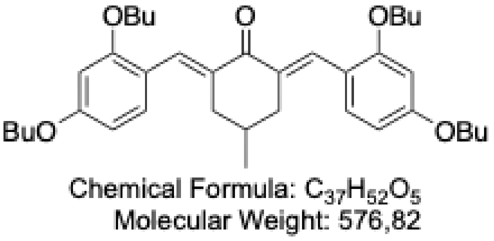

^{1}H NMR (400 MHz, CDCl$_3$) δ ^{1}H NMR (400 MHz, CDCl$_3$) δ 8.00 (d, *J* = 2.5 Hz, 1H), 7.31 – 7.25 (m, 1H), 6.54 – 6.43 (m, 2H), 3.98 (td, *J* = 6.6, 4.2 Hz, 5H), 2.97 (dd, *J* = 15.5, 3.6 Hz, 1H), 2.40 (ddd, *J* = 15.1, 11.5, 2.7 Hz, 1H), 1.91 – 1.72 (m, 5H), 1.57 – 1.41 (m, 5H), 1.07 – 0.92 (m, 9H).

^{13}C NMR (101 MHz, CDCl$_3$) δ 190.12, 161.11, 159.65, 133.91, 132.39, 131.40, 118.41, 104.74, 99.74, 68.40, 67.92, 37.38, 31.44, 31.32, 30.11, 22.00, 19.43, 19.39, 13.99, 13.98.

Yield : 78%. Yellowish solid : m.p. = 83-84°C.

---

[60] Sasidharan, A.K.; Mathew, J.; Achalkumar, A.S.; Mathews, M. Synthesis and Liquid Crystalline Properties of Low Molecular Weight Bis-Chalcone Compounds. *Curr. Org. Synth.* **2022**, *19*, 463–475, doi:10.2174/1570179418666211006144308.

HRMS (ESI MS) m/z: theor: 577.3888 found: 577.3840 ($[M-H]^+$ detected).

4-(tert-butyl)-2,6-bis((E)-2,4-dibutoxybenzylidene)cyclohexan-1-one (10h)

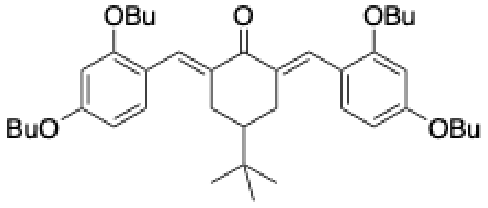

Chemical Formula: $C_{40}H_{58}O_5$
Molecular Weight: 618,90

^{1}H NMR (400 MHz, $CDCl_3$) δ 8.00 (d, *J* = 2.5 Hz, 1H), 7.34 – 7.25 (m, 1H), 6.55 – 6.40 (m, 3H), 4.08 – 3.89 (m, 5H), 3.07 (dd, *J* = 15.1, 3.2 Hz, 1H), 2.37 (ddd, *J* = 15.6, 12.7, 3.0 Hz, 1H), 1.89 – 1.72 (m, 5H), 1.59 – 1.47 (m, 5H), 1.05 – 0.87 (m, 13H).

^{13}C NMR (101 MHz, $CDCl_3$) δ 190.49, 161.03, 159.65, 134.47, 132.11, 131.15, 118.42, 104.83, 99.77, 68.40, 67.89, 44.77, 32.67, 31.45, 31.32, 30.08, 27.48, 19.42, 19.38, 13.99, 13.97, 13.91.

Yield : 68%. Yellowish amorphous solid.

HRMS (ESI MS) m/z: theor: 619.4357 found: 619.4350 ($[M-H]^+$ detected).

2- NMR spectra

1H NMR for 1a

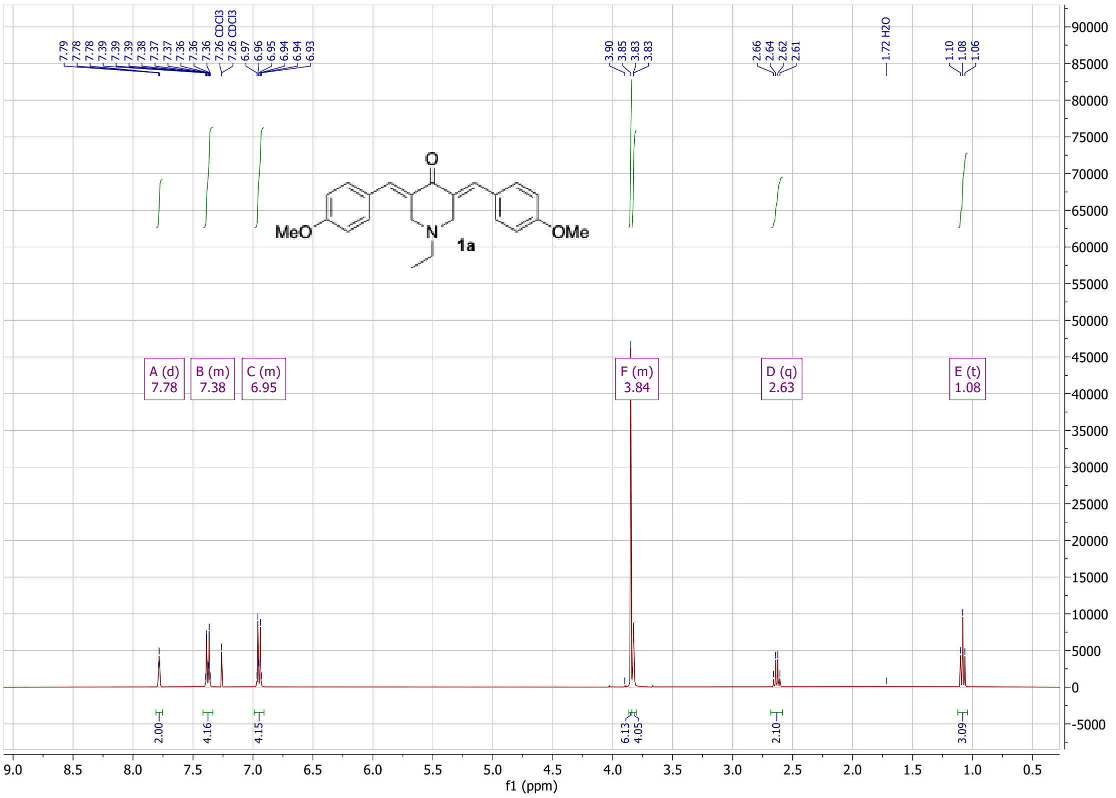

13C NMR for 1a

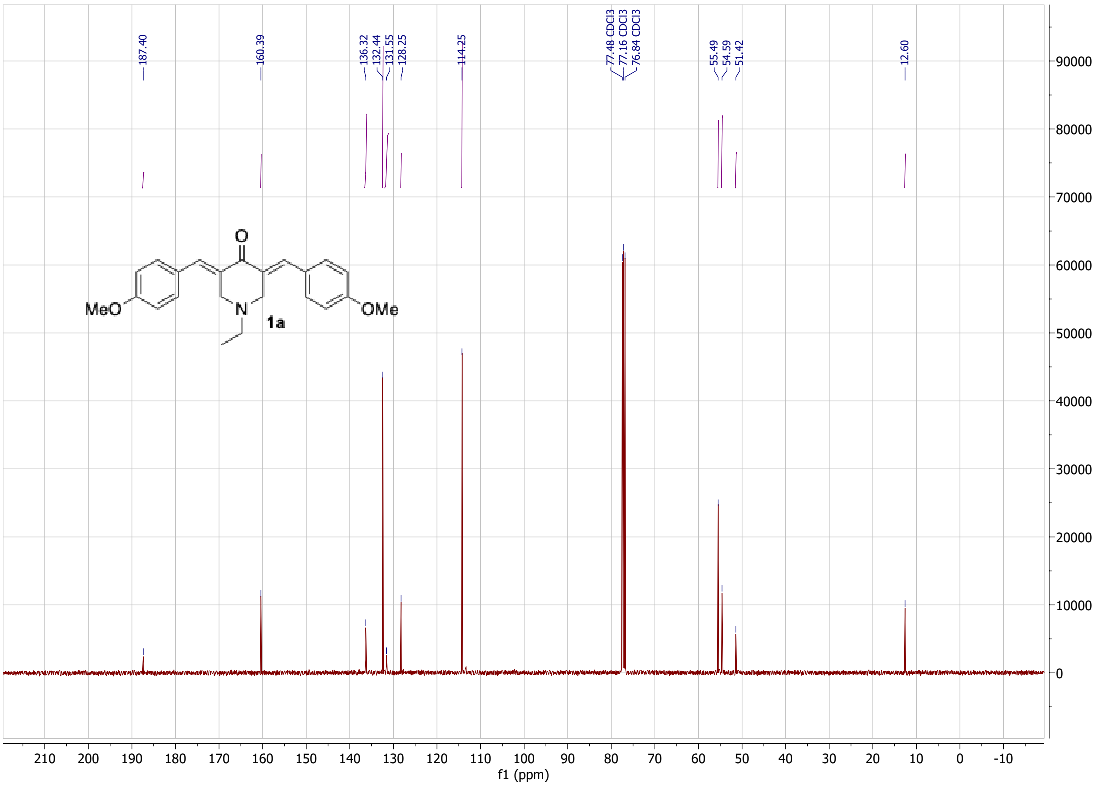

^{1}H NMR for 1b

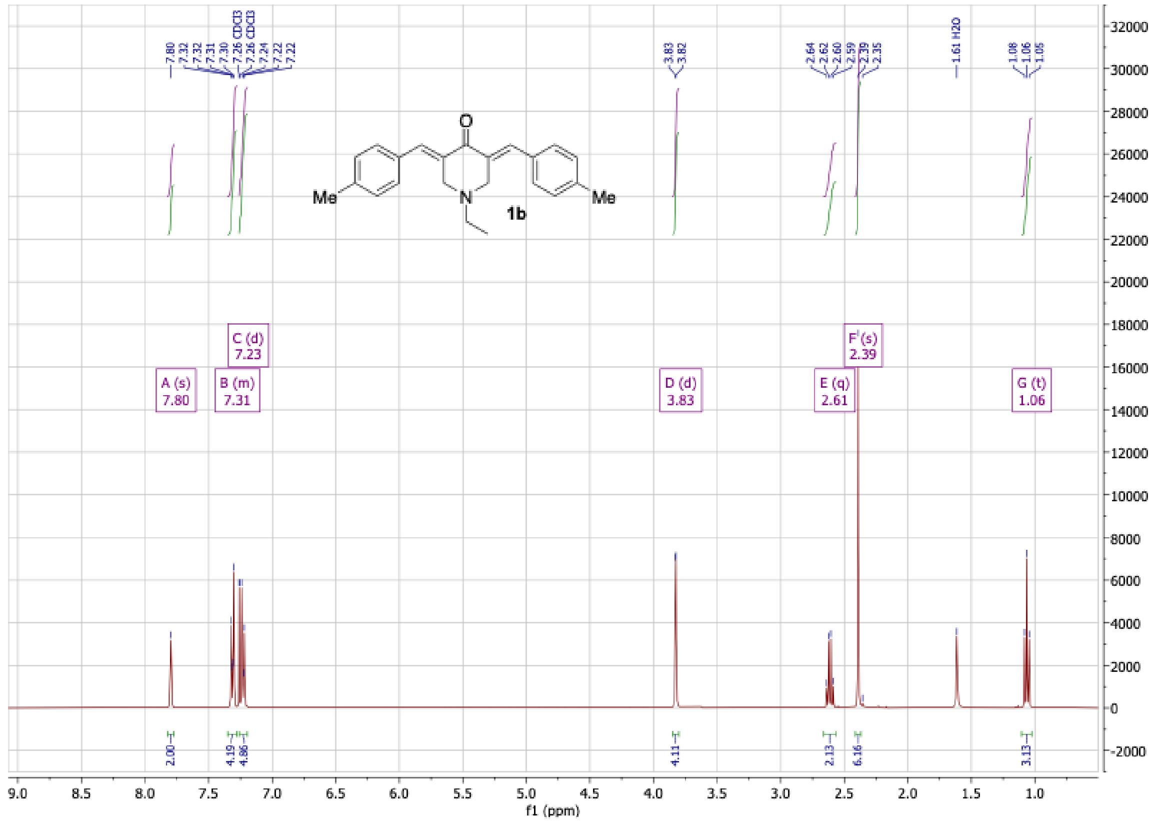

^{13}C NMR for 1b

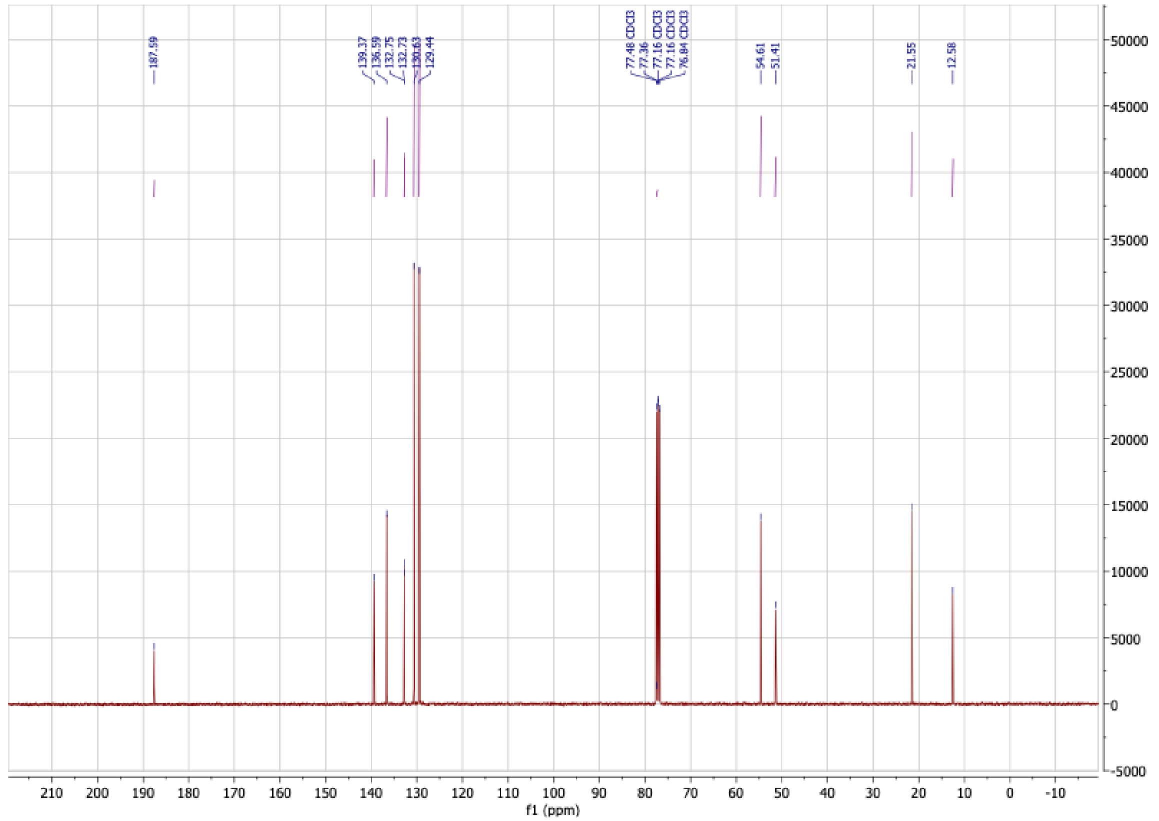

^{1}H NMR for 1c

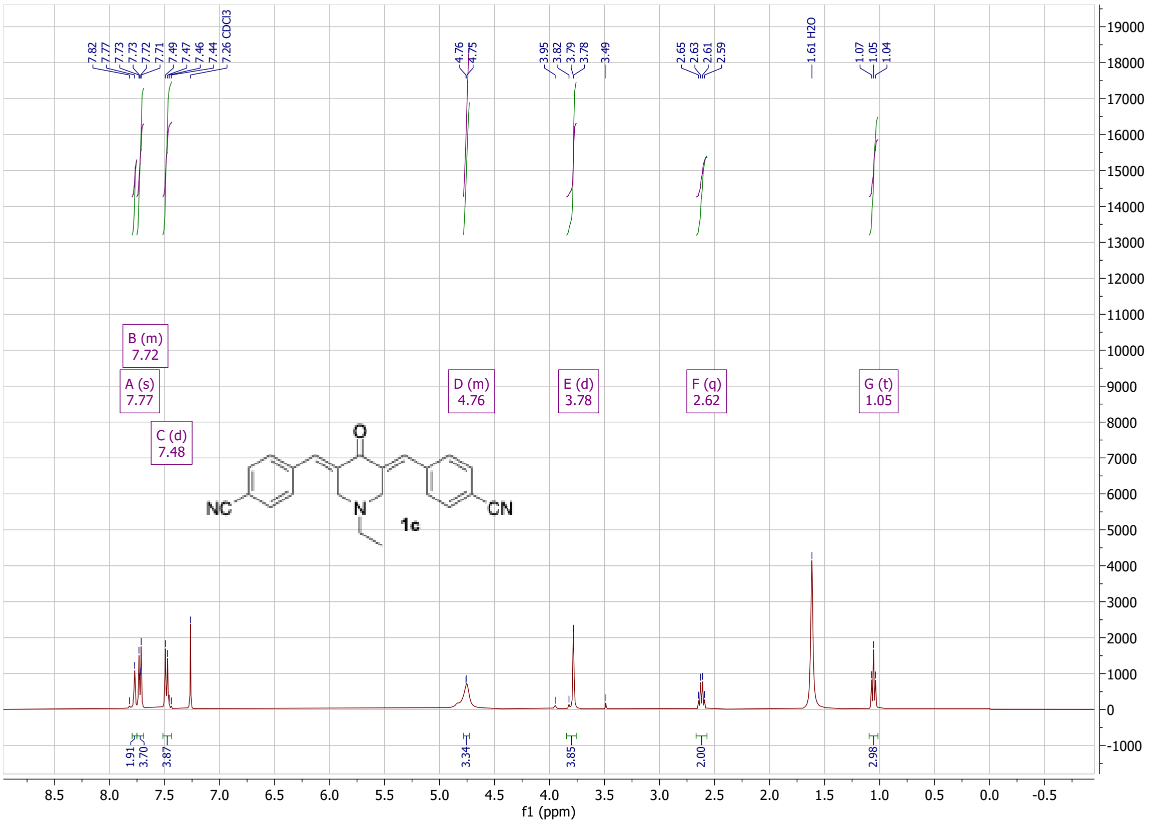

^{13}C NMR for 1c

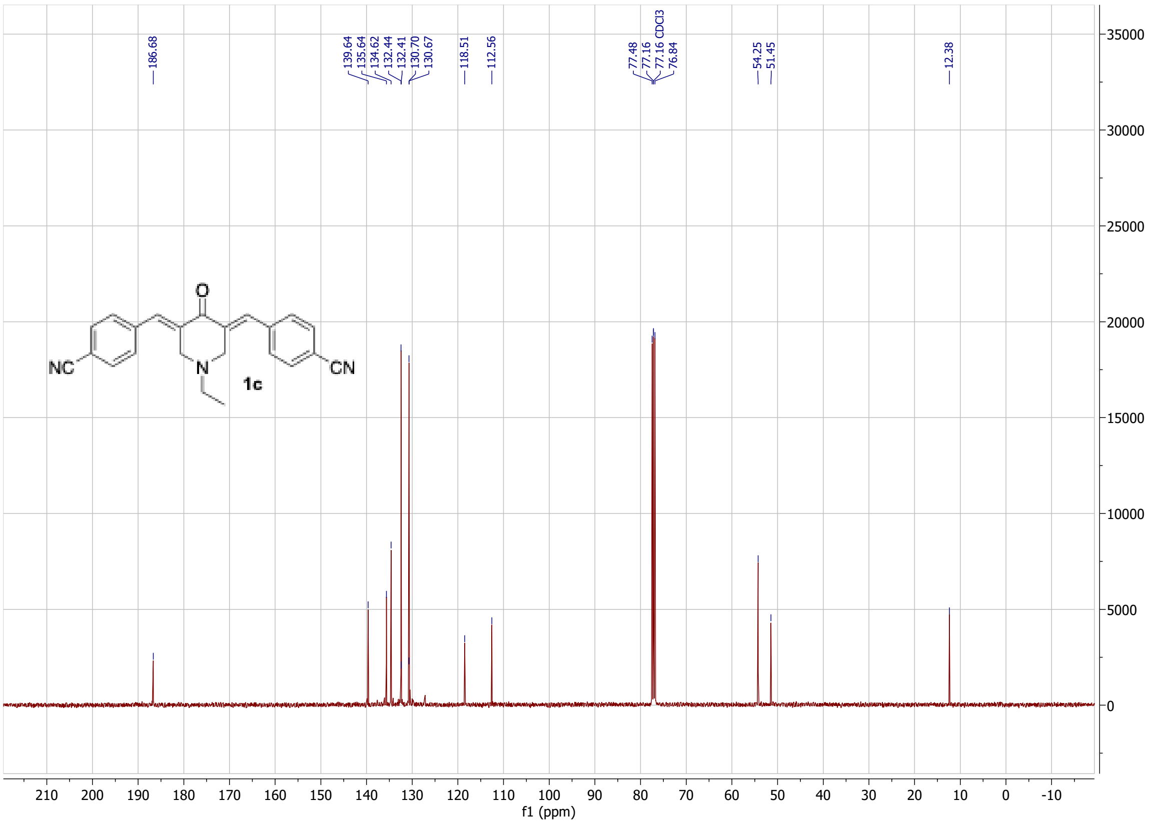

^{1}H NMR for 1d

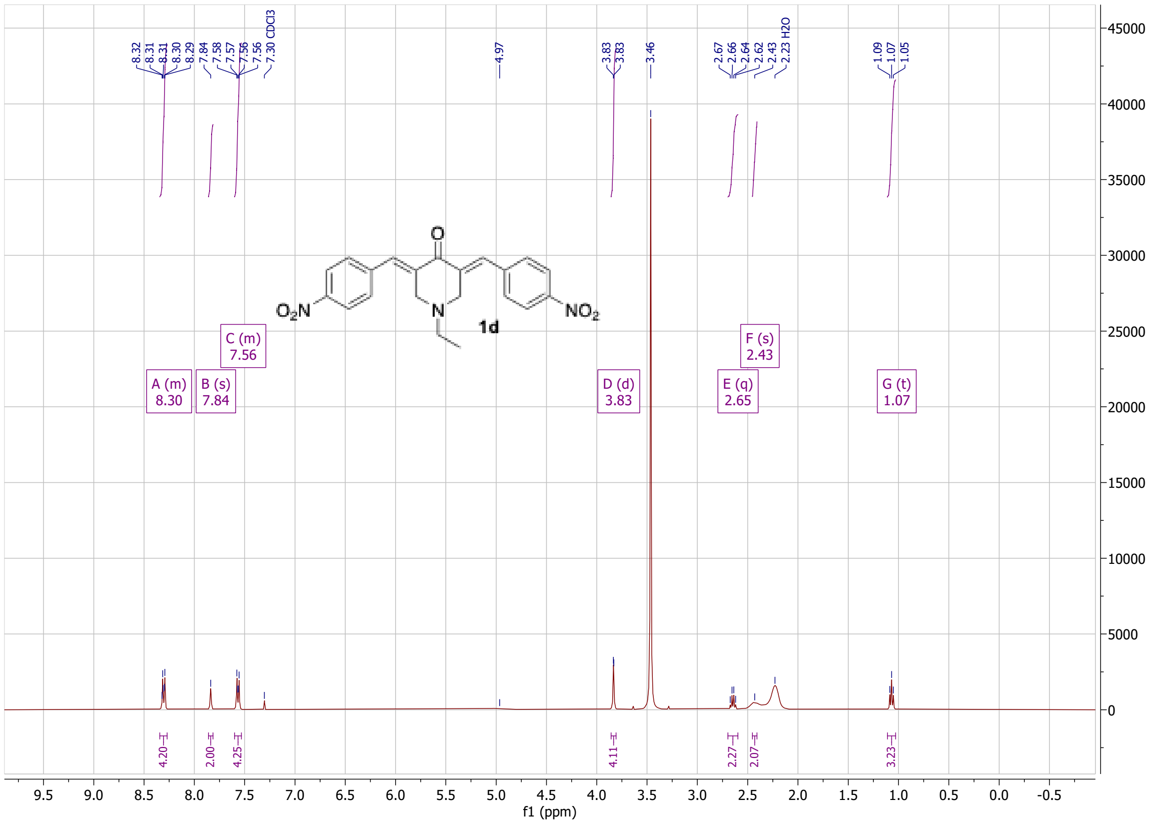

^{13}C NMR for 1d

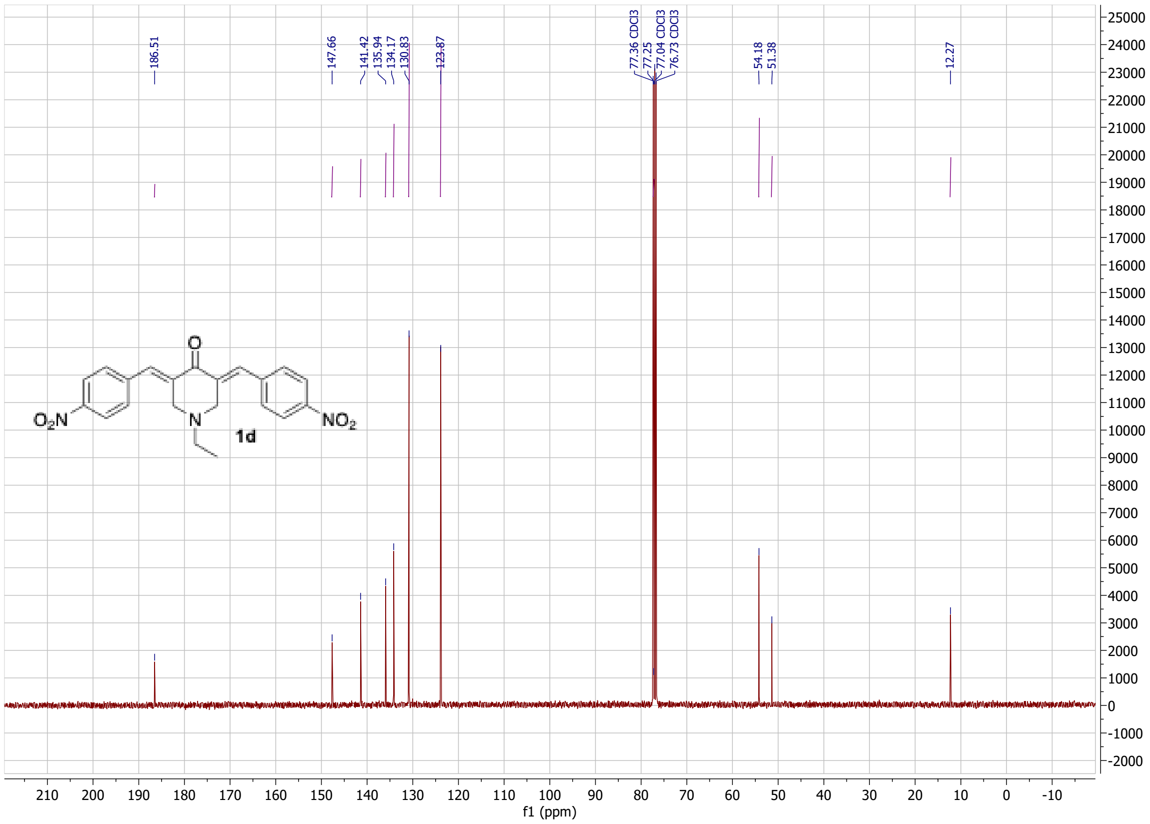

^{1}H NMR for 1e

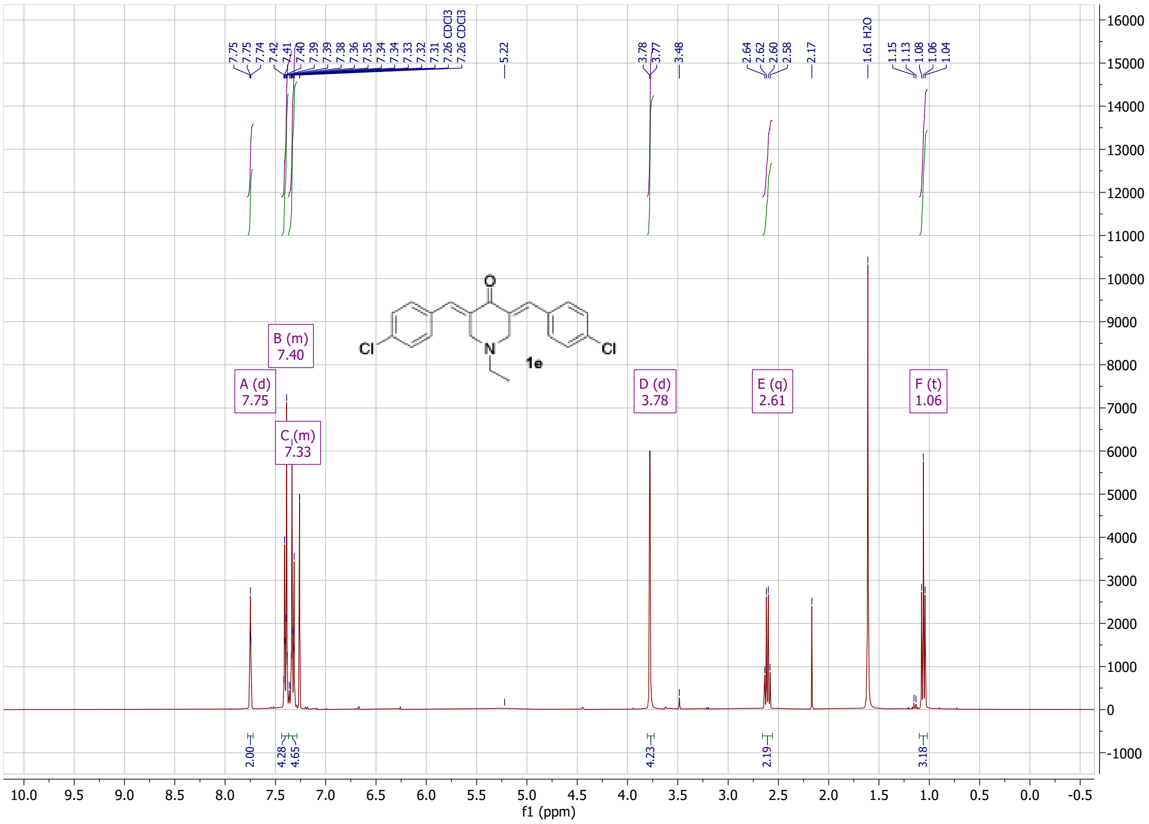

^{13}C NMR for 1e

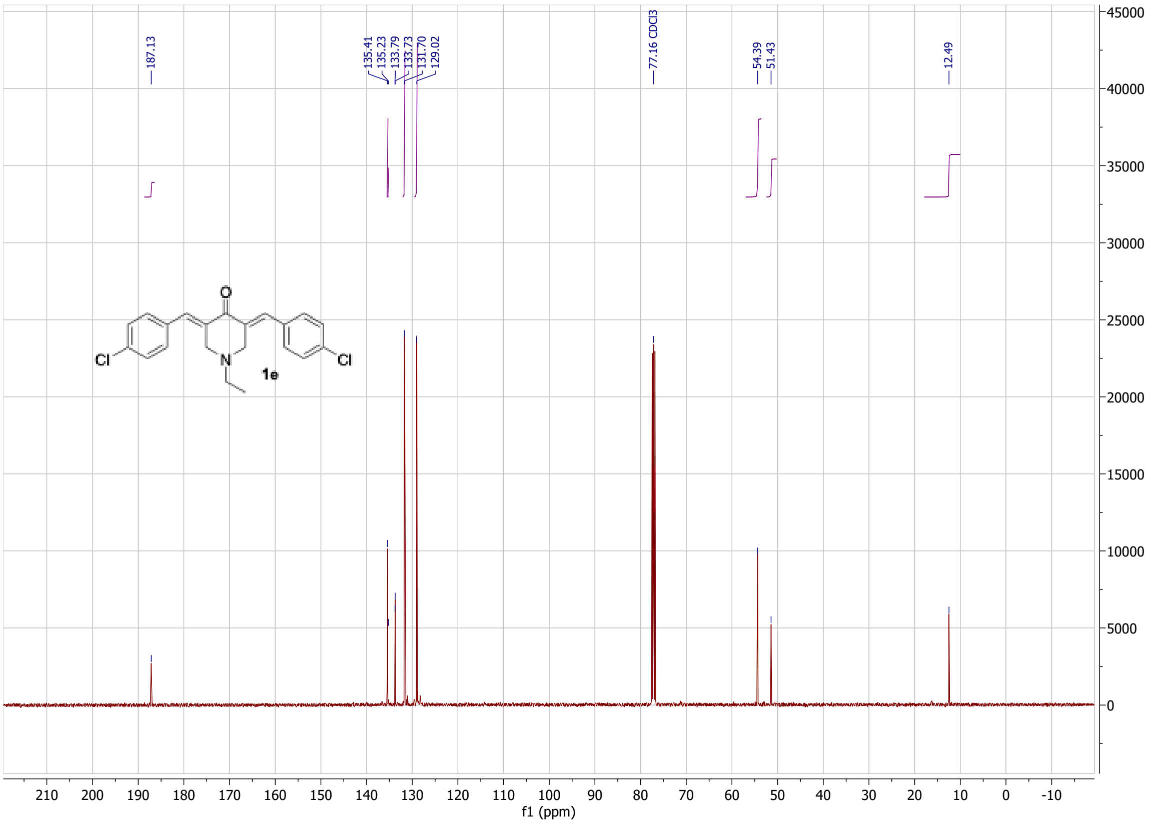

^{1}H NMR for 1f

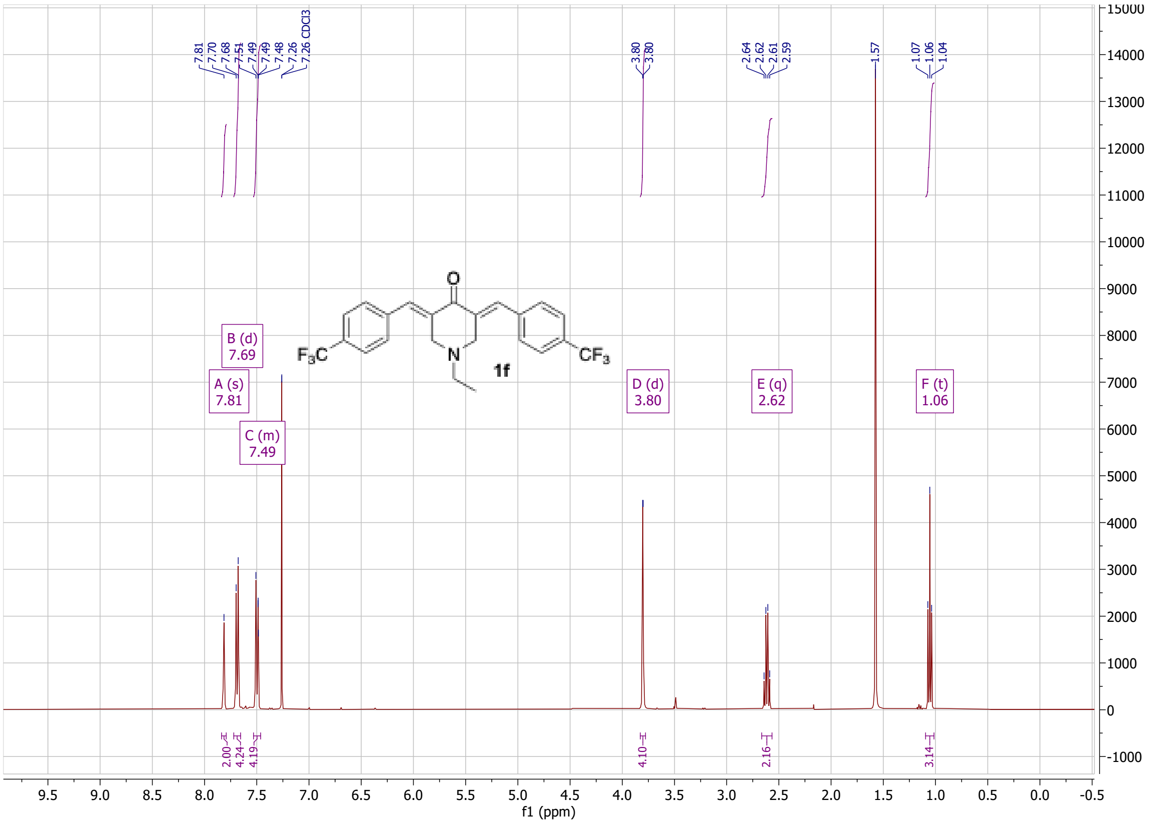

^{13}C NMR for 1f

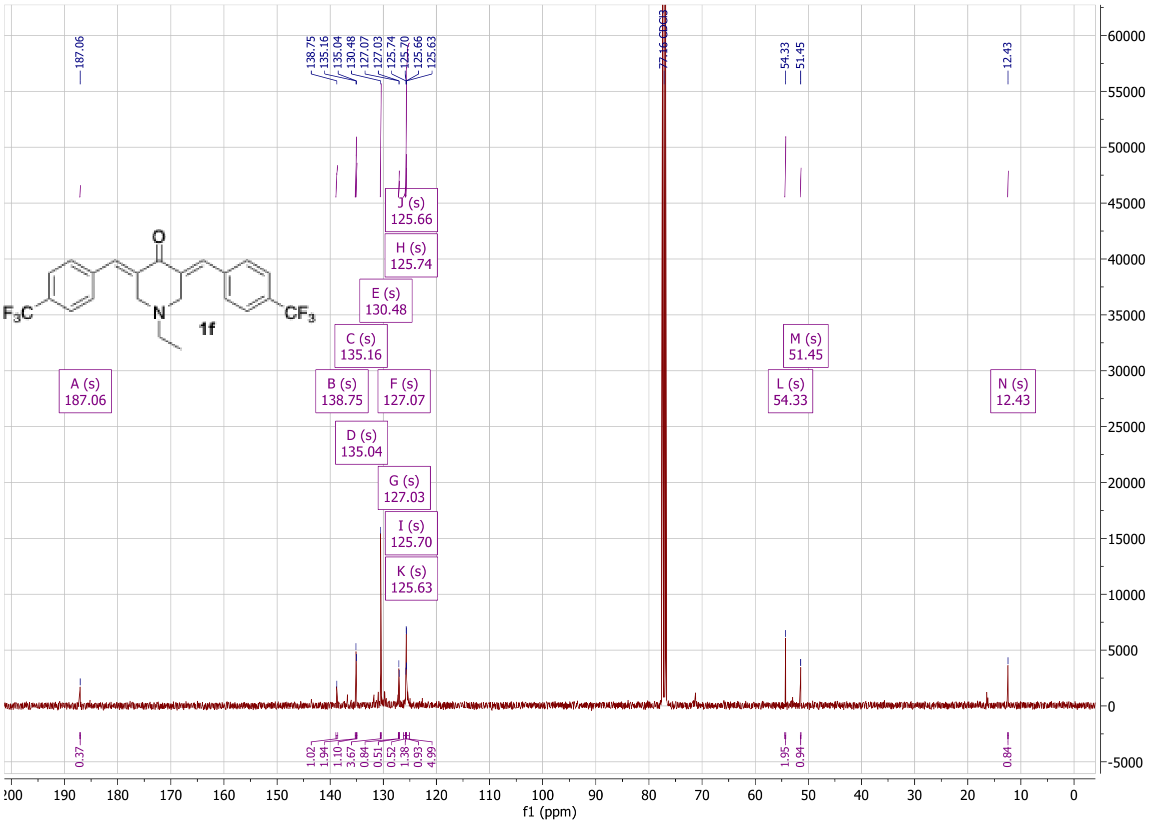

^{1}H NMR for 2a

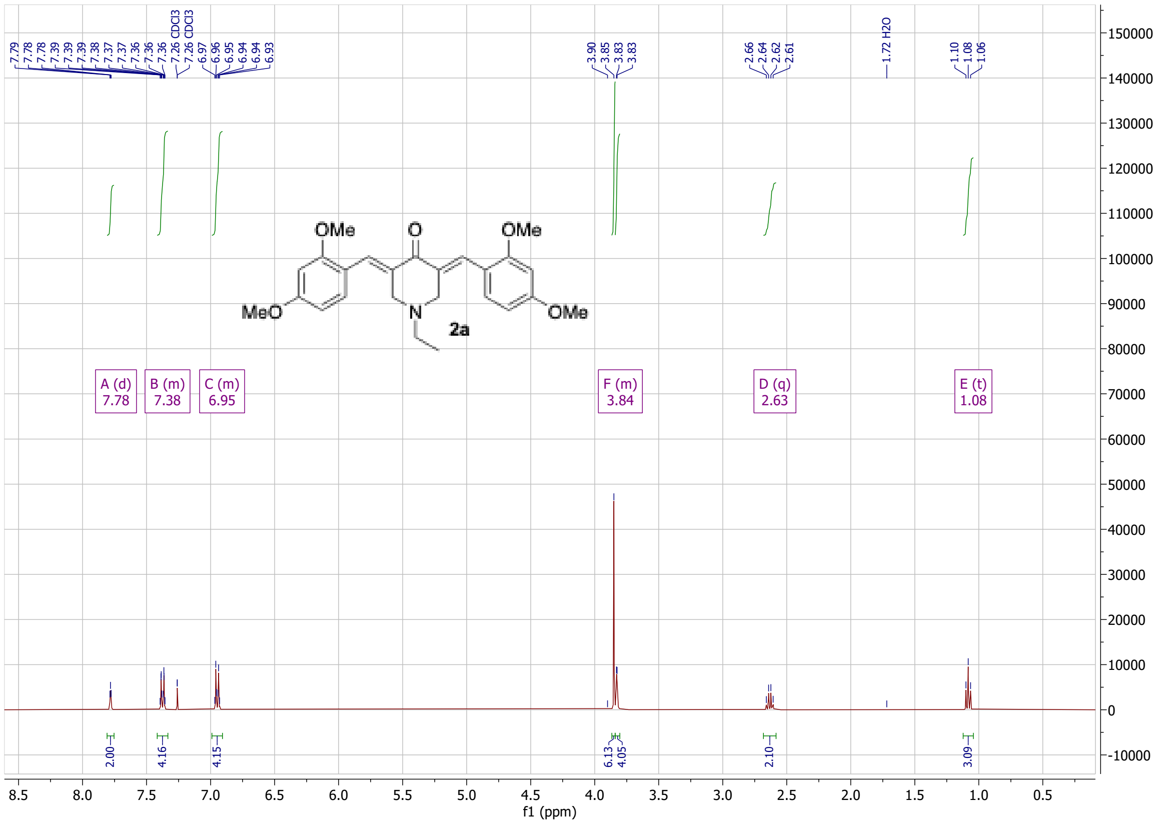

^{13}C NMR for 2a

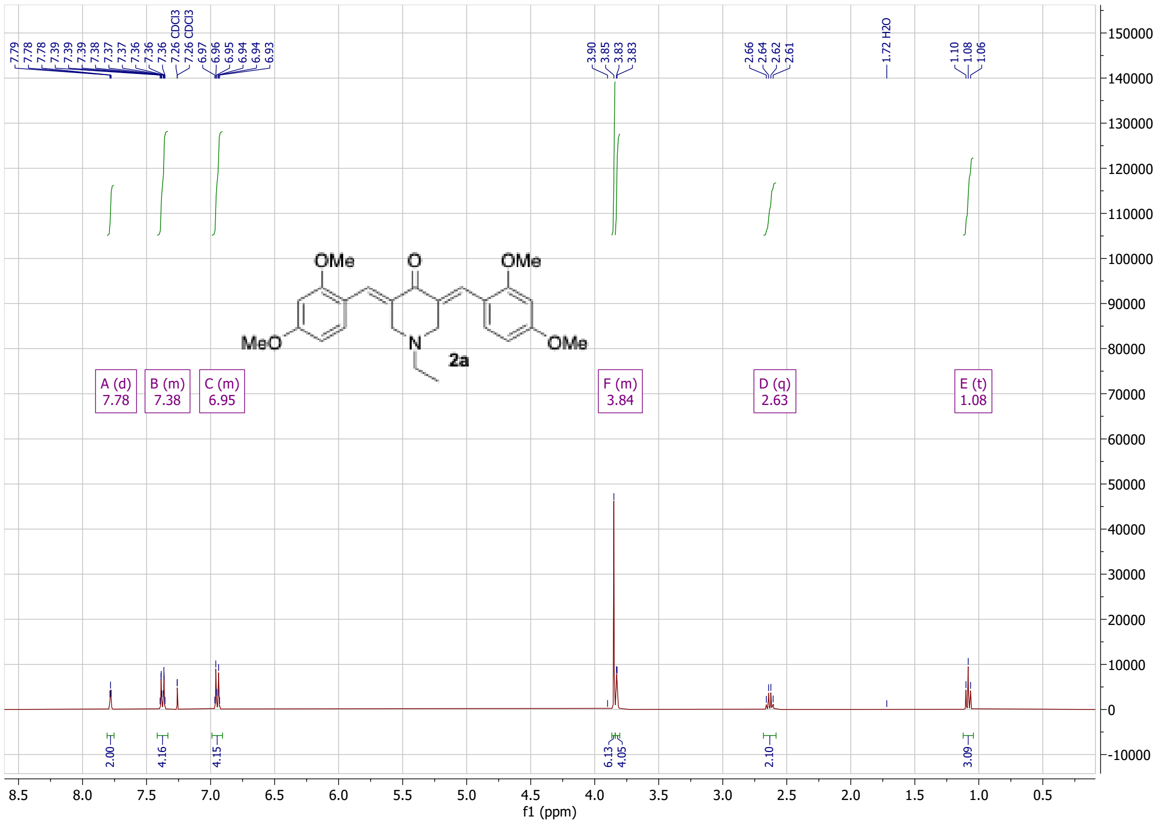

^{1}H NMR for 2g

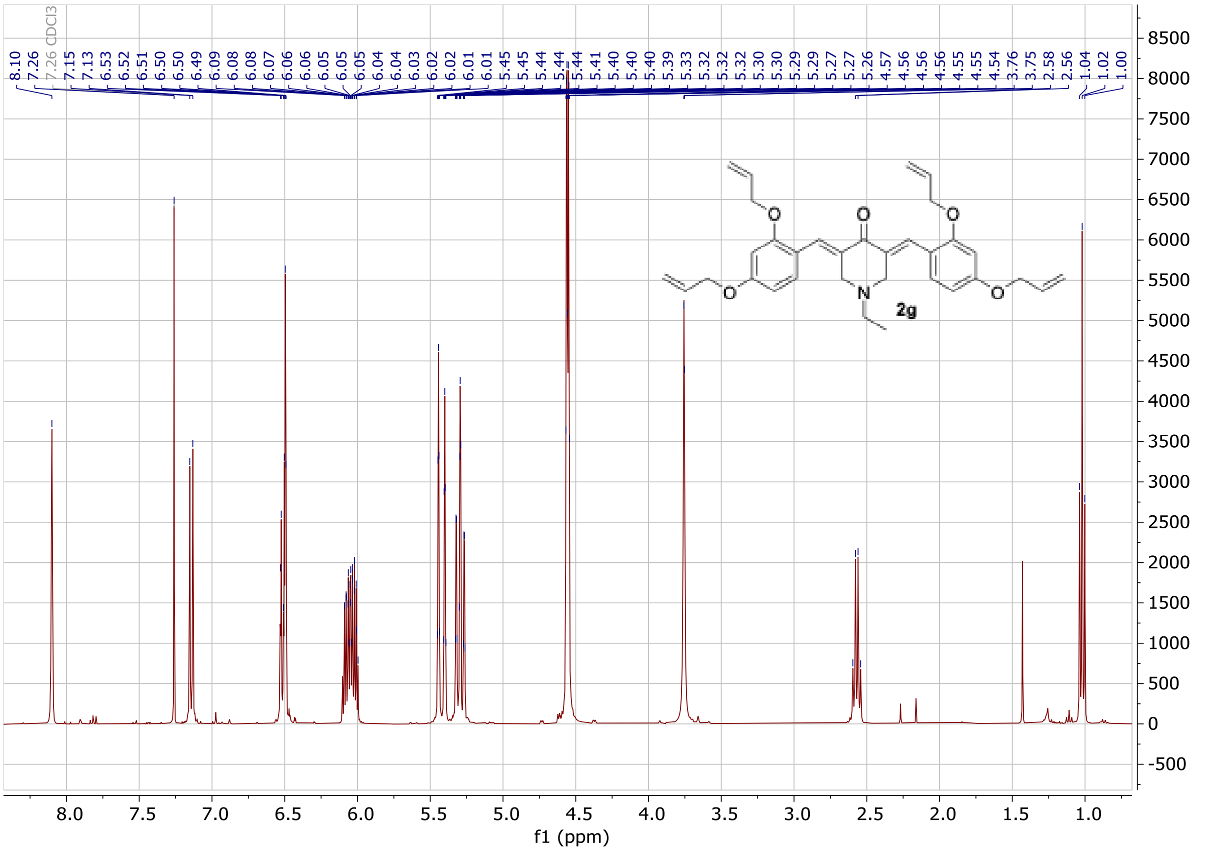

^{13}C NMR for 2g

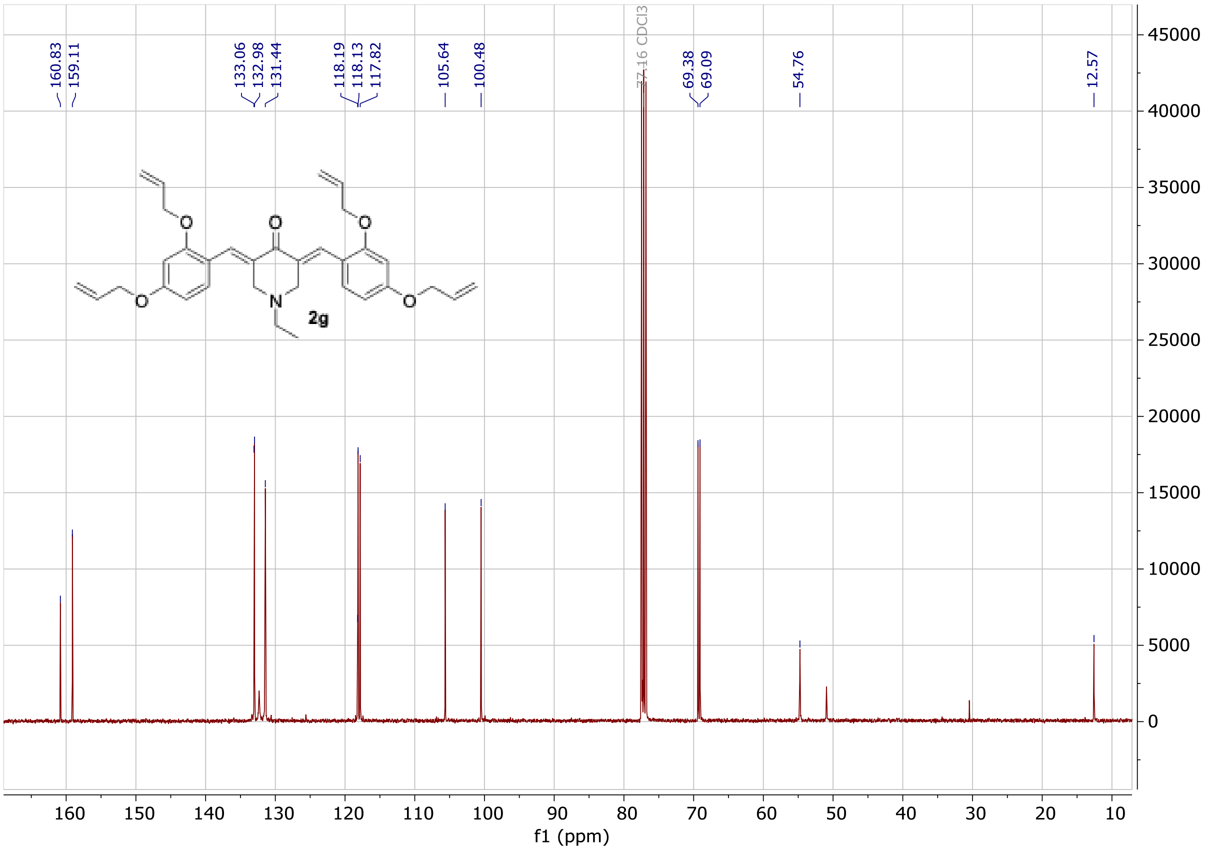

^{1}H NMR for 2h

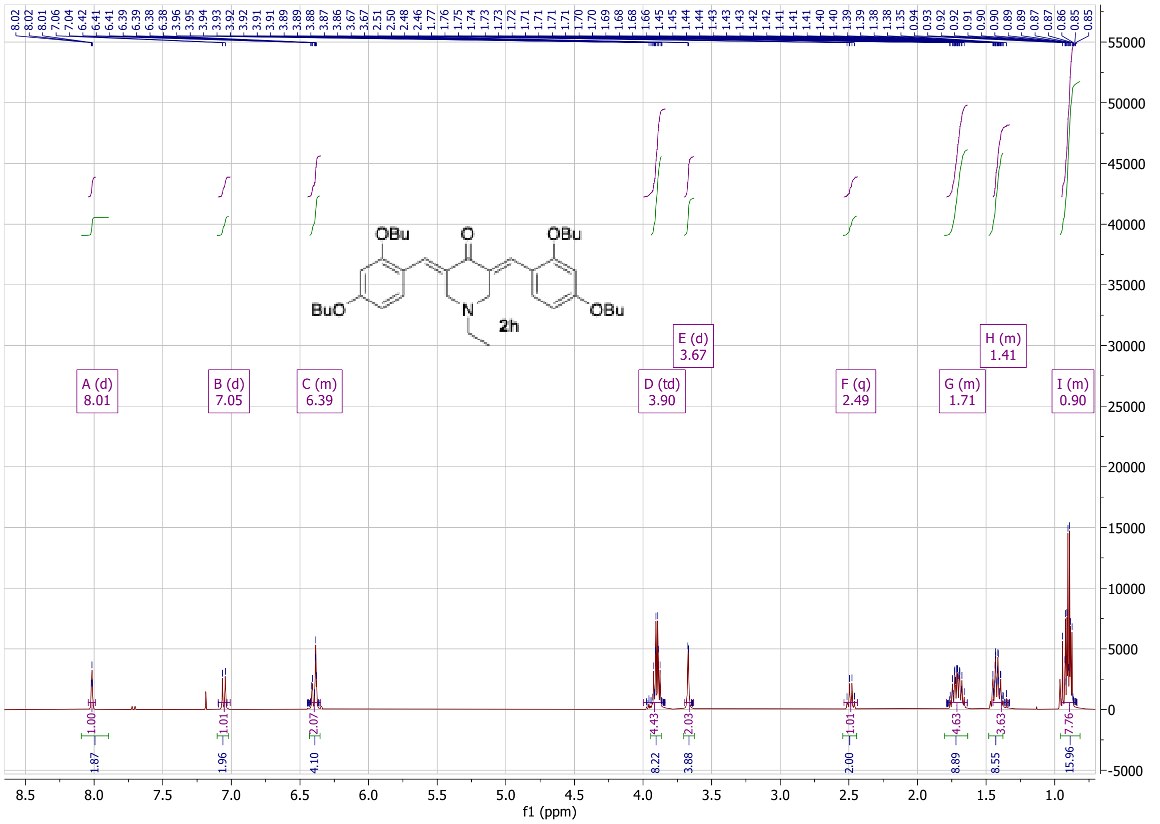

¹³C NMR for 2h

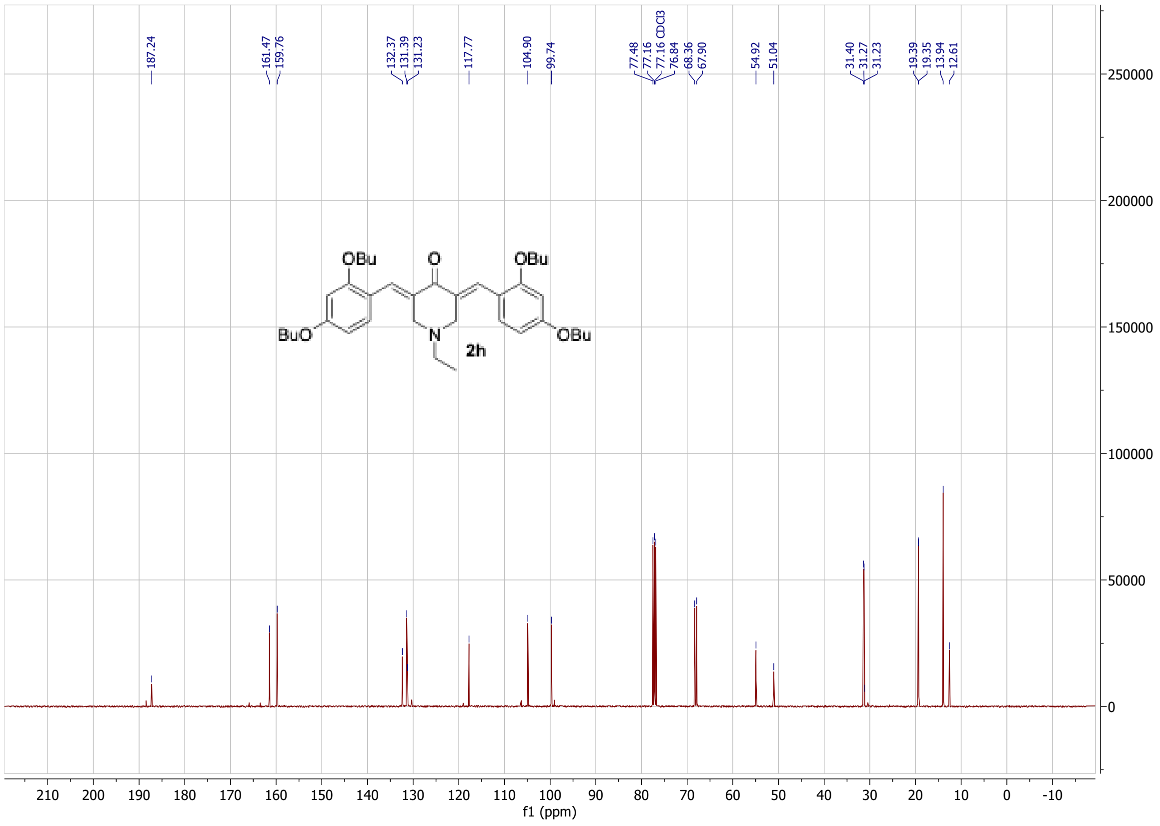

¹H NMR for 2i

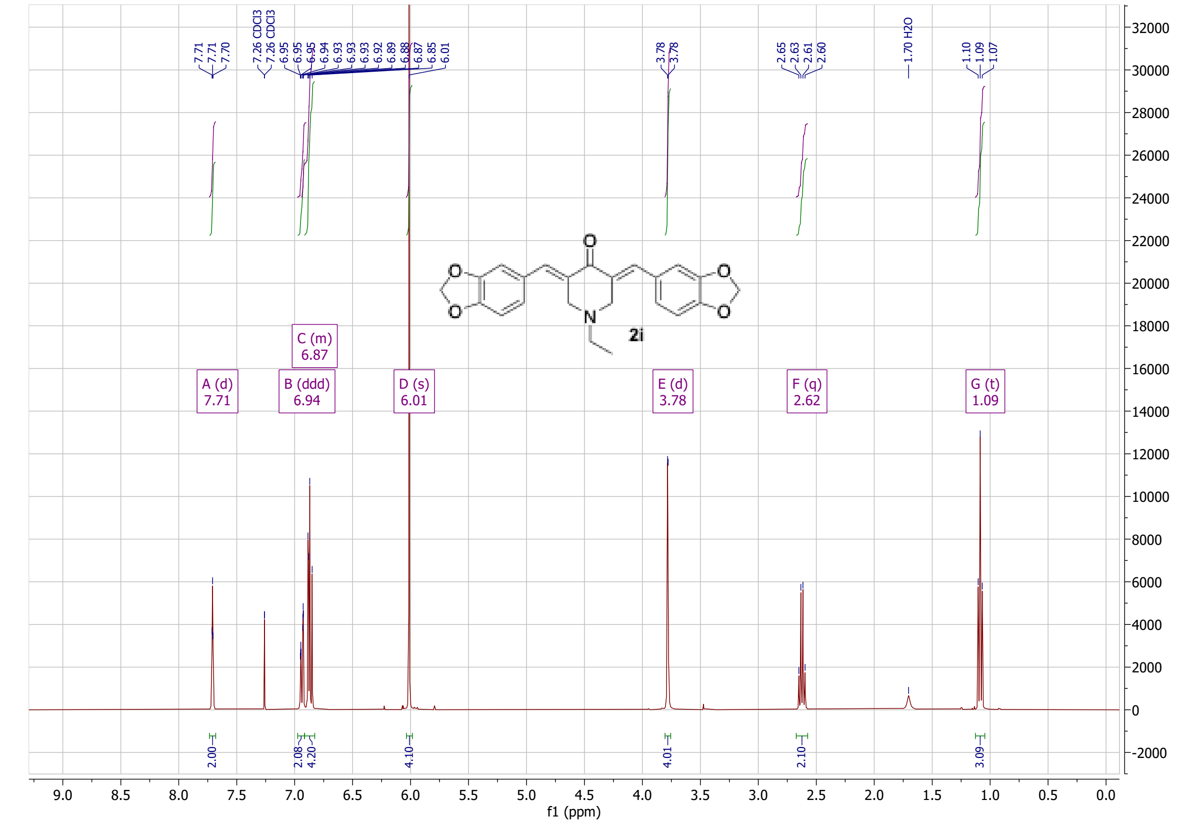

^{13}C NMR for 2i

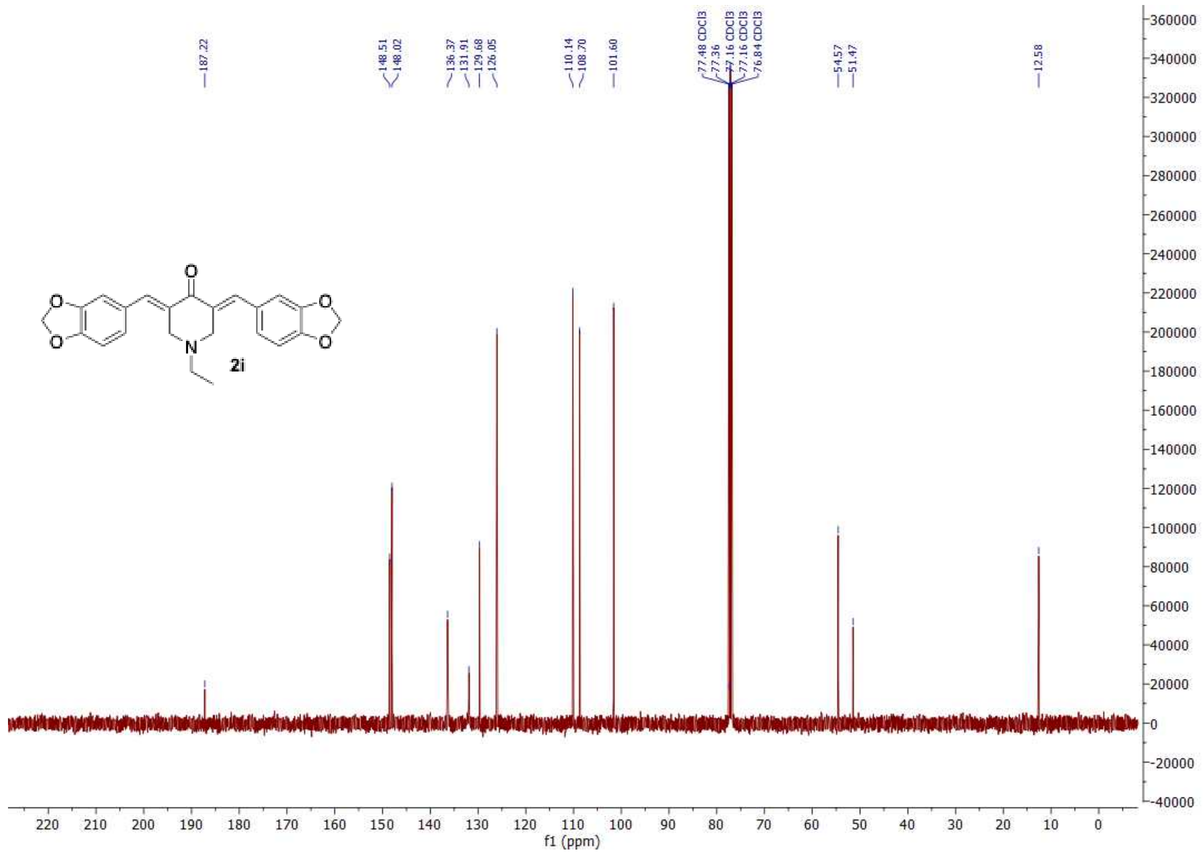

^{1}H NMR for 2j

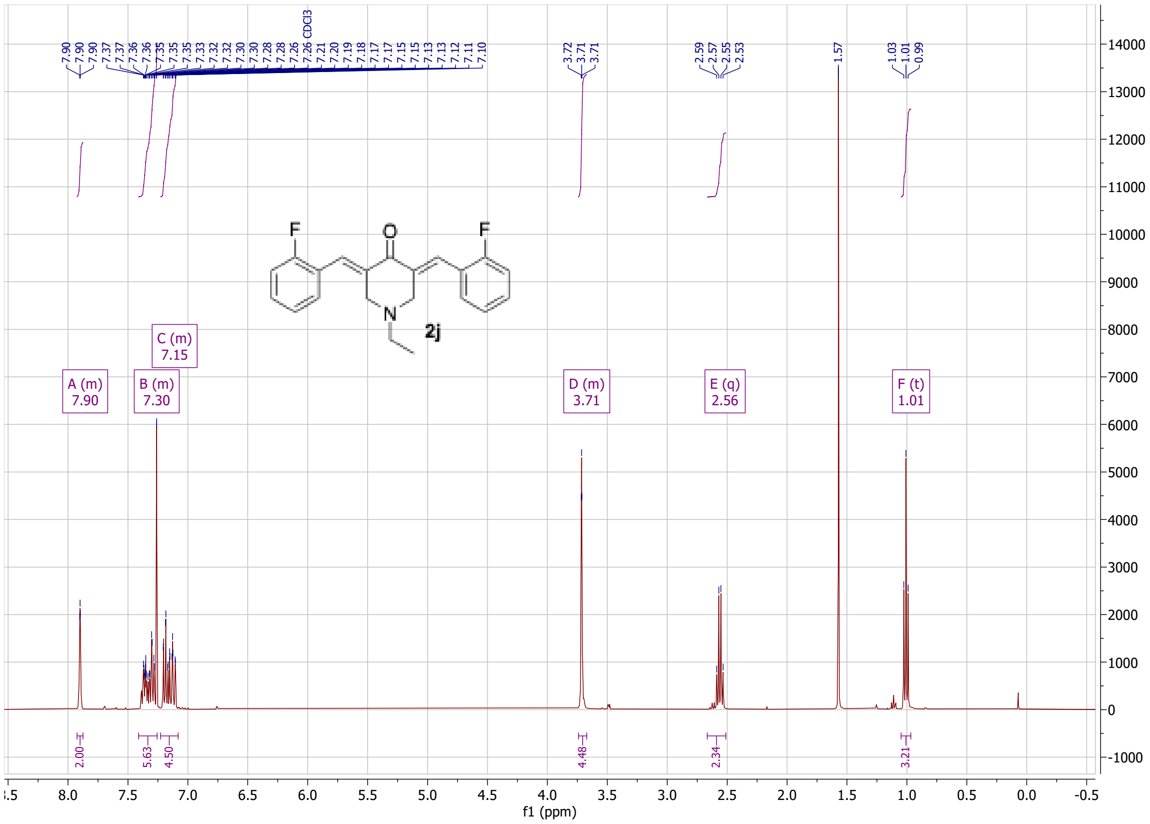

^{13}C NMR for 2j

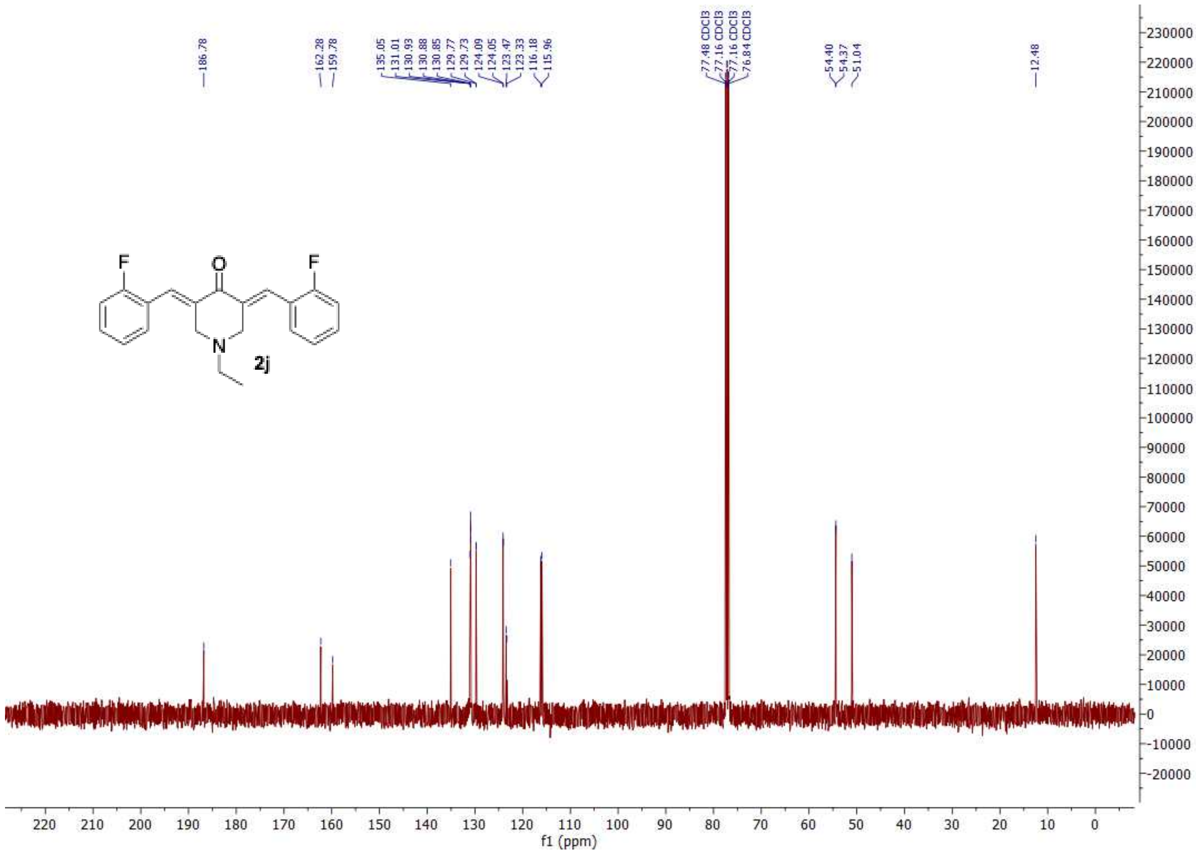

^{1}H NMR for 2k

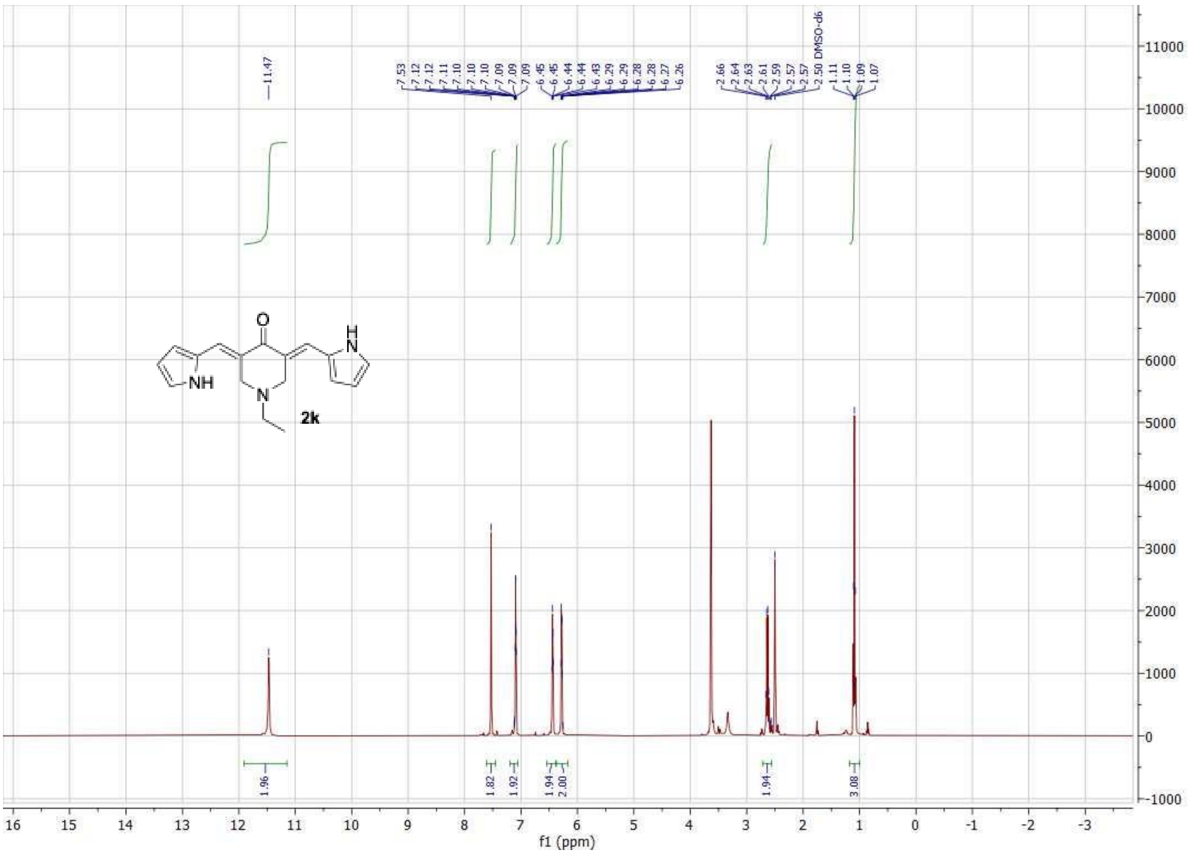

^{13}C NMR for 2k

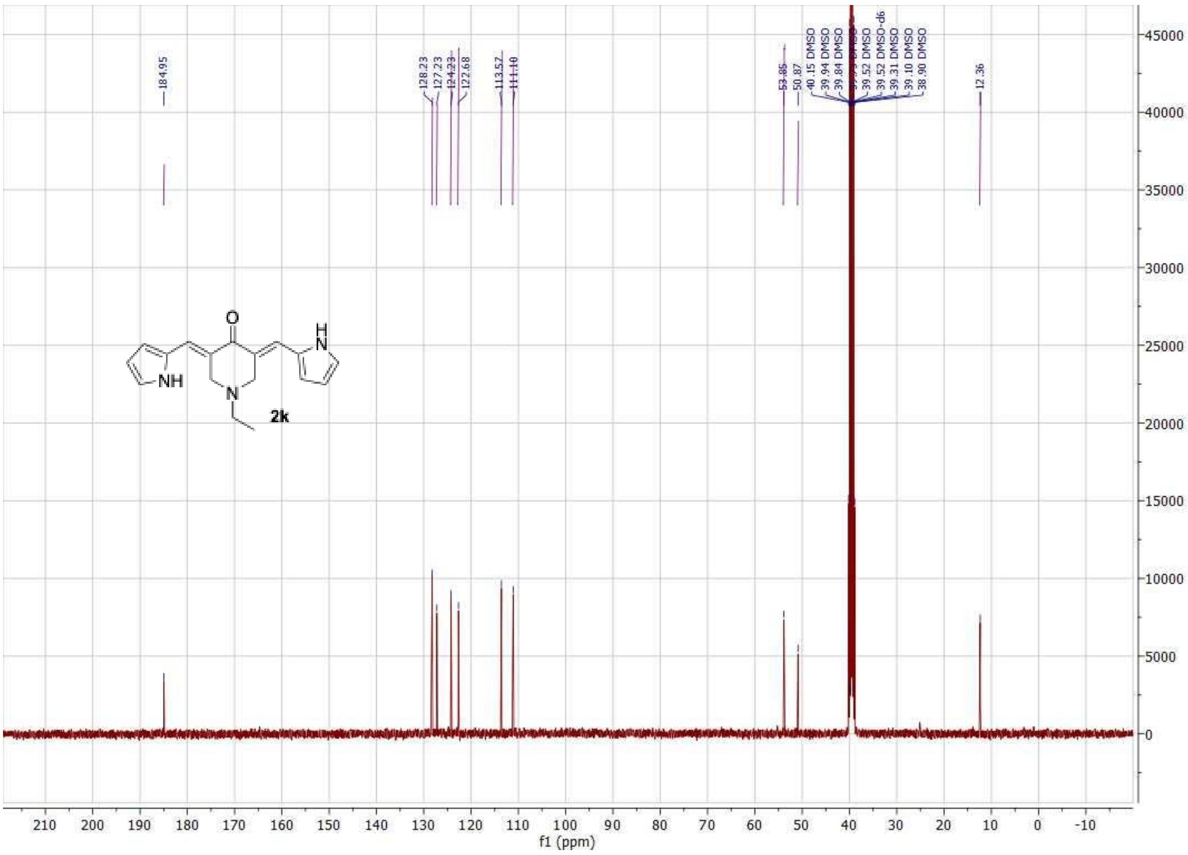

^{1}H NMR for 2l

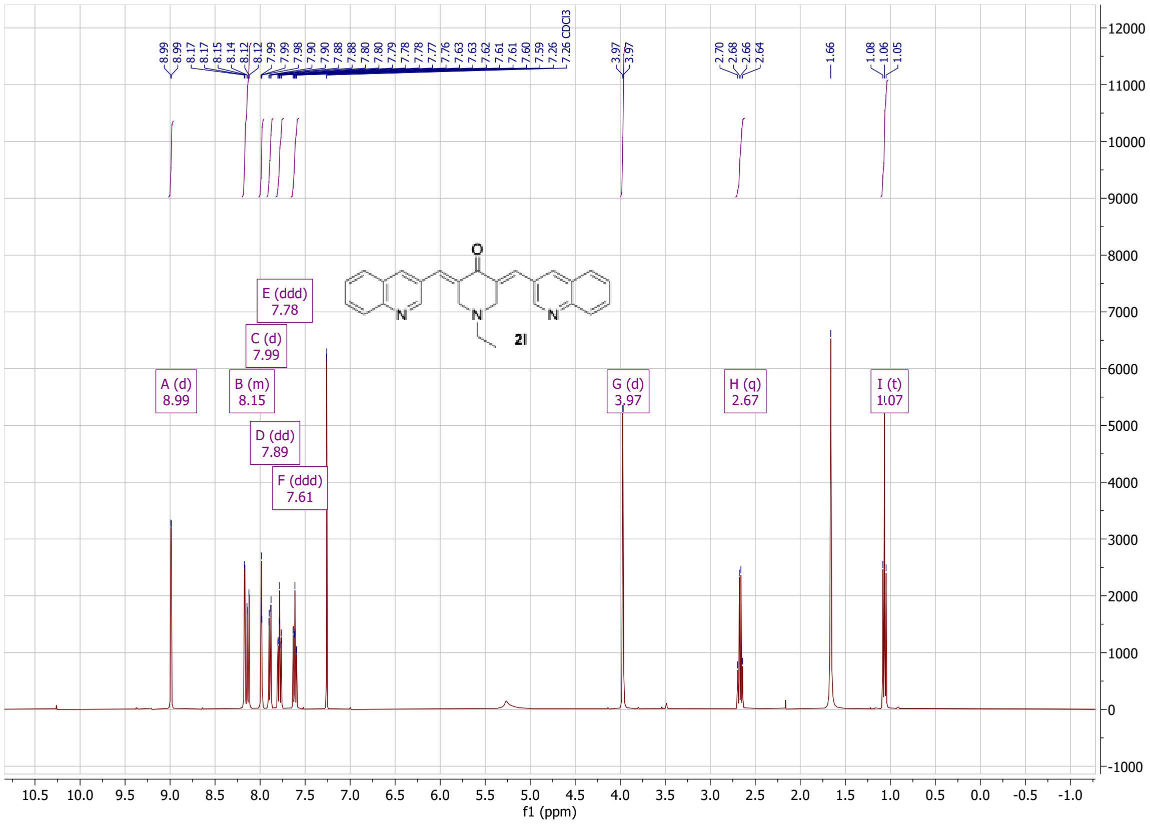

^{13}C NMR for 2l

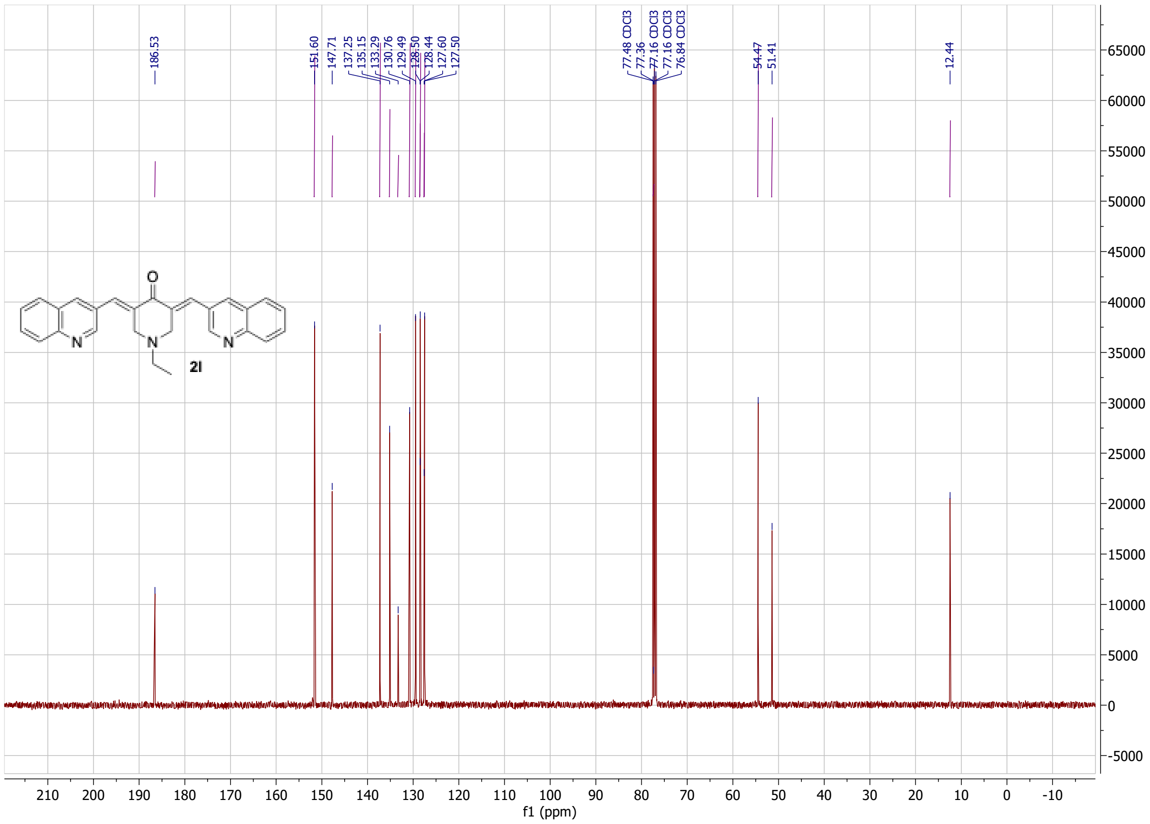

^{1}H NMR for 2m

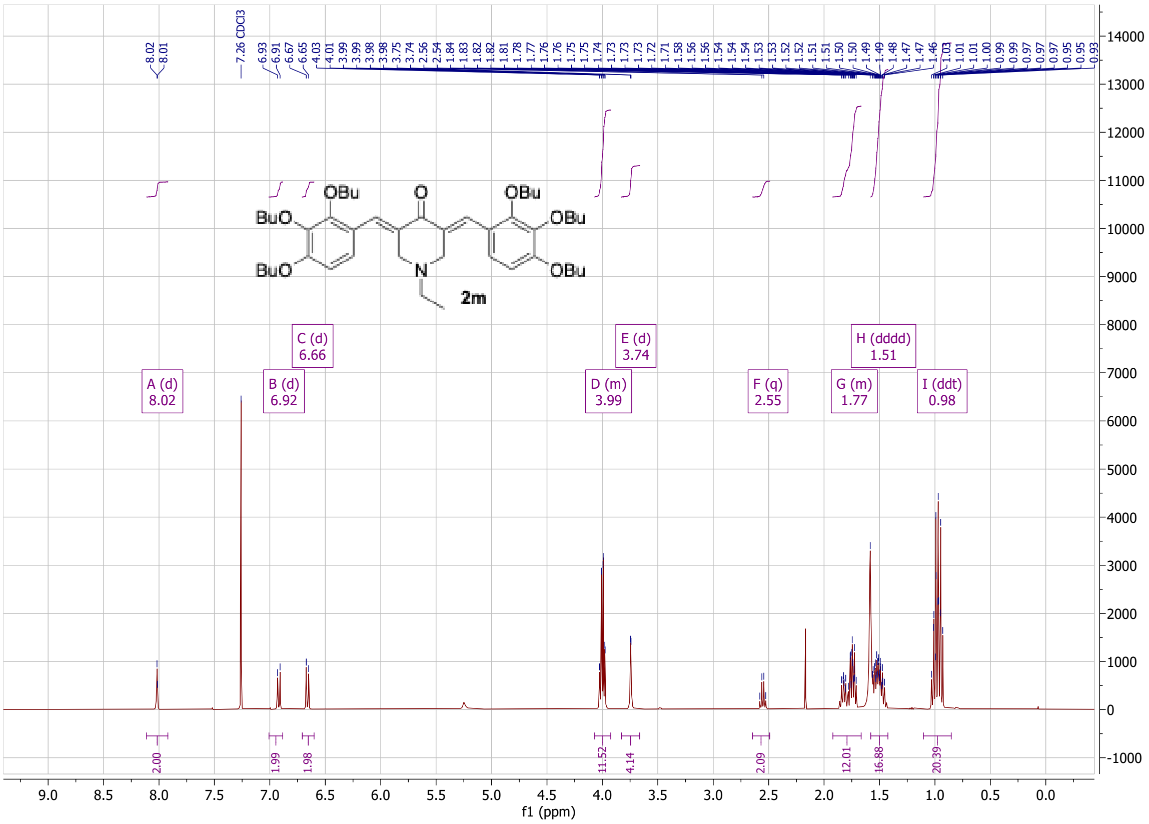

^{13}C NMR for 2m

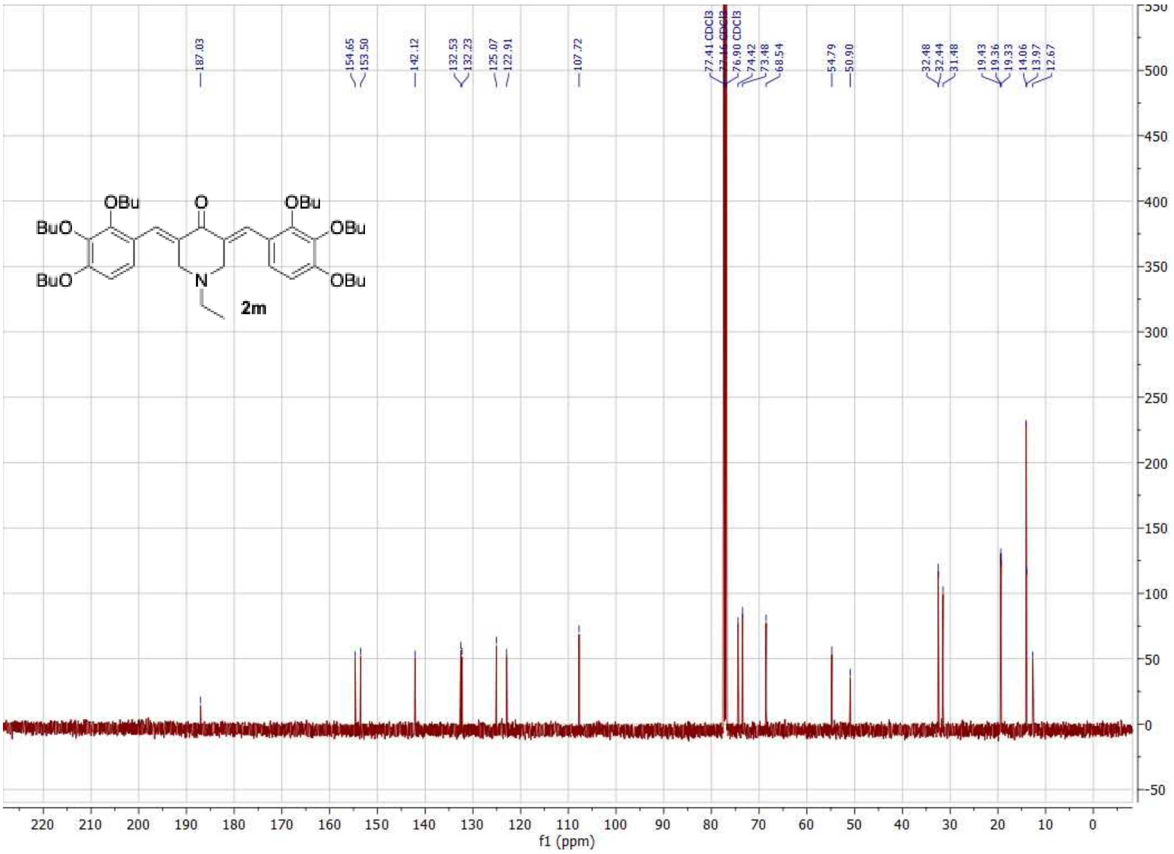

^{1}H NMR for 2n

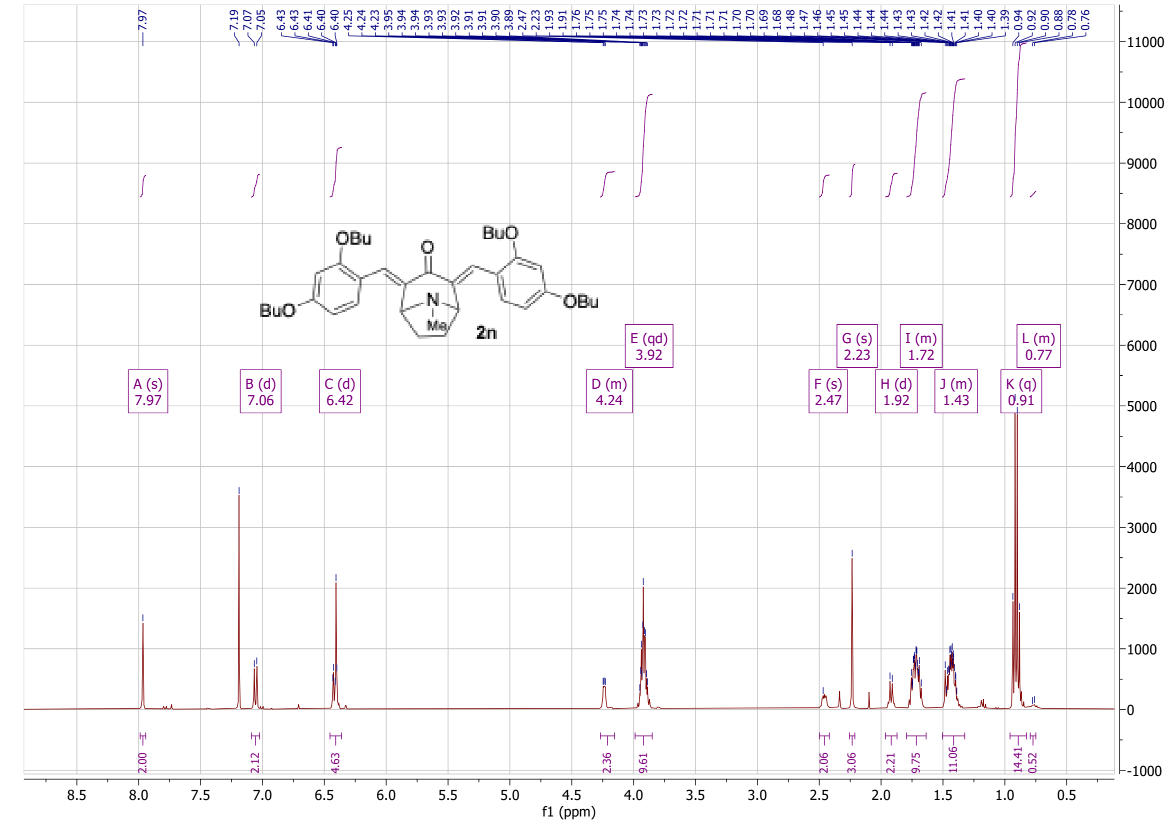

APT NMR sequence for 2n (CH and CH3 : down ; C and CH2 up)

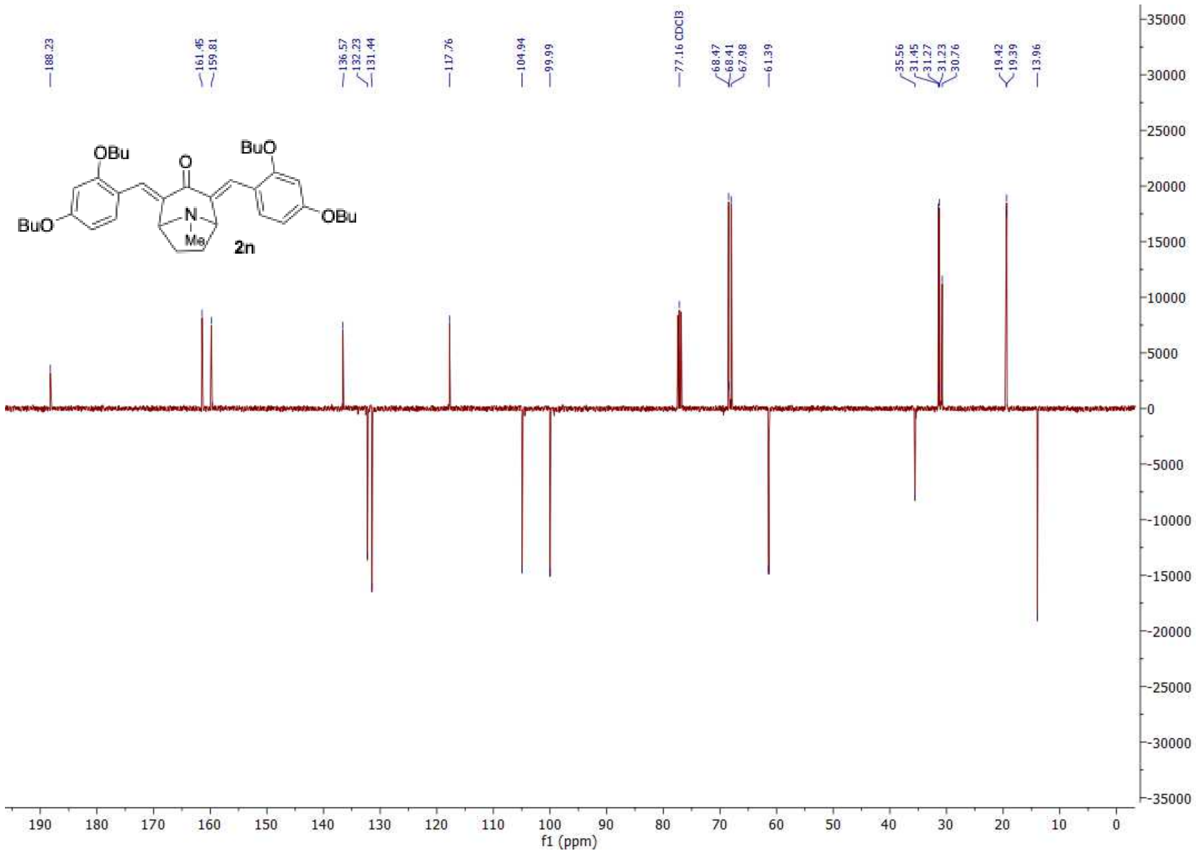

^{1}H NMR for 2o

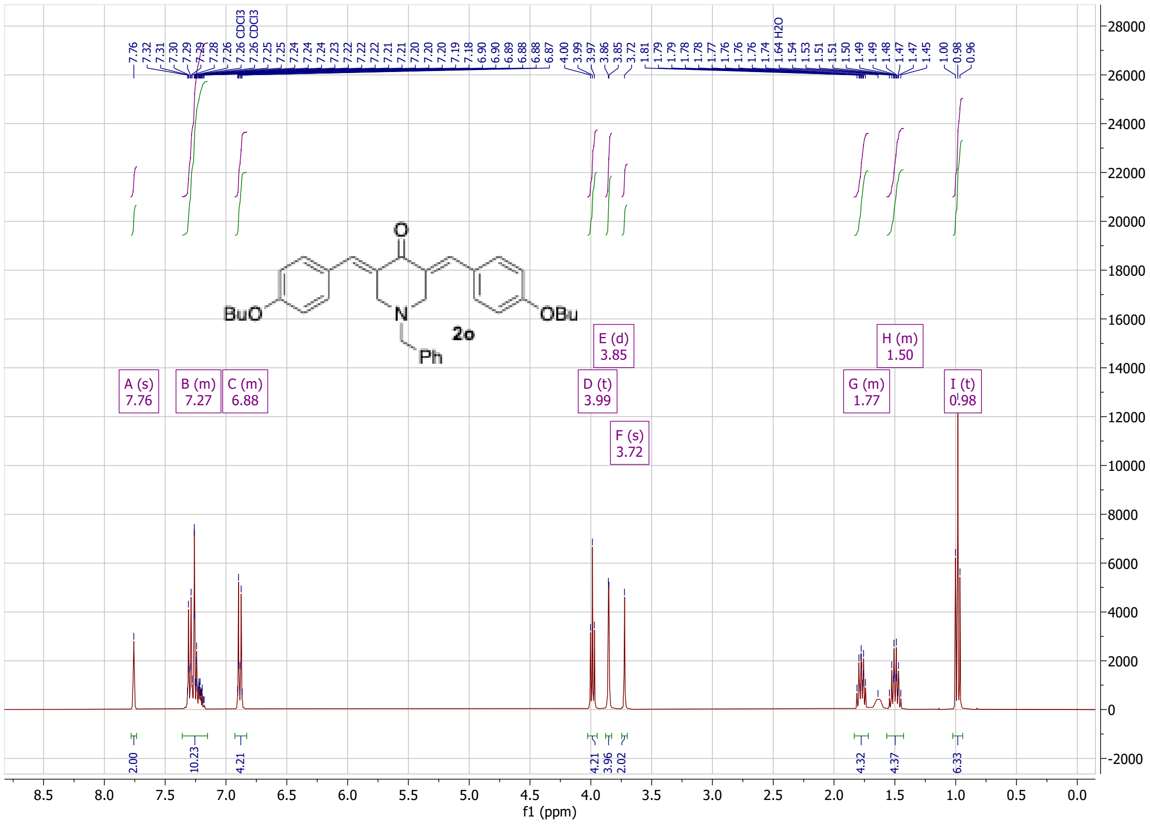

^{13}C NMR for 2o

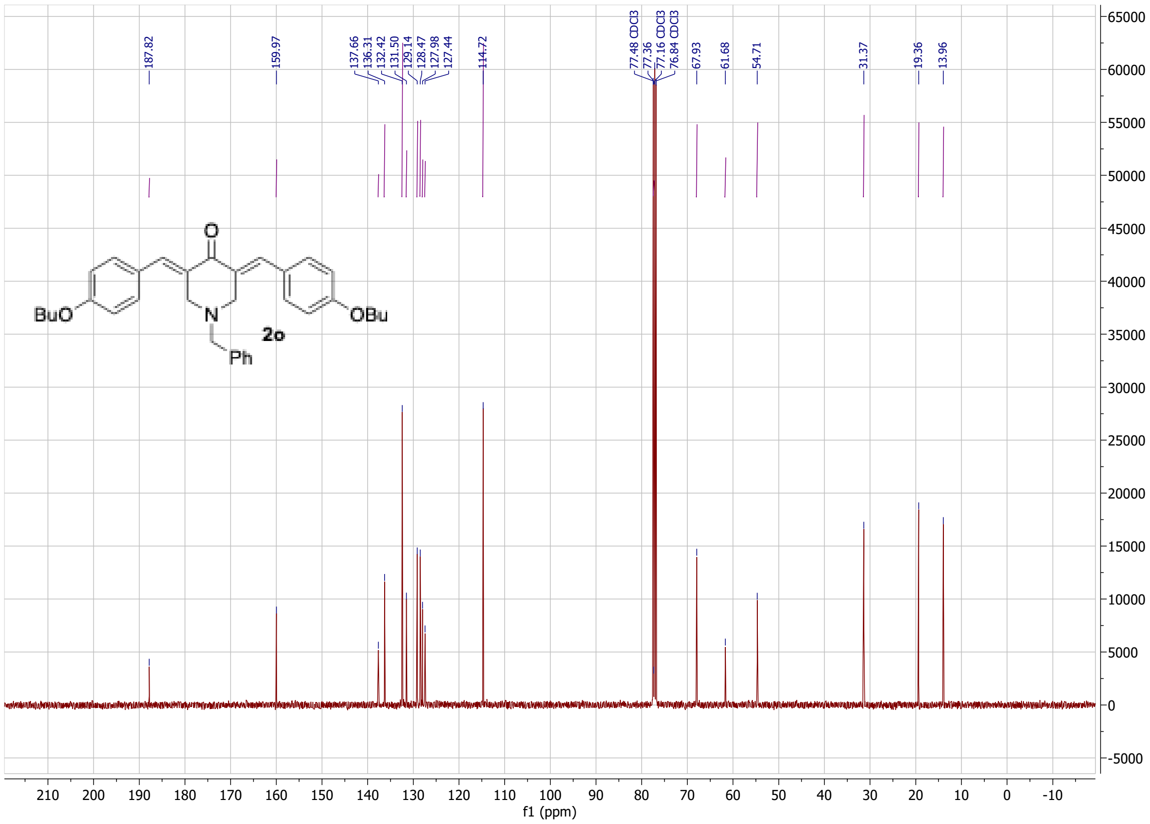

^{1}H NMR for 3a

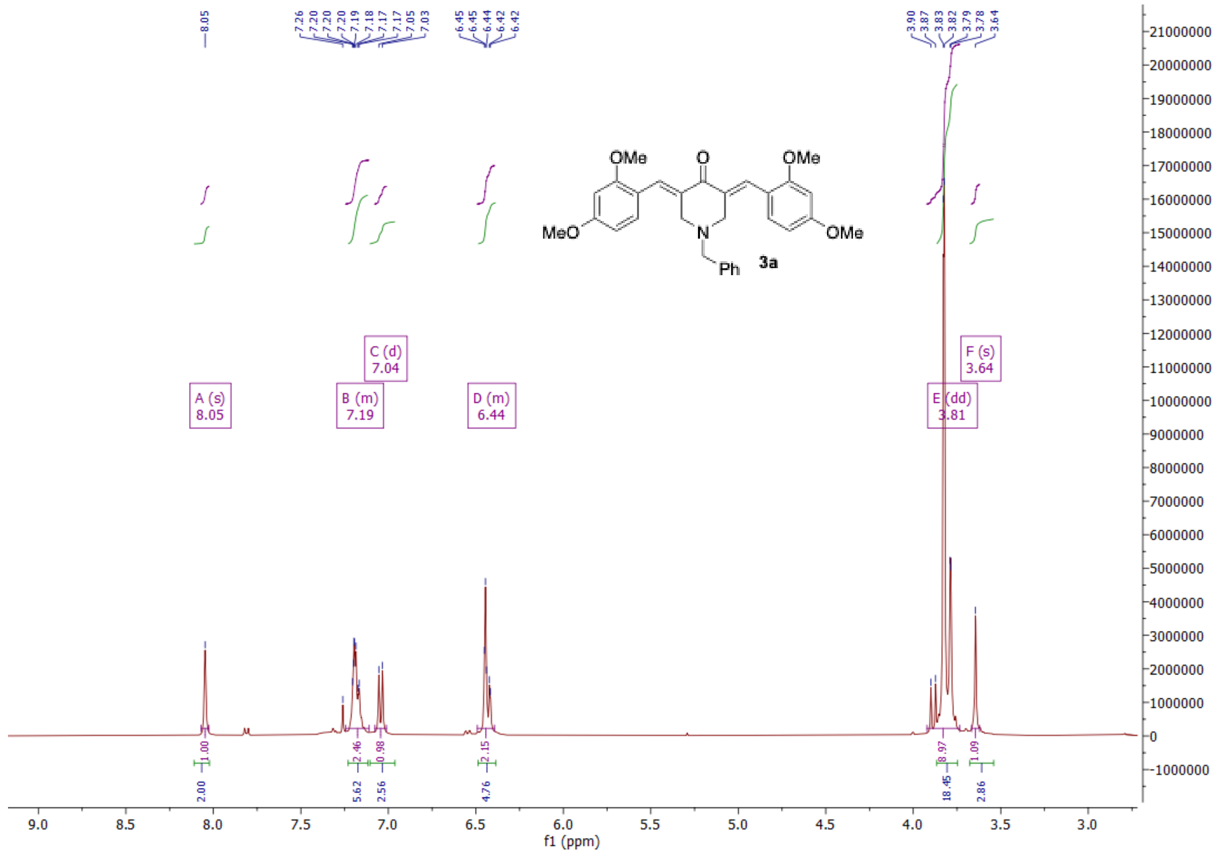

^{13}C NMR for 3a

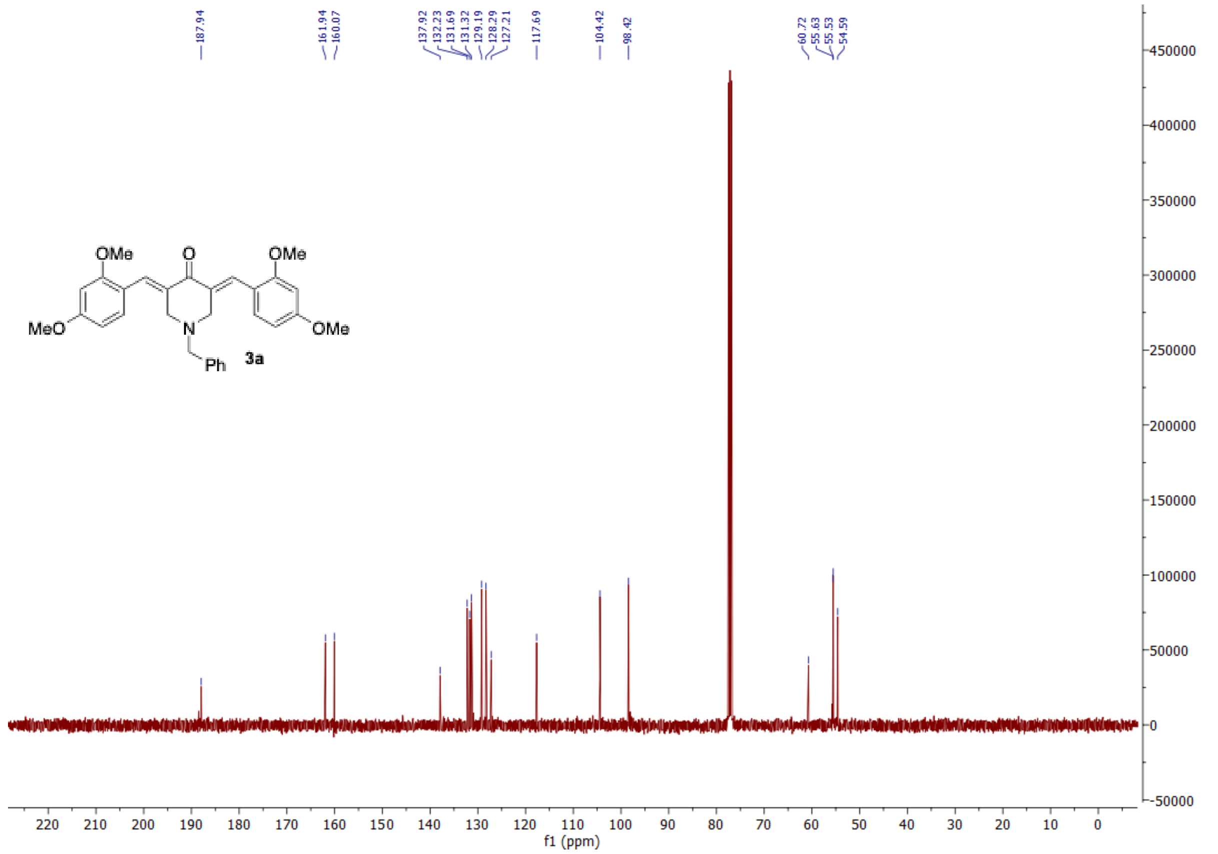

^{1}H NMR for 3h

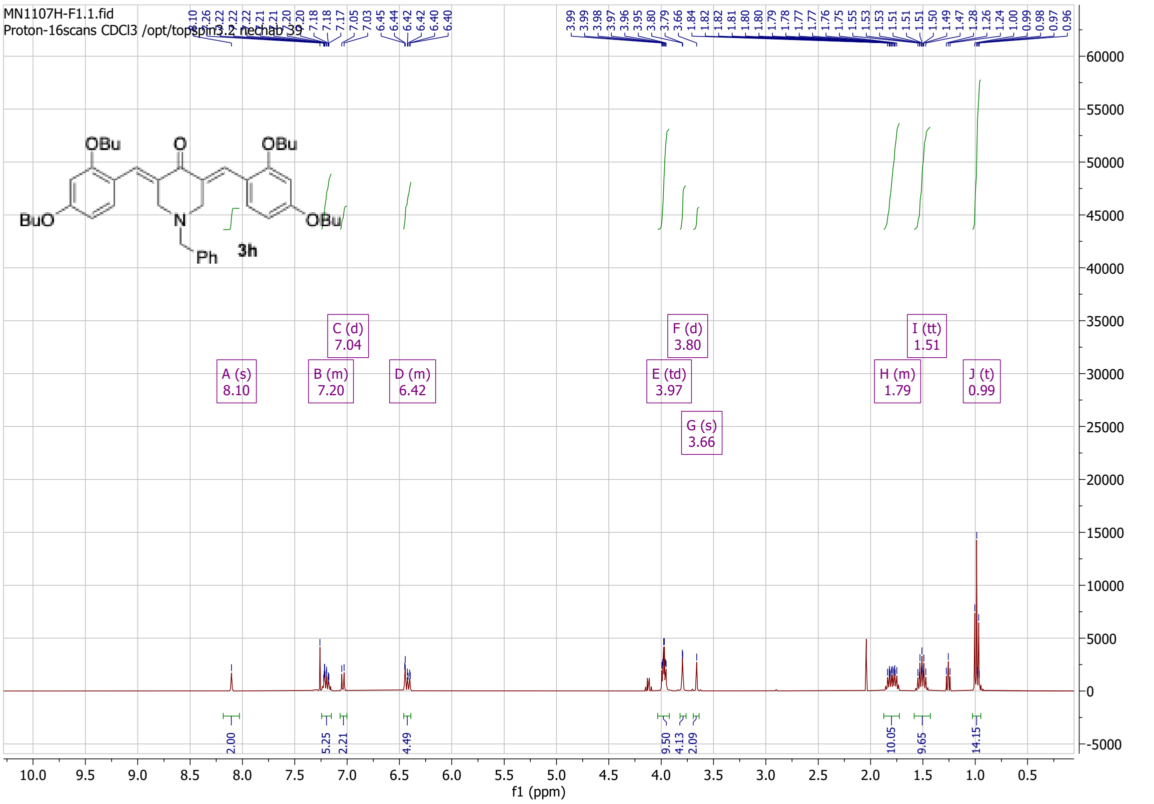

^{13}C NMR for 3h

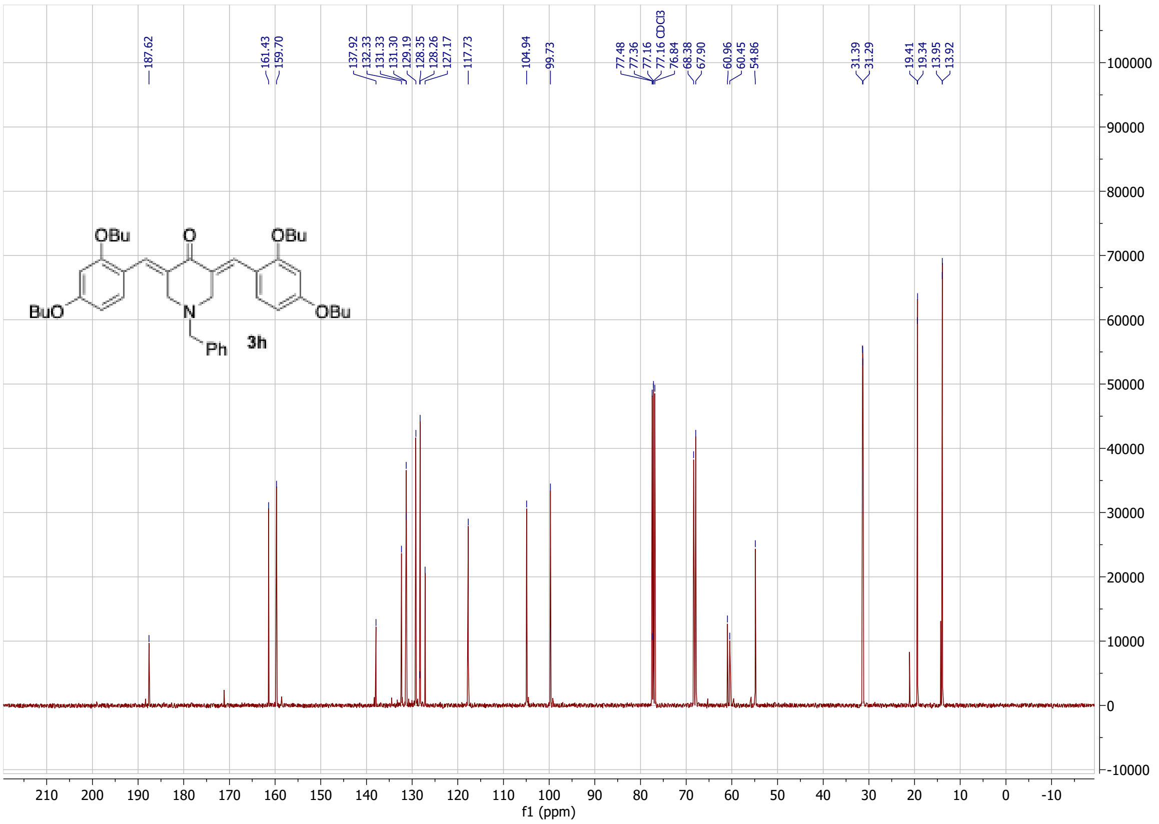

^{1}H NMR for 3iso-h

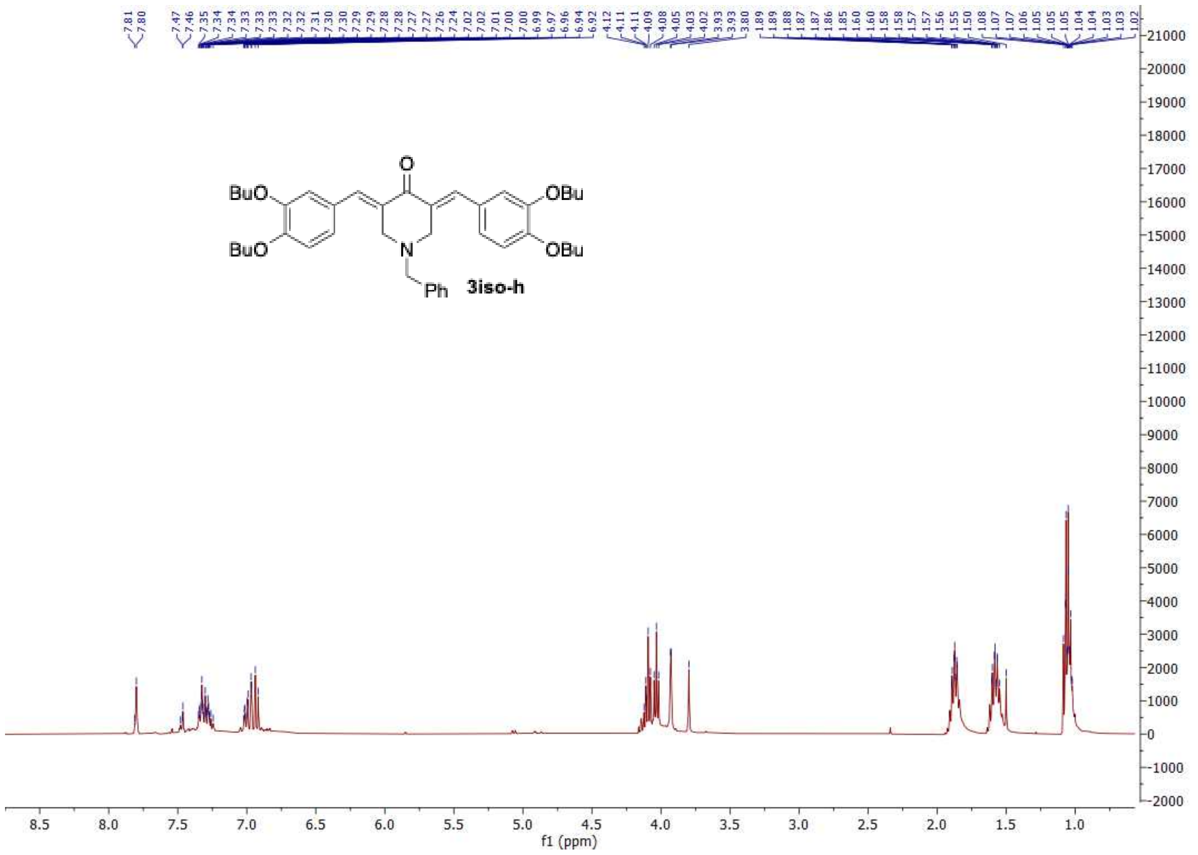

^{13}C NMR for 3iso-h

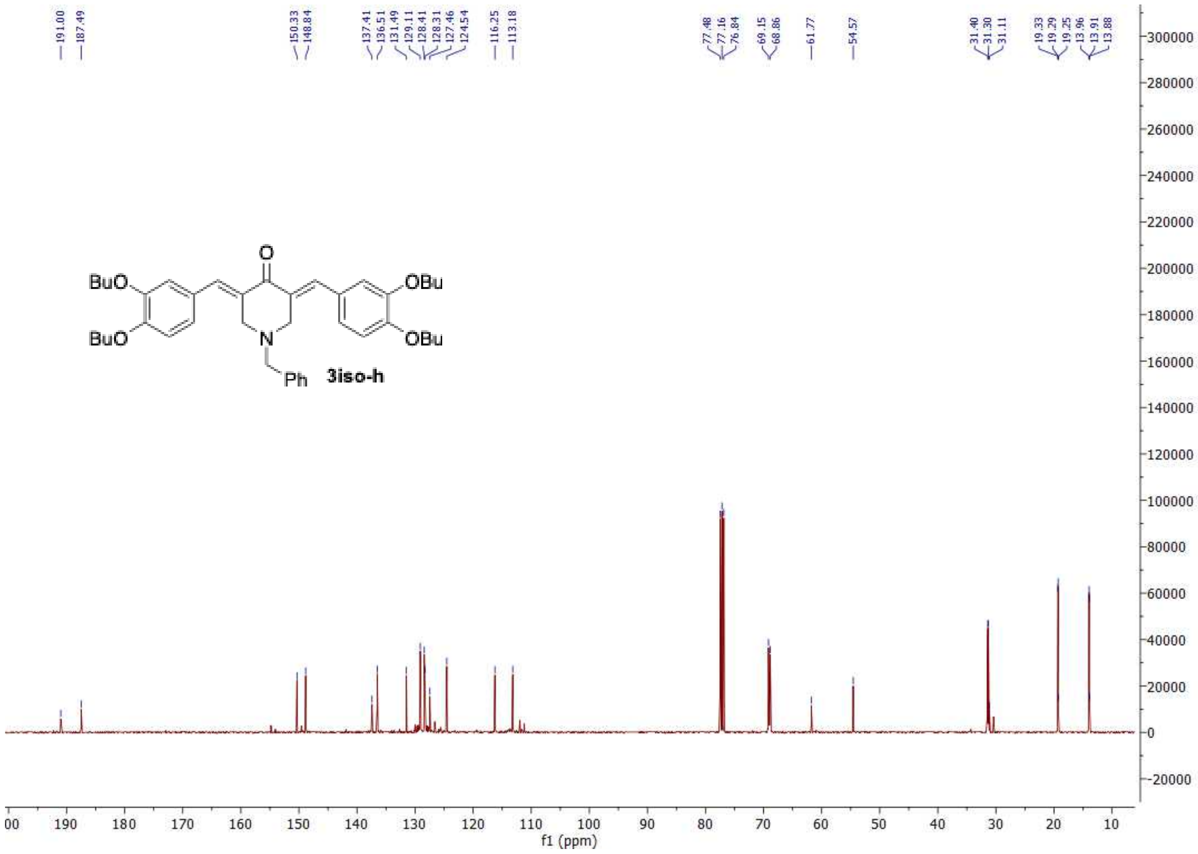

^{1}H NMR for 3j

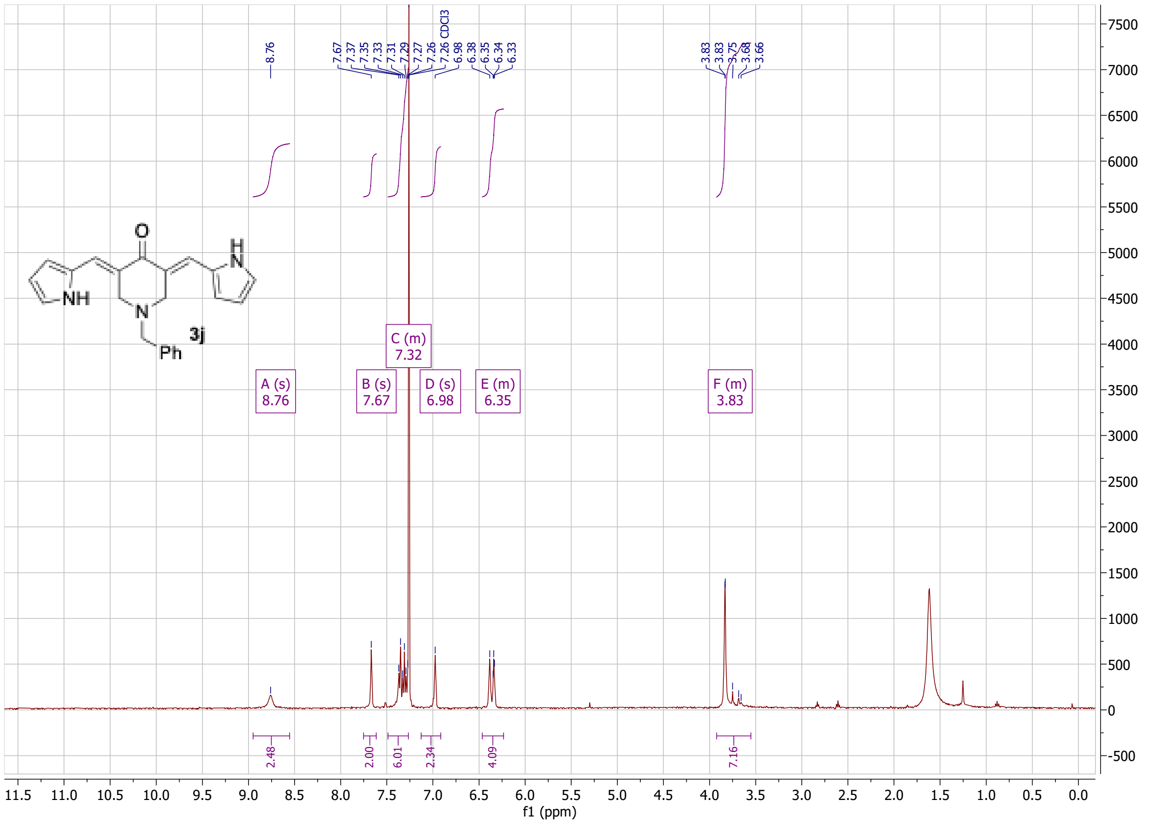

^{1}H NMR for 4h

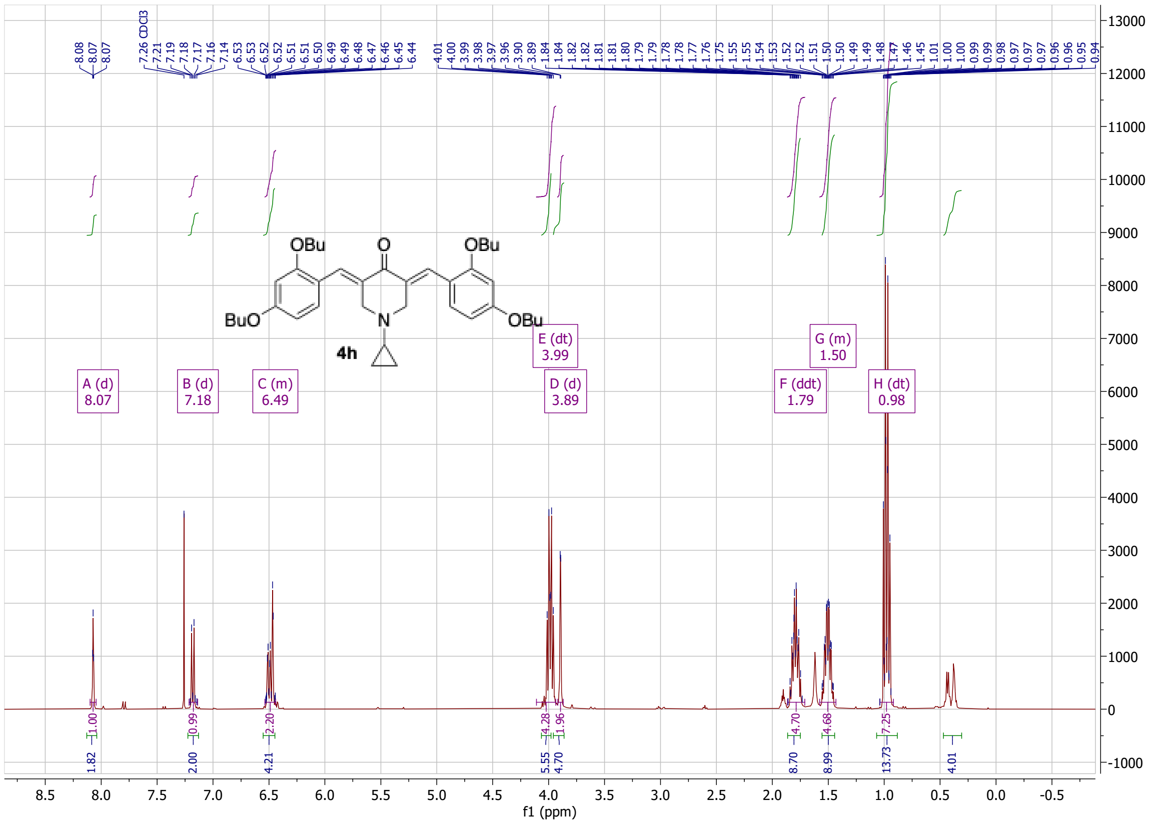

^{13}C NMR for 4h

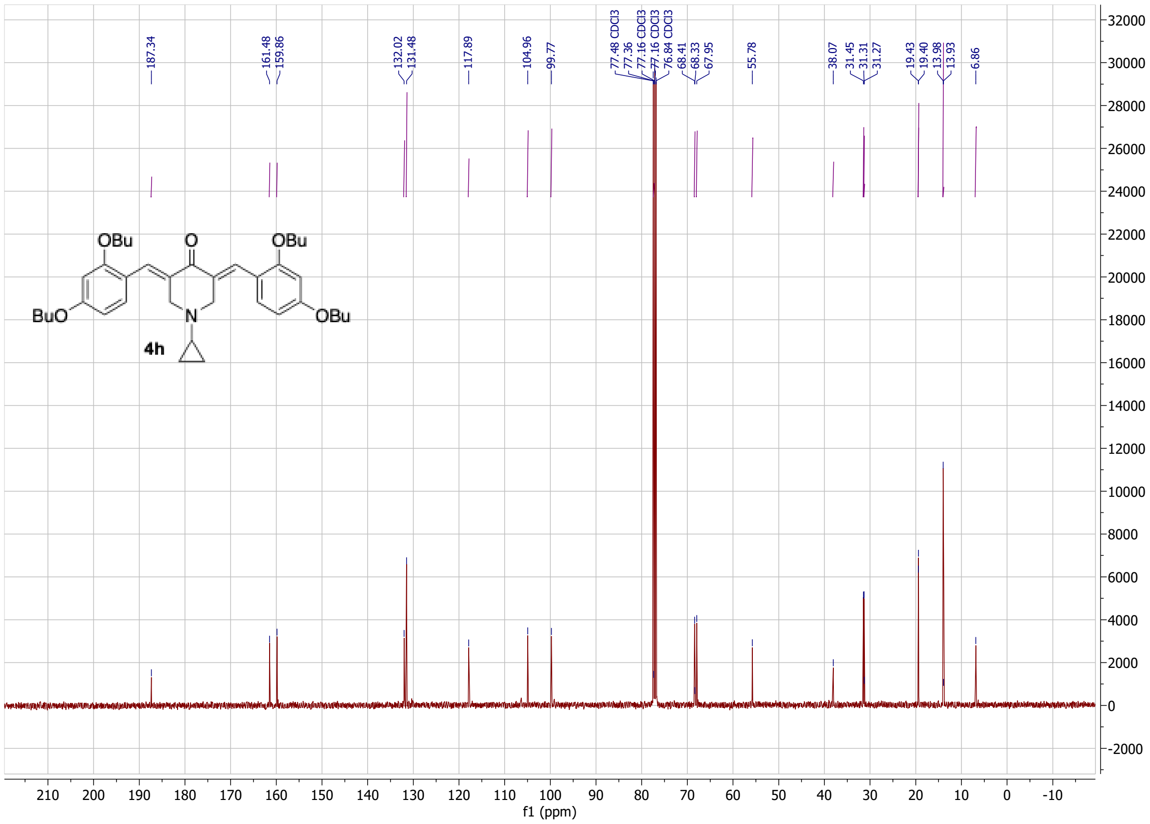

^{1}H NMR for 5h

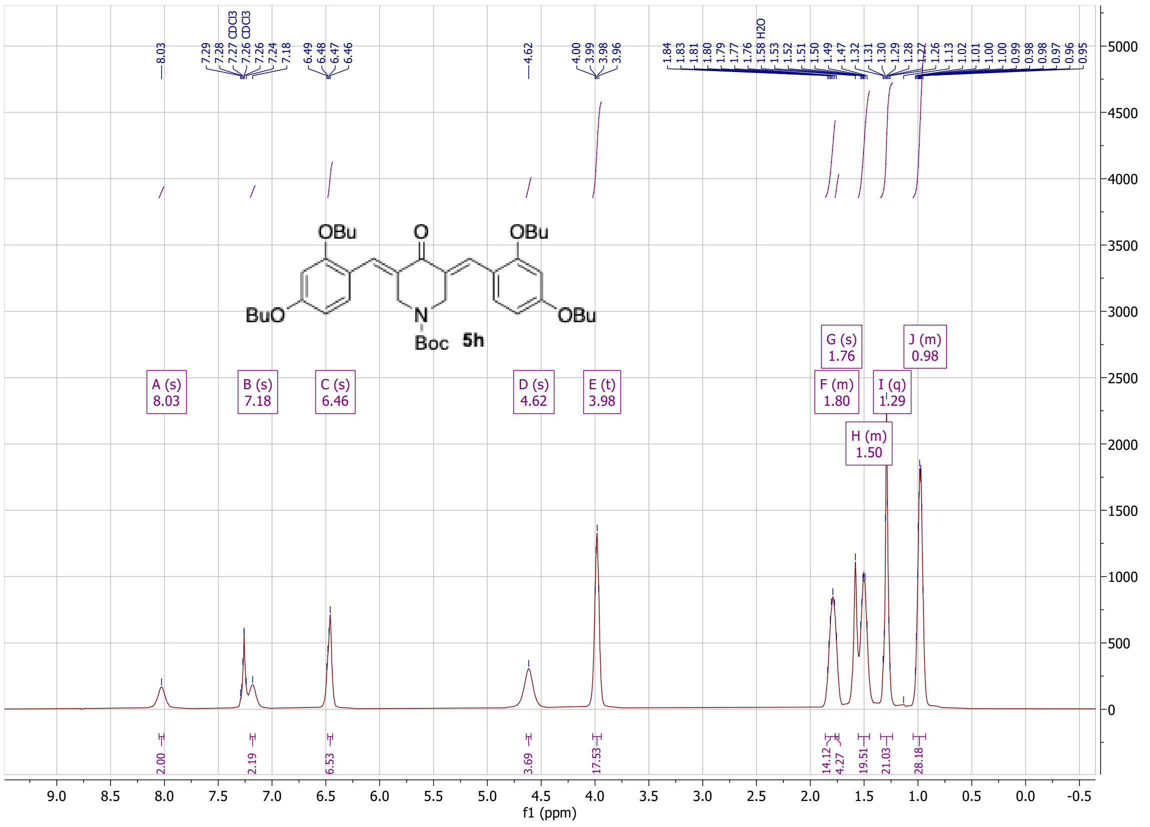

^{13}C NMR for 5h

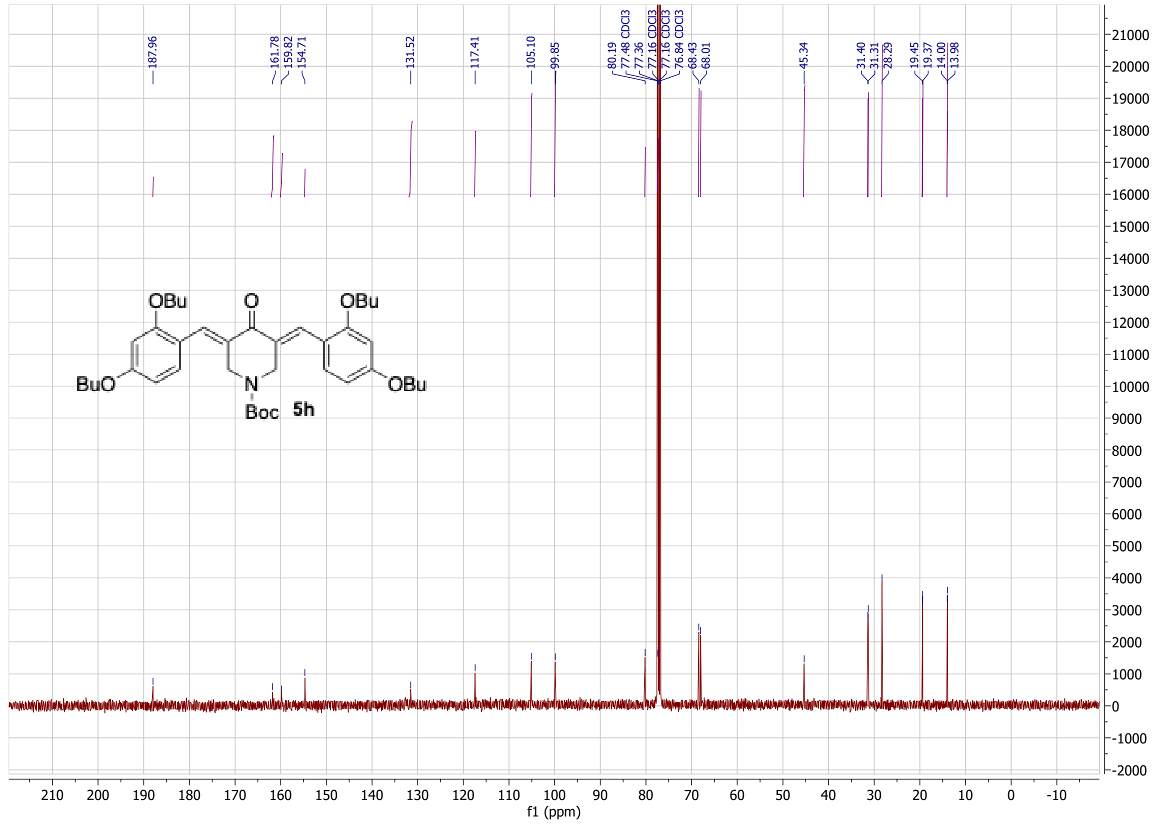

^{1}H NMR for 6h

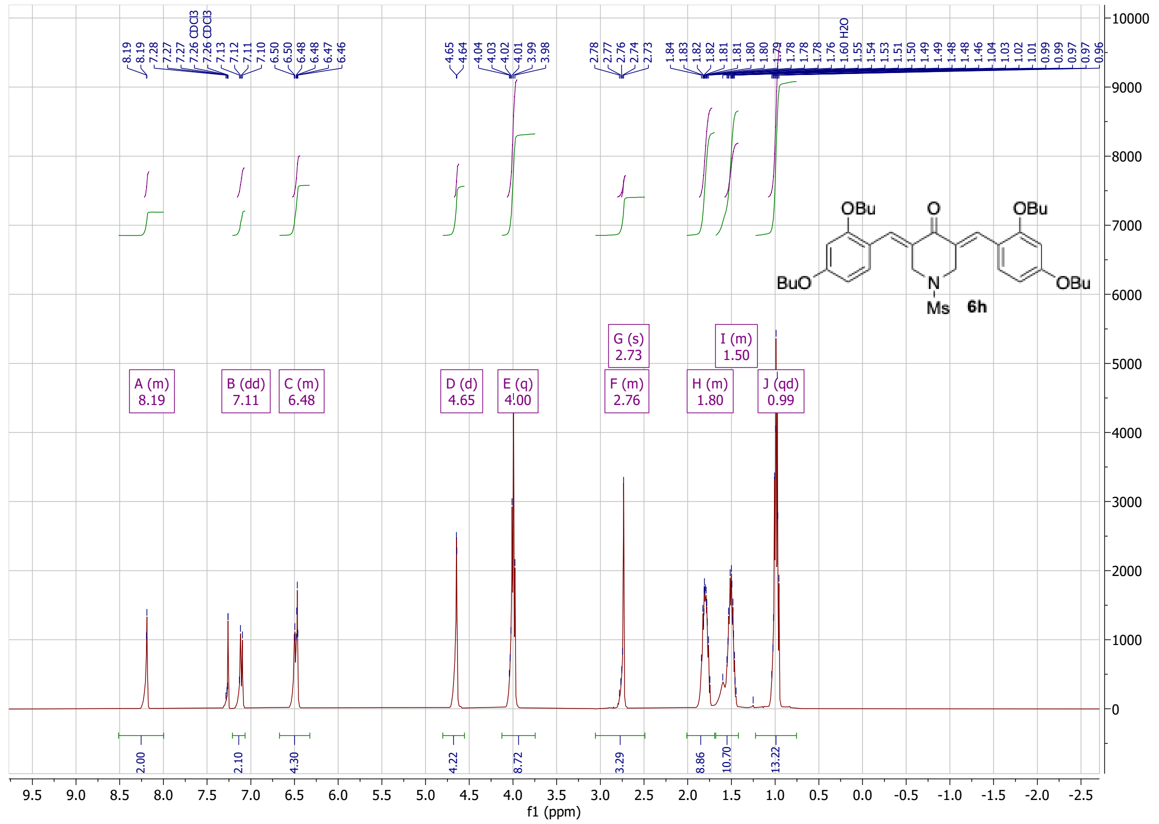

^{13}C NMR for 6h

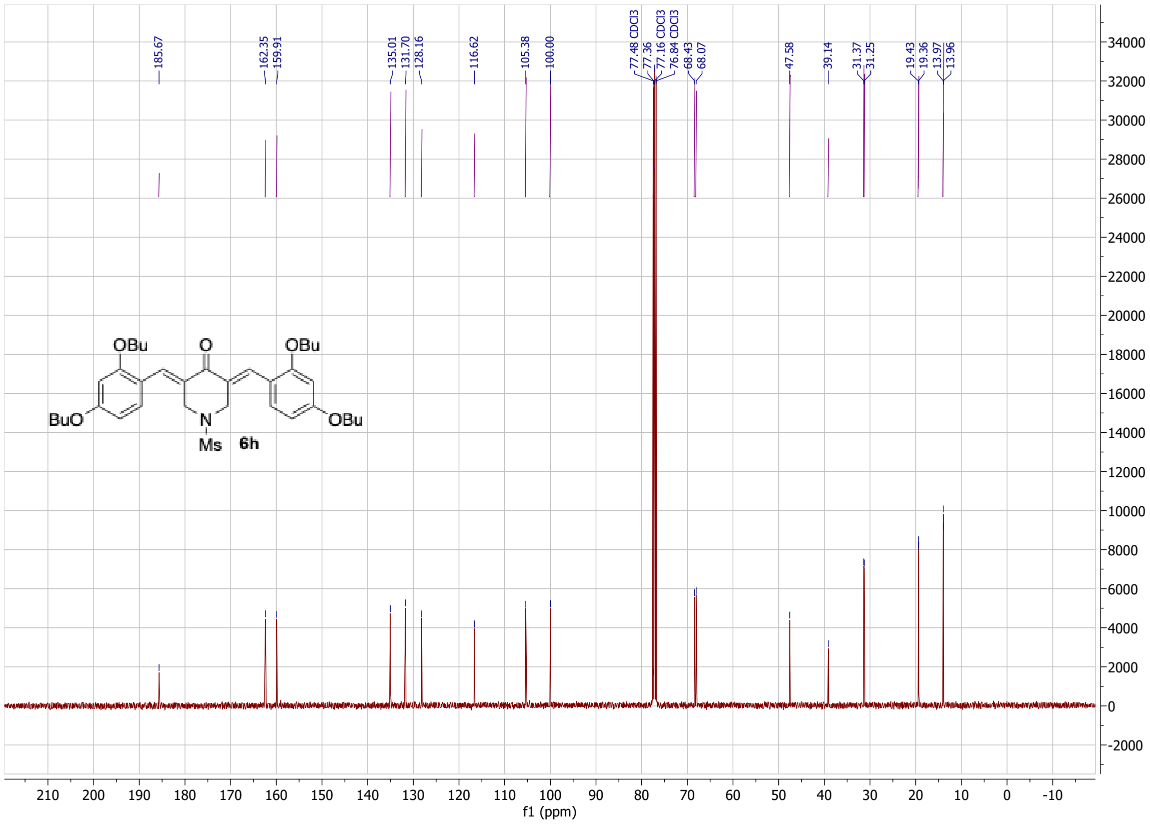

^{1}H NMR for 7h

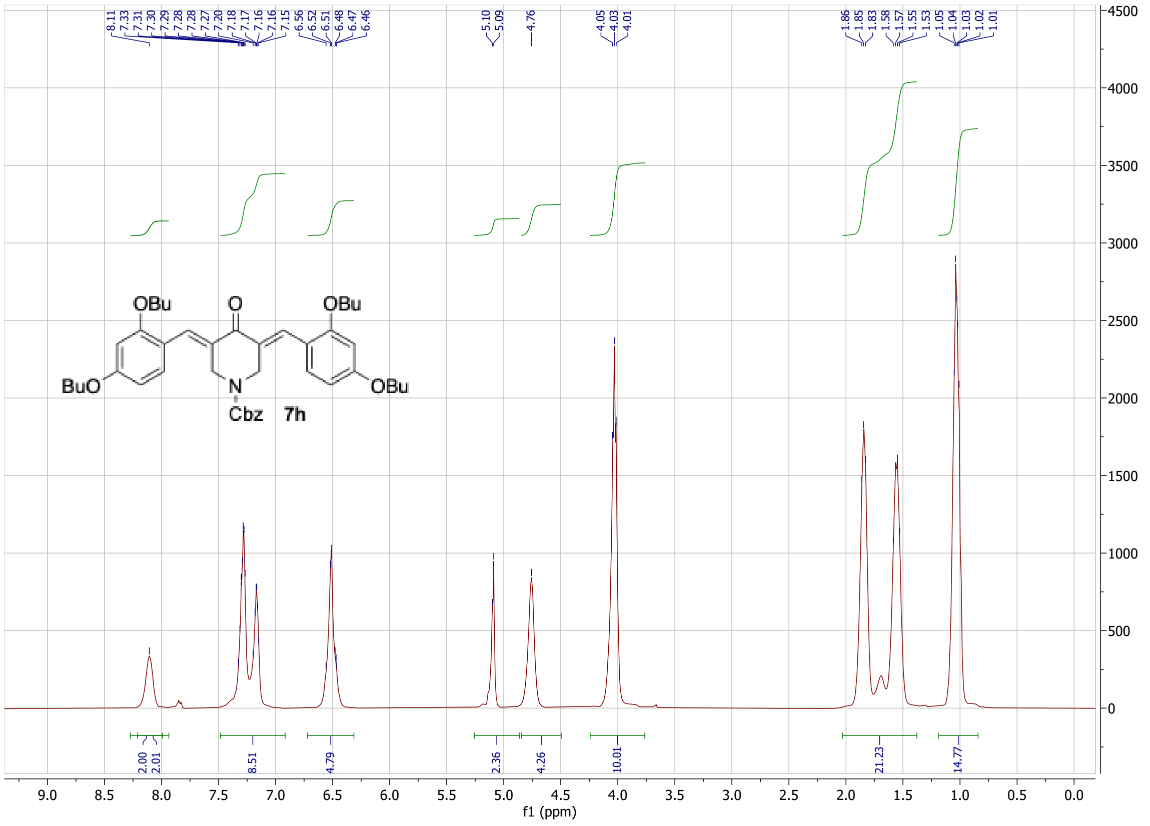

^{13}C NMR for 7h

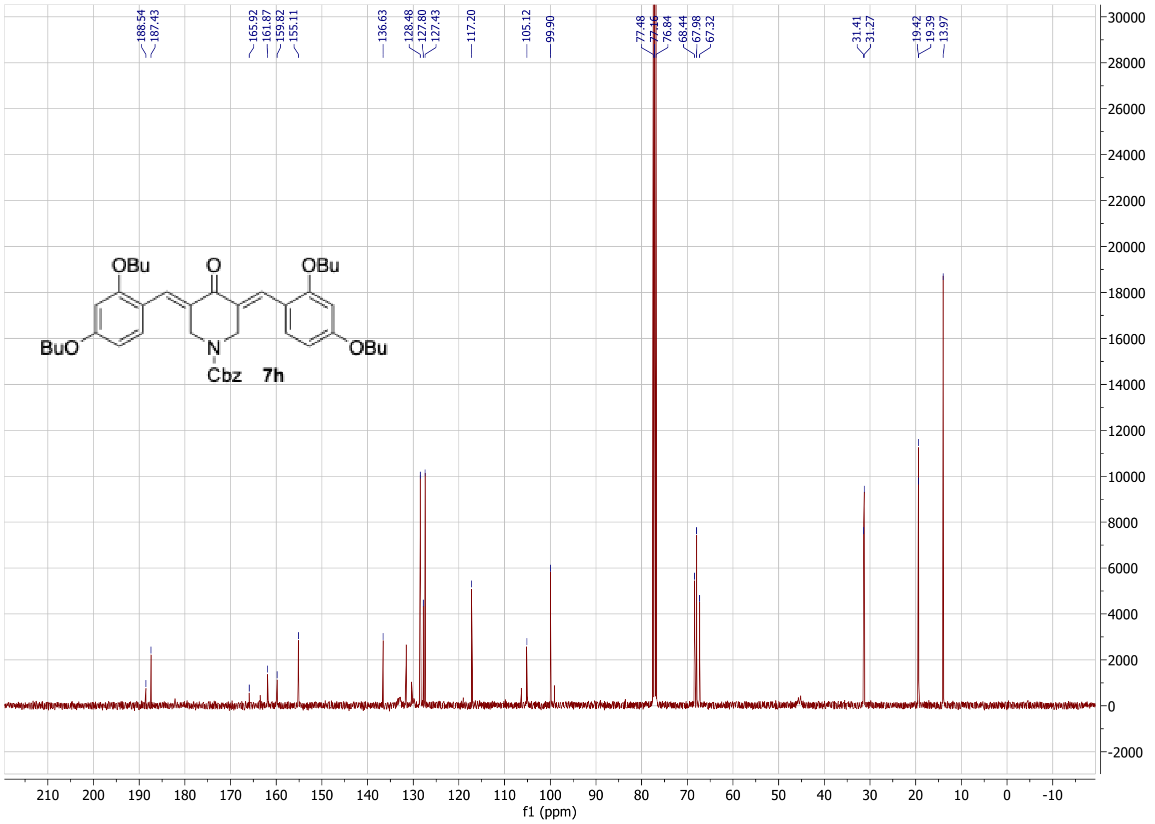

^{1}H NMR for 8h

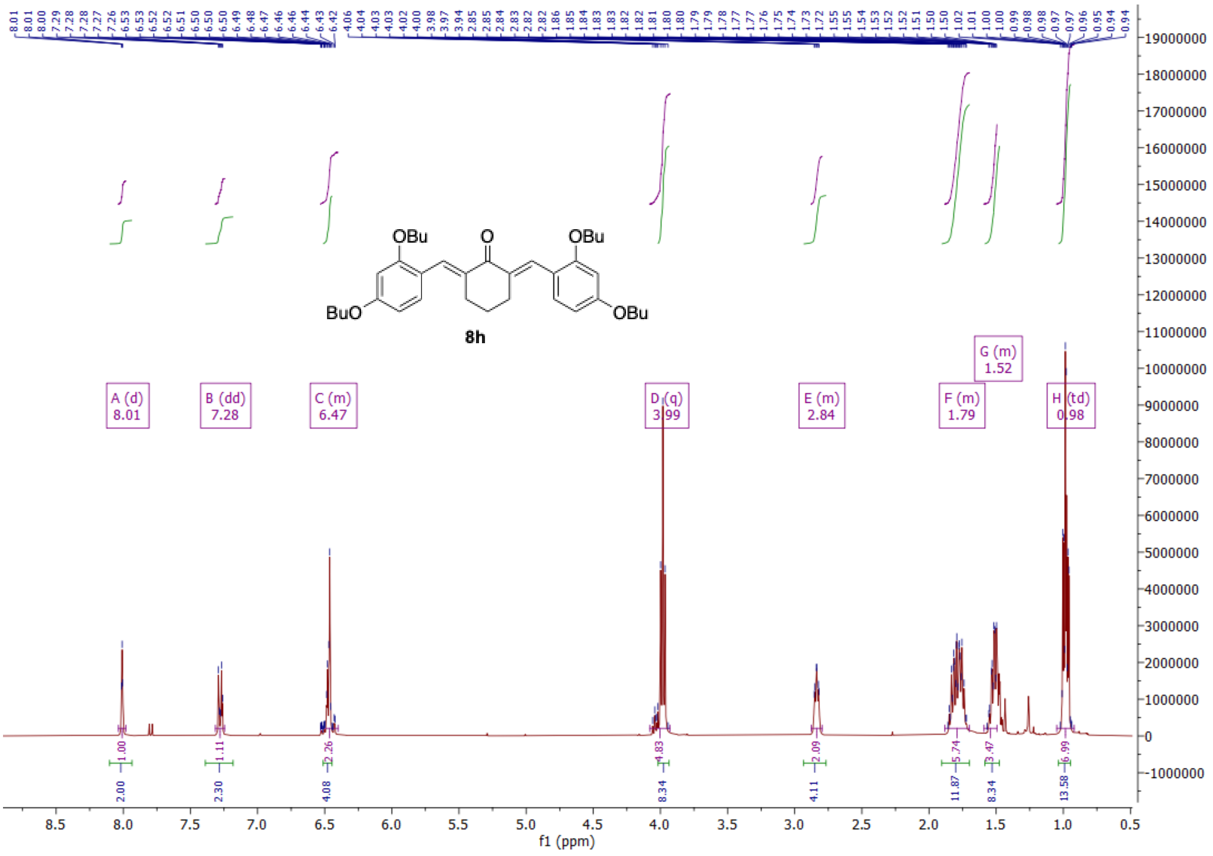

^{13}C NMR for 8h

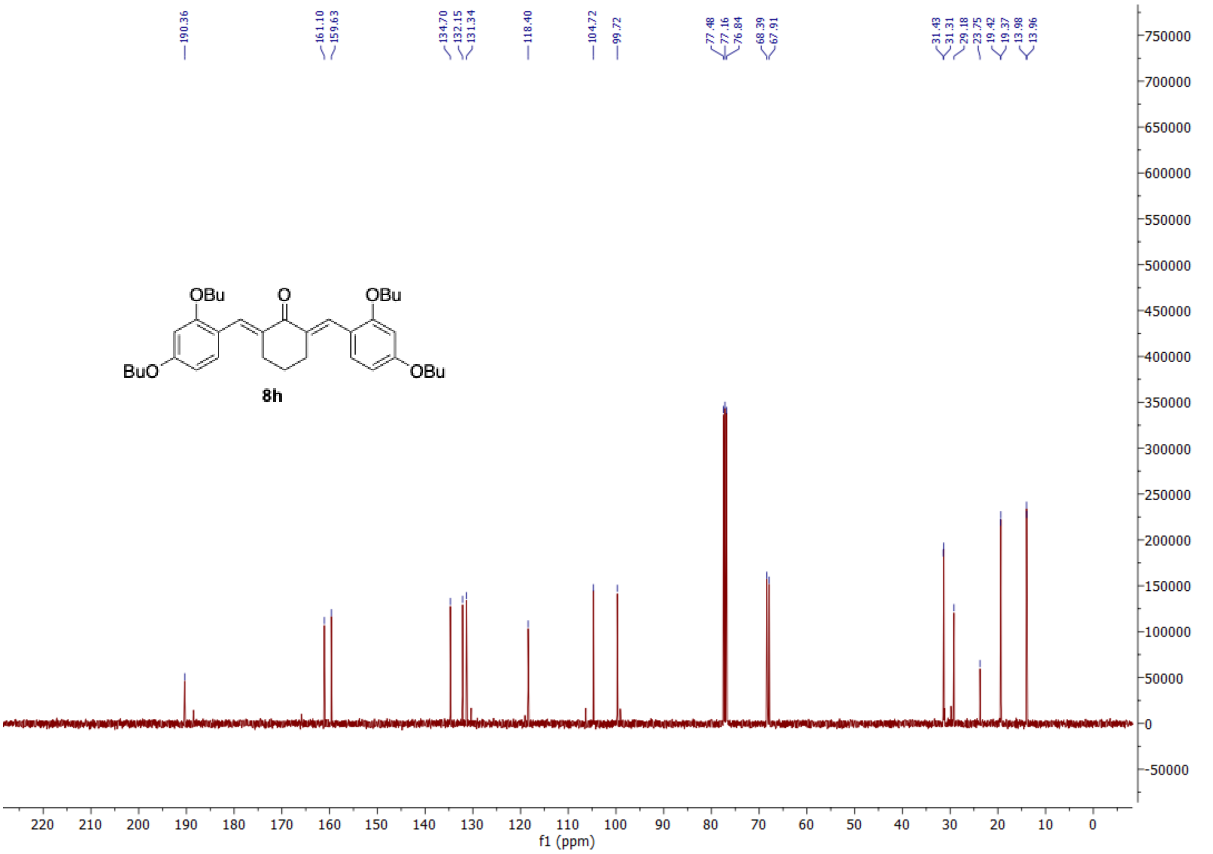

^{1}H NMR for 9h

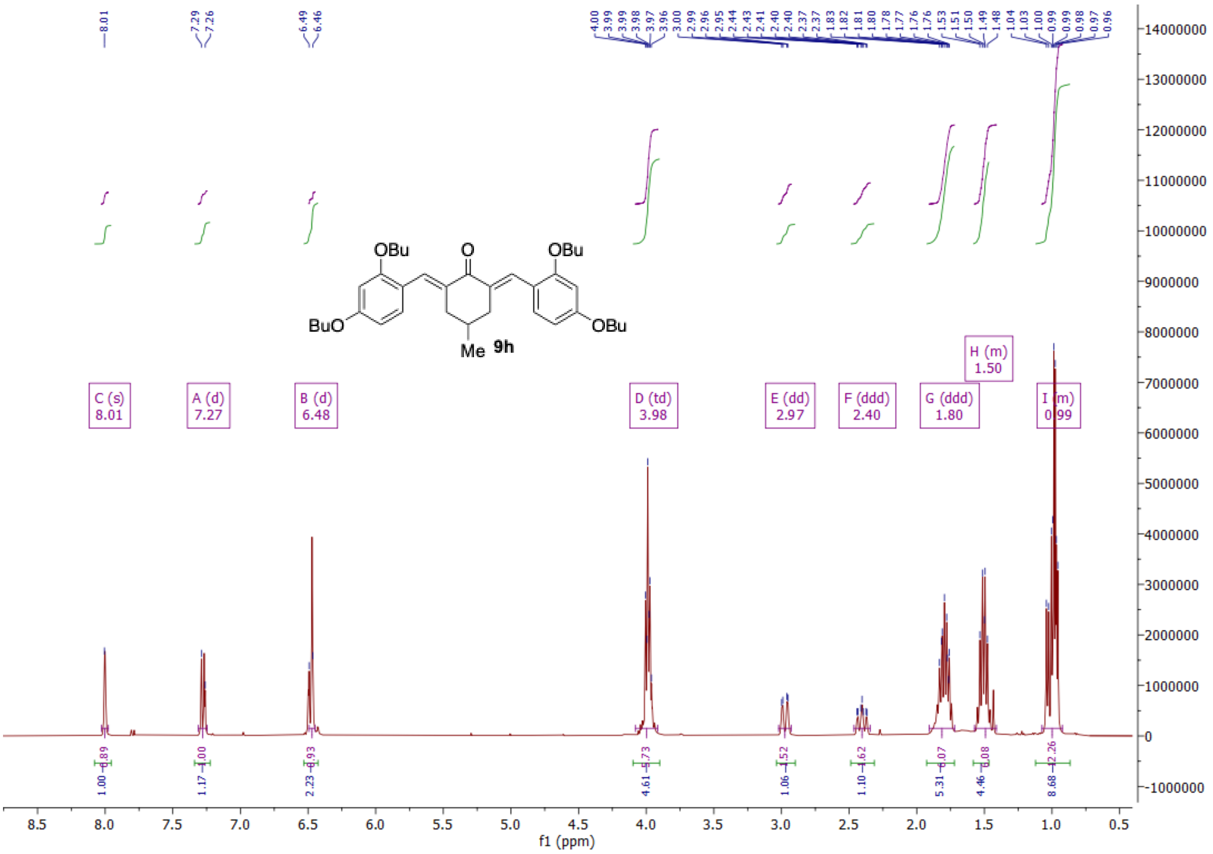

^{13}C NMR for 9h

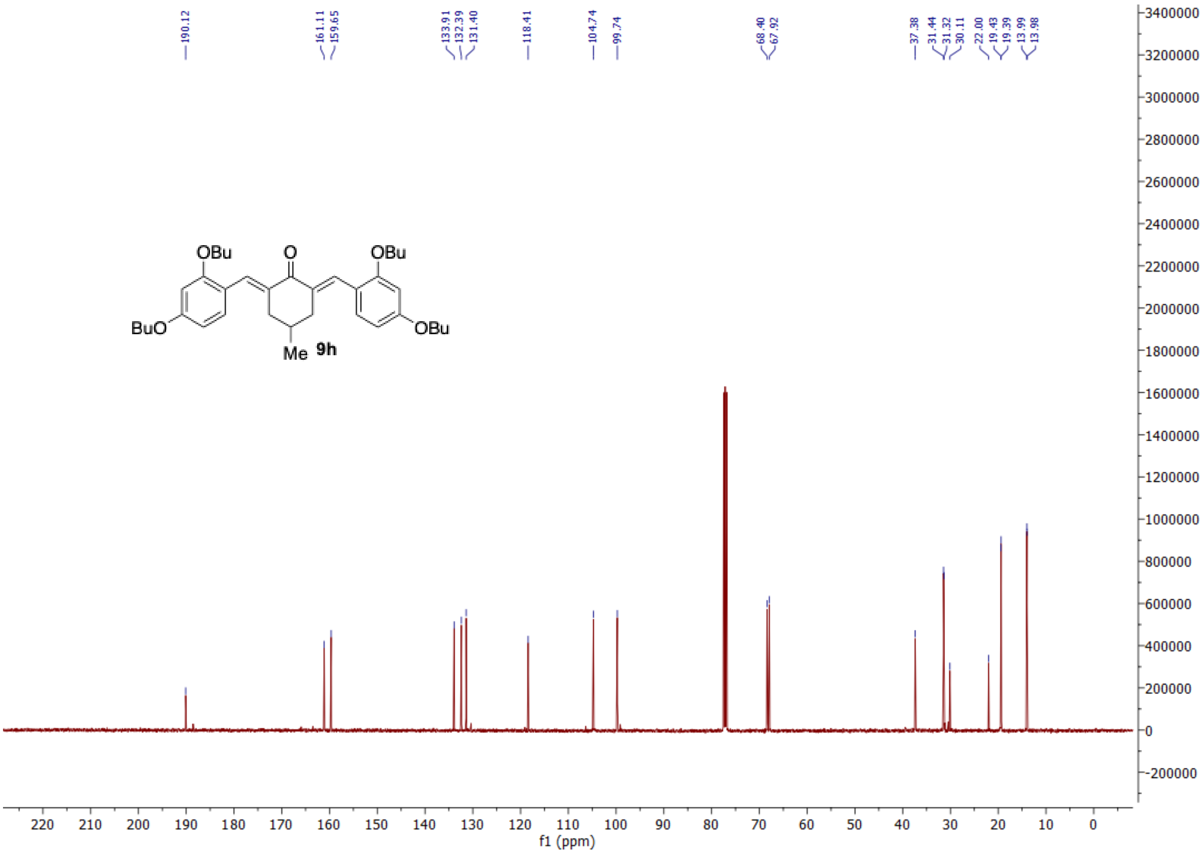

^{1}H NMR for 10h

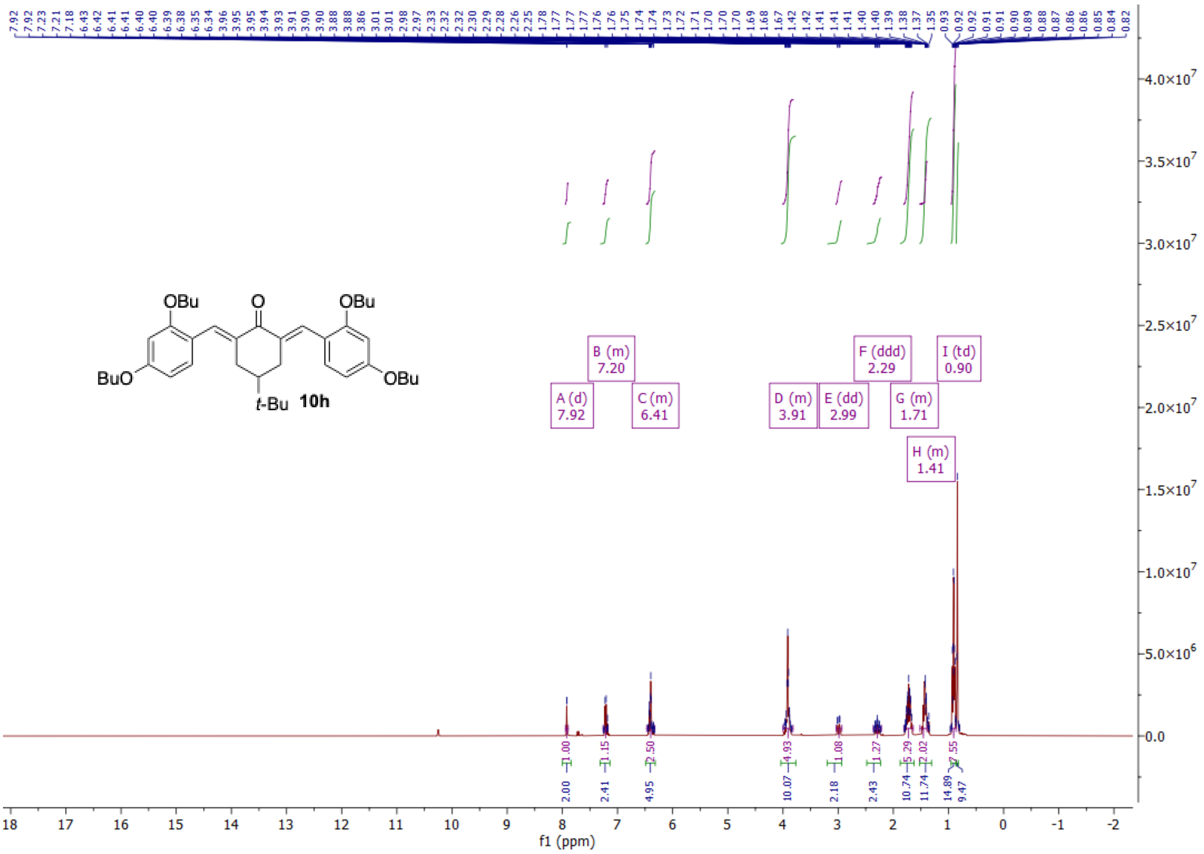

^{13}C NMR for 10h

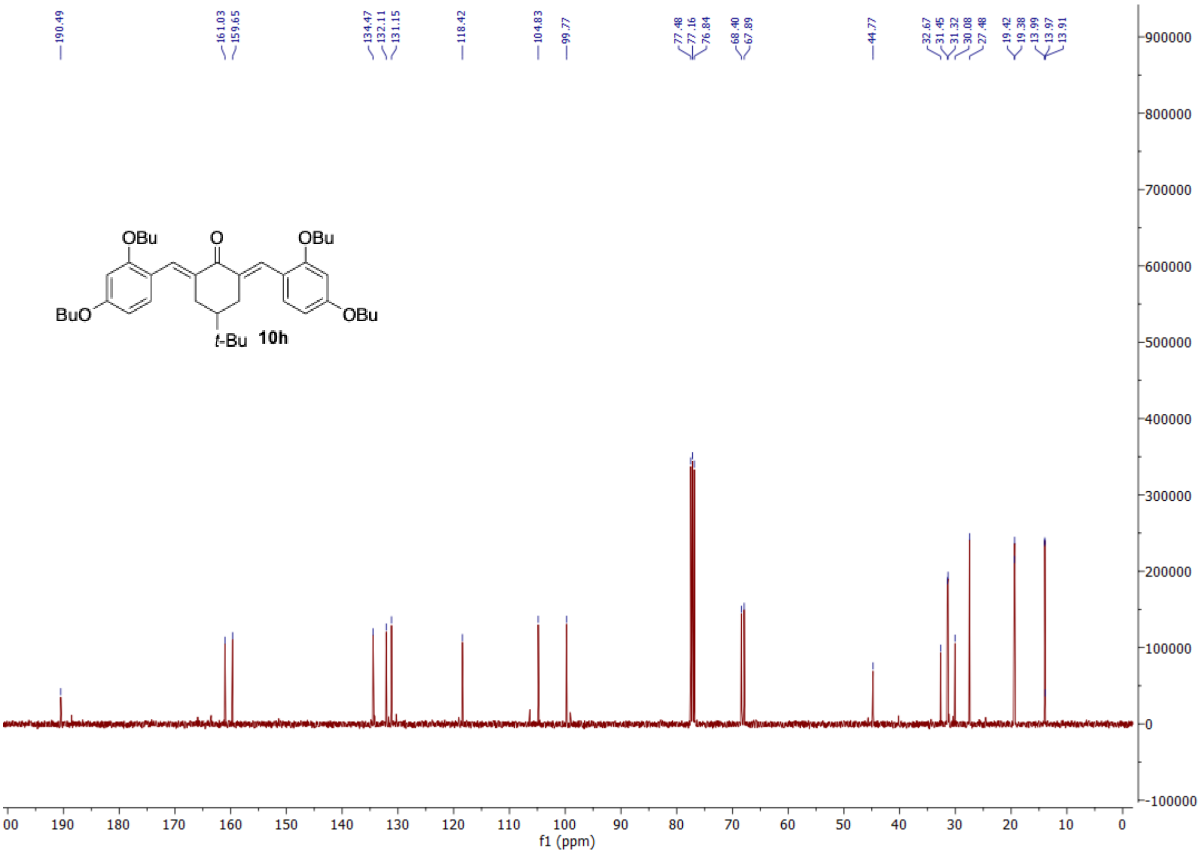

3- Molecular Docking

All the ligands were fully optimized with the Gaussian16 Rev. A03 package at the HF/6-31G(d) level of theory. The atomic charges were computed at the HF/6-31G(d) level of theory with the RESP scheme.
For docking studies, X-ray crystal structure of sterol 14alpha-demethylase (CYP51) from Trypanosoma cruzi in complex with inhibitor fluconazole was obtained from the Protein Data Bank (PDB). (PDB code: 3KHM). The enzyme was prepared by removing fluconazole, then adding hydrogen atoms and atomic charges with AutoDock Tools software. The molecular docking studies were carried out with the AutoDock 4.2.6 software. The docking box set at 70 × 70 × 70 Å and was centered at x = 2.270 Å, y = -23.537 Å and z = 16.924 Å (center of fluconazole). The Lamarckian Genetic Algorithm with default parameters was applied for simulation. The number of docking runs was 20.
The pose drawings and interaction calculations were performed with Protein*Plus*. The 3D active sites were drawn with PyMOL.

Cartesian coordinates (mol2 format) of ligands at HF/6-31G(d) with RESP charges.

Fluconazole

@<TRIPOS>MOLECULE
MOL
34 36 1 0 0
SMALL
resp

```
@<TRIPOS>ATOM
      1 O1          -0.7120   -1.8010    1.3380 oh         1 MOL      -0.634651
      2 C1          -0.3330   -0.7100    0.5450 c3         1 MOL       0.283043
      3 C2          -1.1660   -0.8440   -0.7470 c3         1 MOL      -0.083044
      4 C3          -0.6730    0.5480    1.3810 c3         1 MOL      -0.083044
      5 C4           1.1700   -0.8030    0.2130 ca         1 MOL      -0.006406
      6 N1          -2.5920   -0.9390   -0.5350 na         1 MOL       0.137152
      7 C5          -3.5210    0.0200   -0.5550 cc         1 MOL       0.315372
      8 N2          -3.1960   -2.1280   -0.3620 nc         1 MOL      -0.575578
      9 N3          -4.7060   -0.4840   -0.3830 nd         1 MOL      -0.676425
     10 C6          -4.4450   -1.8080   -0.2720 cd         1 MOL       0.468276
     11 N4          -0.3580    1.8330    0.8120 na         1 MOL       0.137152
     12 C7           0.6590    2.6510    1.0750 cc         1 MOL       0.315372
     13 N5          -1.1490    2.4100   -0.1090 nc         1 MOL      -0.575578
     14 N6           0.5780    3.7360    0.3680 nd         1 MOL      -0.676425
     15 C8          -0.5510    3.5330   -0.3450 cd         1 MOL       0.468276
     16 C9           1.9740   -1.7370    0.8640 ca         1 MOL      -0.161910
     17 C10          1.8120   -0.0120   -0.7300 ca         1 MOL       0.015638
     18 C11          3.3310   -1.8550    0.6190 ca         1 MOL      -0.225620
     19 C12          3.9030   -1.0220   -0.3160 ca         1 MOL       0.240785
     20 F1           5.1990   -1.1170   -0.5670 f          1 MOL      -0.171388
     21 C13          3.1600   -0.0930   -1.0080 ca         1 MOL      -0.157098
     22 F2           1.1200    0.8810   -1.4320 f          1 MOL      -0.074687
     23 H1          -0.4960   -1.6590    2.2500 ho         1 MOL       0.440634
     24 H2          -0.8630   -1.7550   -1.2410 h1         1 MOL       0.091368
     25 H3          -0.9760   -0.0100   -1.4000 h1         1 MOL       0.091368
     26 H4          -0.1270    0.4780    2.3150 h1         1 MOL       0.091368
     27 H5          -1.7280    0.5290    1.6110 h1         1 MOL       0.091368
     28 H6          -3.2800    1.0530   -0.6900 h5         1 MOL       0.114828
     29 H7          -5.2100   -2.5420   -0.1280 h5         1 MOL       0.088428
     30 H8          -0.9340    4.2440   -1.0470 h5         1 MOL       0.088428
     31 H9           1.5240   -2.4110    1.5640 ha         1 MOL       0.164546
     32 H10          3.9320   -2.5820    1.1300 ha         1 MOL       0.175042
     33 H11          3.6040    0.5470   -1.7450 ha         1 MOL       0.168580
     34 H12          1.4250    2.4180    1.7860 h5         1 MOL       0.114828
@<TRIPOS>BOND
     1    1    2 1
     2    1   23 1
     3    2    3 1
     4    2    4 1
     5    2    5 1
     6    3    6 1
     7    3   24 1
     8    3   25 1
     9    4   11 1
    10    4   26 1
    11    4   27 1
    12    5   16 ar
    13    5   17 ar
    14    6    7 1
    15    6    8 1
```

```
    16     7     9 2
    17     7    28 1
    18     8    10 2
    19     9    10 1
    20    10    29 1
    21    11    12 1
    22    11    13 1
    23    12    14 2
    24    12    34 1
    25    13    15 2
    26    14    15 1
    27    15    30 1
    28    16    18 ar
    29    16    31 1
    30    17    21 ar
    31    17    22 1
    32    18    19 ar
    33    18    32 1
    34    19    20 1
    35    19    21 ar
    36    21    33 1
@<TRIPOS>SUBSTRUCTURE
     1 MOL         1 TEMP              0 ****  ****    0 ROOT
```

2h

```
@<TRIPOS>MOLECULE
MOL
   96    98     1     0     0
SMALL
resp


@<TRIPOS>ATOM
      1 C1          1.2450    -0.8490     0.6090 c3         1 MOL      -0.215201
      2 C2         -1.1270    -0.7420     0.7150 c3         1 MOL      -0.215201
      3 C3         -0.0190    -0.1810    -1.5480 c          1 MOL       0.494949
      4 H1         -2.0100    -1.2480     1.0780 h1         1 MOL       0.156916
      5 H2          1.2840     0.0750     1.1820 h1         1 MOL       0.156916
      6 H3          2.1150    -1.4160     0.8980 h1         1 MOL       0.156916
      7 H4         -1.0420     0.1790     1.2830 h1         1 MOL       0.156916
      8 N1          0.0420    -1.5510     1.0120 n3         1 MOL      -0.425862
      9 O1         -0.0570     0.2210    -2.6780 o          1 MOL      -0.534487
     10 C4         -1.2760    -0.4200    -0.7590 ce         1 MOL      -0.028397
     11 C5          1.2900    -0.5020    -0.8740 ce         1 MOL      -0.028397
     12 C6         -2.4220    -0.3860    -1.4300 cf         1 MOL      -0.115908
     13 H5         -2.3520    -0.2010    -2.4880 ha         1 MOL       0.137959
     14 C7          2.3860    -0.4060    -1.6210 cf         1 MOL      -0.115908
     15 H6          2.2550    -0.0520    -2.6270 ha         1 MOL       0.137959
     16 C8         -3.7940    -0.6340    -0.9220 ca         1 MOL       0.052252
     17 C9         -4.3640     0.0570     0.1560 ca         1 MOL       0.158182
```

18	C10	-4.5820	-1.5680	-1.5840	ca	1 MOL	-0.235103
19	C11	-5.6650	-0.2210	0.5540	ca	1 MOL	-0.169786
20	C12	-5.8750	-1.8640	-1.1900	ca	1 MOL	-0.155068
21	H7	-4.1590	-2.0880	-2.4250	ha	1 MOL	0.166558
22	C13	-6.4090	-1.1810	-0.1130	ca	1 MOL	0.125725
23	H8	-6.1220	0.2830	1.3810	ha	1 MOL	0.162019
24	H9	-6.4530	-2.6120	-1.6990	ha	1 MOL	0.162292
25	C14	3.7860	-0.7060	-1.2280	ca	1 MOL	0.052252
26	C15	4.1760	-1.9480	-0.7350	ca	1 MOL	-0.235103
27	C16	4.7810	0.2470	-1.4450	ca	1 MOL	0.158182
28	C17	5.4970	-2.2270	-0.4330	ca	1 MOL	-0.155068
29	H10	3.4360	-2.7180	-0.6140	ha	1 MOL	0.166558
30	C18	6.1050	-0.0180	-1.1430	ca	1 MOL	-0.169786
31	C19	6.4620	-1.2550	-0.6330	ca	1 MOL	0.125725
32	H11	5.7880	-3.1960	-0.0710	ha	1 MOL	0.162292
33	H12	6.8570	0.7250	-1.3350	ha	1 MOL	0.162019
34	O2	-7.6640	-1.4490	0.3330	os	1 MOL	-0.349571
35	O3	-3.6030	0.9950	0.7500	os	1 MOL	-0.315353
36	O4	4.4520	1.4450	-1.9950	os	1 MOL	-0.315353
37	O5	7.7690	-1.5250	-0.3720	os	1 MOL	-0.349571
38	C20	-0.0460	-2.9300	0.5510	c3	1 MOL	0.166601
39	H13	-1.0800	-3.2400	0.6450	h1	1 MOL	0.028075
40	C21	-8.7290	-0.9180	-0.4300	c3	1 MOL	0.063005
41	C22	-10.0320	-1.3220	0.2340	c3	1 MOL	0.034364
42	H14	-8.6800	-1.2970	-1.4460	h1	1 MOL	0.042408
43	H15	-8.6350	0.1640	-0.4750	h1	1 MOL	0.042408
44	C23	-11.2590	-0.7950	-0.5110	c3	1 MOL	0.036484
45	H16	-10.0680	-2.4060	0.2930	hc	1 MOL	0.020292
46	H17	-10.0290	-0.9530	1.2560	hc	1 MOL	0.020292
47	C24	-12.5730	-1.2020	0.1560	c3	1 MOL	-0.086697
48	H18	-11.2090	0.2900	-0.5750	hc	1 MOL	-0.000488
49	H19	-11.2490	-1.1620	-1.5360	hc	1 MOL	-0.000488
50	H20	-13.4260	-0.8160	-0.3920	hc	1 MOL	0.018656
51	H21	-12.6700	-2.2820	0.2040	hc	1 MOL	0.018656
52	H22	-12.6300	-0.8200	1.1710	hc	1 MOL	0.018656
53	C25	-4.1450	1.8360	1.7400	c3	1 MOL	0.043417
54	C26	-3.0820	2.8510	2.1220	c3	1 MOL	0.029588
55	H23	-4.4400	1.2490	2.6050	h1	1 MOL	0.043839
56	H24	-5.0280	2.3350	1.3540	h1	1 MOL	0.043839
57	C27	-3.5760	3.8410	3.1780	c3	1 MOL	0.053695
58	H25	-2.2090	2.3210	2.4910	hc	1 MOL	0.016908
59	H26	-2.7740	3.3820	1.2260	hc	1 MOL	0.016908
60	C28	-2.5090	4.8620	3.5710	c3	1 MOL	-0.075089
61	H27	-4.4540	4.3650	2.8040	hc	1 MOL	-0.004835
62	H28	-3.8980	3.2990	4.0650	hc	1 MOL	-0.004835
63	H29	-2.8850	5.5530	4.3190	hc	1 MOL	0.015257
64	H30	-1.6320	4.3720	3.9830	hc	1 MOL	0.015257
65	H31	-2.1900	5.4450	2.7120	hc	1 MOL	0.015257
66	C29	4.1560	2.4940	-1.0940	c3	1 MOL	0.043417
67	C30	3.7480	3.7100	-1.9050	c3	1 MOL	0.029588
68	H32	5.0320	2.7070	-0.4870	h1	1 MOL	0.043839

```
  69 H33       3.3550   2.1870  -0.4280 h1     1 MOL     0.043839
  70 C31       3.4110   4.9150  -1.0260 c3     1 MOL     0.053695
  71 H34       4.5570   3.9590  -2.5860 hc     1 MOL     0.016908
  72 H35       2.8920   3.4430  -2.5170 hc     1 MOL     0.016908
  73 C32       2.9970   6.1400  -1.8410 c3     1 MOL    -0.075089
  74 H36       2.6070   4.6520  -0.3410 hc     1 MOL    -0.004835
  75 H37       4.2700   5.1680  -0.4080 hc     1 MOL    -0.004835
  76 H38       2.7630   6.9800  -1.1950 hc     1 MOL     0.015257
  77 H39       3.7920   6.4490  -2.5120 hc     1 MOL     0.015257
  78 H40       2.1190   5.9290  -2.4440 hc     1 MOL     0.015257
  79 C33       8.2080  -1.2490   0.9420 c3     1 MOL     0.063005
  80 C34       9.6770  -1.6160   1.0360 c3     1 MOL     0.034364
  81 H41       7.6190  -1.8240   1.6520 h1     1 MOL     0.042408
  82 H42       8.0560  -0.1960   1.1630 h1     1 MOL     0.042408
  83 C35      10.2600  -1.3550   2.4260 c3     1 MOL     0.036484
  84 H43       9.7890  -2.6650   0.7750 hc     1 MOL     0.020292
  85 H44      10.2240  -1.0480   0.2890 hc     1 MOL     0.020292
  86 C36      11.7400  -1.7250   2.5230 c3     1 MOL    -0.086697
  87 H45      10.1350  -0.3060   2.6830 hc     1 MOL    -0.000488
  88 H46       9.7000  -1.9210   3.1680 hc     1 MOL    -0.000488
  89 H47      12.1280  -1.5300   3.5170 hc     1 MOL     0.018656
  90 H48      11.8950  -2.7770   2.3060 hc     1 MOL     0.018656
  91 H49      12.3330  -1.1510   1.8170 hc     1 MOL     0.018656
  92 H50       0.2110  -3.0340  -0.5050 h1     1 MOL     0.028075
  93 C37       0.8170  -3.8720   1.3860 c3     1 MOL    -0.074814
  94 H51       1.8730  -3.6370   1.3100 hc     1 MOL     0.018363
  95 H52       0.5360  -3.8100   2.4310 hc     1 MOL     0.018363
  96 H53       0.6860  -4.8970   1.0510 hc     1 MOL     0.018363
@<TRIPOS>BOND
     1     1     5 1
     2     1     6 1
     3     1     8 1
     4     1    11 1
     5     2     4 1
     6     2     7 1
     7     2     8 1
     8     2    10 1
     9     3     9 2
    10     3    10 1
    11     3    11 1
    12     8    38 1
    13    10    12 2
    14    11    14 2
    15    12    13 1
    16    12    16 1
    17    14    15 1
    18    14    25 1
    19    16    17 ar
    20    16    18 ar
    21    17    19 ar
    22    17    35 1
```

23	18	20 ar
24	18	21 1
25	19	22 ar
26	19	23 1
27	20	22 ar
28	20	24 1
29	22	34 1
30	25	26 ar
31	25	27 ar
32	26	28 ar
33	26	29 1
34	27	30 ar
35	27	36 1
36	28	31 ar
37	28	32 1
38	30	31 ar
39	30	33 1
40	31	37 1
41	34	40 1
42	35	53 1
43	36	66 1
44	37	79 1
45	38	39 1
46	38	92 1
47	38	93 1
48	40	41 1
49	40	42 1
50	40	43 1
51	41	44 1
52	41	45 1
53	41	46 1
54	44	47 1
55	44	48 1
56	44	49 1
57	47	50 1
58	47	51 1
59	47	52 1
60	53	54 1
61	53	55 1
62	53	56 1
63	54	57 1
64	54	58 1
65	54	59 1
66	57	60 1
67	57	61 1
68	57	62 1
69	60	63 1
70	60	64 1
71	60	65 1
72	66	67 1
73	66	68 1

```
   74    66    69 1
   75    67    70 1
   76    67    71 1
   77    67    72 1
   78    70    73 1
   79    70    74 1
   80    70    75 1
   81    73    76 1
   82    73    77 1
   83    73    78 1
   84    79    80 1
   85    79    81 1
   86    79    82 1
   87    80    83 1
   88    80    84 1
   89    80    85 1
   90    83    86 1
   91    83    87 1
   92    83    88 1
   93    86    89 1
   94    86    90 1
   95    86    91 1
   96    93    94 1
   97    93    95 1
   98    93    96 1
@<TRIPOS>SUBSTRUCTURE
     1 MOL         1 TEMP              0 ****  ****     0 ROOT
```

3h

```
@<TRIPOS>MOLECULE
MOL
  103   106     1     0     0
SMALL
resp


@<TRIPOS>ATOM
      1 C1          -1.3820    -0.7520    -0.9200 c3         1 MOL      -0.100381
      2 C2           0.9910    -0.4330    -1.0050 c3         1 MOL      -0.100381
      3 C3          -0.0970    -0.6100     1.3320 c          1 MOL       0.494561
      4 H1           1.8960    -0.7760    -1.4900 h1         1 MOL       0.109431
      5 H2          -1.5660     0.2600    -1.2790 h1         1 MOL       0.109431
      6 H3          -2.1840    -1.3580    -1.3140 h1         1 MOL       0.109431
      7 H4           0.8510     0.6010    -1.3090 h1         1 MOL       0.109431
      8 N1          -0.1360    -1.1920    -1.5010 n3         1 MOL      -0.368327
      9 O1          -0.0530    -0.6010     2.5300 o          1 MOL      -0.538735
     10 C4           1.1510    -0.4940     0.5020 ce         1 MOL      -0.027500
     11 C5          -1.4120    -0.7210     0.6030 ce         1 MOL      -0.027500
     12 C6           2.3070    -0.4810     1.1540 cf         1 MOL      -0.114338
```

13 H5	2.2550	-0.5830	2.2230 ha	1 MOL	0.134932
14 C7	-2.5110	-0.6790	1.3520 cf	1 MOL	-0.114338
15 H6	-2.3680	-0.5550	2.4100 ha	1 MOL	0.134932
16 C8	3.6780	-0.4210	0.5890 ca	1 MOL	0.069321
17 C9	4.1280	0.5940	-0.2660 ca	1 MOL	0.171990
18 C10	4.5870	-1.4020	0.9680 ca	1 MOL	-0.260277
19 C11	5.4360	0.5840	-0.7340 ca	1 MOL	-0.162028
20 C12	5.8890	-1.4290	0.5000 ca	1 MOL	-0.167379
21 H7	4.2530	-2.1750	1.6370 ha	1 MOL	0.180178
22 C13	6.3050	-0.4270	-0.3560 ca	1 MOL	0.137902
23 H8	5.8030	1.3400	-1.3980 ha	1 MOL	0.158437
24 H9	6.5660	-2.2140	0.7830 ha	1 MOL	0.164194
25 C14	-3.9260	-0.7370	0.9060 ca	1 MOL	0.069321
26 C15	-4.4340	-1.7630	0.1140 ca	1 MOL	-0.260277
27 C16	-4.8250	0.2240	1.3700 ca	1 MOL	0.171990
28 C17	-5.7710	-1.8200	-0.2360 ca	1 MOL	-0.167379
29 H10	-3.7780	-2.5480	-0.2130 ha	1 MOL	0.180178
30 C18	-6.1650	0.1810	1.0250 ca	1 MOL	-0.162028
31 C19	-6.6370	-0.8400	0.2190 ca	1 MOL	0.137902
32 H11	-6.1520	-2.6290	-0.8320 ha	1 MOL	0.164194
33 H12	-6.8400	0.9230	1.4100 ha	1 MOL	0.158437
34 O2	7.5660	-0.4210	-0.8650 os	1 MOL	-0.363411
35 O3	3.2470	1.5620	-0.5800 os	1 MOL	-0.344534
36 O4	-4.3900	1.2030	2.2060 os	1 MOL	-0.344534
37 O5	-7.9600	-0.9010	-0.0890 os	1 MOL	-0.363411
38 C20	0.0600	-2.6040	-1.7780 c3	1 MOL	-0.138586
39 H13	0.8170	-2.6690	-2.5530 h1	1 MOL	0.109232
40 H14	-0.8610	-2.9670	-2.2220 h1	1 MOL	0.109232
41 C21	0.4680	-3.5450	-0.6490 ca	1 MOL	0.052759
42 C22	-0.4670	-4.0610	0.2420 ca	1 MOL	-0.098055
43 C23	1.7930	-3.9410	-0.5120 ca	1 MOL	-0.098055
44 C24	-0.0880	-4.9270	1.2540 ca	1 MOL	-0.185978
45 H15	-1.5030	-3.7870	0.1480 ha	1 MOL	0.118521
46 C25	2.1790	-4.8110	0.4950 ca	1 MOL	-0.185978
47 H16	2.5340	-3.5660	-1.1970 ha	1 MOL	0.118521
48 C26	1.2390	-5.3050	1.3840 ca	1 MOL	-0.090820
49 H17	-0.8270	-5.3090	1.9350 ha	1 MOL	0.139311
50 H18	3.2100	-5.1060	0.5810 ha	1 MOL	0.139311
51 H19	1.5350	-5.9800	2.1670 ha	1 MOL	0.125142
52 C27	8.5850	0.0450	-0.0040 c3	1 MOL	0.090170
53 C28	9.9030	-0.0250	-0.7540 c3	1 MOL	0.035844
54 H20	8.6190	-0.5670	0.8930 h1	1 MOL	0.034460
55 H21	8.3640	1.0650	0.2990 h1	1 MOL	0.034460
56 C29	11.0840	0.4610	0.0890 c3	1 MOL	0.041632
57 H22	10.0670	-1.0510	-1.0700 hc	1 MOL	0.017241
58 H23	9.8190	0.5710	-1.6580 hc	1 MOL	0.017241
59 C30	12.4120	0.3910	-0.6650 c3	1 MOL	-0.075494
60 H24	10.9060	1.4850	0.4090 hc	1 MOL	-0.004373
61 H25	11.1540	-0.1360	0.9960 hc	1 MOL	-0.004373
62 H26	13.2320	0.7410	-0.0460 hc	1 MOL	0.015337
63 H27	12.6370	-0.6270	-0.9690 hc	1 MOL	0.015337

```
  64 H28        12.3870    1.0060   -1.5600 hc       1 MOL      0.015337
  65 C31         3.6570    2.6960   -1.3050 c3       1 MOL      0.071892
  66 C32         2.4770    3.6480   -1.3870 c3       1 MOL      0.036353
  67 H29         3.9810    2.4030   -2.3000 h1       1 MOL      0.035014
  68 H30         4.4950    3.1700   -0.8030 h1       1 MOL      0.035014
  69 C33         2.8190    4.9360   -2.1370 c3       1 MOL      0.051427
  70 H31         1.6520    3.1410   -1.8800 hc       1 MOL      0.015947
  71 H32         2.1480    3.8780   -0.3780 hc       1 MOL      0.015947
  72 C34         1.6340    5.8960   -2.2280 c3       1 MOL     -0.069928
  73 H33         3.6490    5.4360   -1.6420 hc       1 MOL     -0.005854
  74 H34         3.1630    4.6940   -3.1410 hc       1 MOL     -0.005854
  75 H35         1.9030    6.8020   -2.7620 hc       1 MOL      0.013410
  76 H36         0.7990    5.4390   -2.7500 hc       1 MOL      0.013410
  77 H37         1.2880    6.1850   -1.2390 hc       1 MOL      0.013410
  78 C35        -3.9590    2.4060    1.5990 c3       1 MOL      0.071892
  79 C36        -3.4500    3.3280    2.6920 c3       1 MOL      0.036353
  80 H38        -4.7900    2.8580    1.0630 h1       1 MOL      0.035014
  81 H39        -3.1750    2.1910    0.8790 h1       1 MOL      0.035014
  82 C37        -2.9620    4.6710    2.1460 c3       1 MOL      0.051427
  83 H40        -4.2490    3.4850    3.4120 hc       1 MOL      0.015947
  84 H41        -2.6460    2.8240    3.2190 hc       1 MOL      0.015947
  85 C38        -2.4470    5.5990    3.2450 c3       1 MOL     -0.069928
  86 H42        -2.1710    4.5010    1.4190 hc       1 MOL     -0.005854
  87 H43        -3.7710    5.1630    1.6090 hc       1 MOL     -0.005854
  88 H44        -2.1070    6.5440    2.8320 hc       1 MOL      0.013410
  89 H45        -3.2250    5.8160    3.9710 hc       1 MOL      0.013410
  90 H46        -1.6140    5.1500    3.7770 hc       1 MOL      0.013410
  91 C39        -8.3510   -0.2400   -1.2750 c3       1 MOL      0.090170
  92 C40        -9.8480   -0.4220   -1.4460 c3       1 MOL      0.035844
  93 H47        -7.8110   -0.6570   -2.1210 h1       1 MOL      0.034460
  94 H48        -8.0940    0.8140   -1.2060 h1       1 MOL      0.034460
  95 C41       -10.3830    0.2590   -2.7070 c3       1 MOL      0.041632
  96 H49       -10.0660   -1.4860   -1.4750 hc       1 MOL      0.017241
  97 H50       -10.3470   -0.0250   -0.5660 hc       1 MOL      0.017241
  98 C42       -11.8900    0.0770   -2.8800 c3       1 MOL     -0.075494
  99 H51       -10.1510    1.3220   -2.6730 hc       1 MOL     -0.004373
 100 H52        -9.8700   -0.1380   -3.5800 hc       1 MOL     -0.004373
 101 H53       -12.2430    0.5690   -3.7810 hc       1 MOL      0.015337
 102 H54       -12.1510   -0.9750   -2.9520 hc       1 MOL      0.015337
 103 H55       -12.4340    0.4940   -2.0380 hc       1 MOL      0.015337
@<TRIPOS>BOND
     1     1     5 1
     2     1     6 1
     3     1     8 1
     4     1    11 1
     5     2     4 1
     6     2     7 1
     7     2     8 1
     8     2    10 1
     9     3     9 2
    10     3    10 1
```

11	3	11 1
12	8	38 1
13	10	12 2
14	11	14 2
15	12	13 1
16	12	16 1
17	14	15 1
18	14	25 1
19	16	17 ar
20	16	18 ar
21	17	19 ar
22	17	35 1
23	18	20 ar
24	18	21 1
25	19	22 ar
26	19	23 1
27	20	22 ar
28	20	24 1
29	22	34 1
30	25	26 ar
31	25	27 ar
32	26	28 ar
33	26	29 1
34	27	30 ar
35	27	36 1
36	28	31 ar
37	28	32 1
38	30	31 ar
39	30	33 1
40	31	37 1
41	34	52 1
42	35	65 1
43	36	78 1
44	37	91 1
45	38	39 1
46	38	40 1
47	38	41 1
48	41	42 ar
49	41	43 ar
50	42	44 ar
51	42	45 1
52	43	46 ar
53	43	47 1
54	44	48 ar
55	44	49 1
56	46	48 ar
57	46	50 1
58	48	51 1
59	52	53 1
60	52	54 1
61	52	55 1

```
  62    53    56 1
  63    53    57 1
  64    53    58 1
  65    56    59 1
  66    56    60 1
  67    56    61 1
  68    59    62 1
  69    59    63 1
  70    59    64 1
  71    65    66 1
  72    65    67 1
  73    65    68 1
  74    66    69 1
  75    66    70 1
  76    66    71 1
  77    69    72 1
  78    69    73 1
  79    69    74 1
  80    72    75 1
  81    72    76 1
  82    72    77 1
  83    78    79 1
  84    78    80 1
  85    78    81 1
  86    79    82 1
  87    79    83 1
  88    79    84 1
  89    82    85 1
  90    82    86 1
  91    82    87 1
  92    85    88 1
  93    85    89 1
  94    85    90 1
  95    91    92 1
  96    91    93 1
  97    91    94 1
  98    92    95 1
  99    92    96 1
 100    92    97 1
 101    95    98 1
 102    95    99 1
 103    95   100 1
 104    98   101 1
 105    98   102 1
 106    98   103 1
@<TRIPOS>SUBSTRUCTURE
     1 MOL         1 TEMP              0 ****  ****    0 ROOT
```

2m

```
@<TRIPOS>MOLECULE
```

```
MOL
  122  124     1     0     0
SMALL
resp


@<TRIPOS>ATOM
      1 C1          0.8370    -1.4230     0.2680 c3         1 MOL      -0.070669
      2 C2         -1.5200    -1.1650     0.0990 c3         1 MOL      -0.070669
      3 C3         -0.0760    -0.0660    -1.7380 c          1 MOL       0.470040
      4 H1         -2.4650    -1.6830     0.1840 h1         1 MOL       0.103622
      5 H2          0.8280    -0.7120     1.0920 h1         1 MOL       0.103622
      6 H3          1.6350    -2.1160     0.4810 h1         1 MOL       0.103622
      7 H4         -1.4850    -0.4560     0.9190 h1         1 MOL       0.103622
      8 N1         -0.4380    -2.1140     0.2870 n3         1 MOL      -0.480259
      9 O1          0.0620     0.6480    -2.6920 o          1 MOL      -0.545937
     10 C4         -1.4430    -0.4280    -1.2240 ce         1 MOL       0.007683
     11 C5          1.1080    -0.6640    -1.0240 ce         1 MOL       0.007683
     12 C6         -2.4760    -0.1250    -2.0040 cf         1 MOL      -0.139383
     13 H5         -2.2480     0.3410    -2.9470 ha         1 MOL       0.147991
     14 C7          2.3040    -0.4350    -1.5580 cf         1 MOL      -0.139383
     15 H6          2.3360     0.2040    -2.4220 ha         1 MOL       0.147991
     16 C8         -3.9140    -0.3980    -1.7610 ca         1 MOL       0.035736
     17 C9         -4.5810     0.0030    -0.6050 ca         1 MOL       0.119429
     18 C10        -4.6480    -1.0510    -2.7500 ca         1 MOL      -0.247899
     19 C11        -5.9370    -0.2670    -0.4370 ca         1 MOL       0.166862
     20 C12        -5.9890    -1.3290    -2.5870 ca         1 MOL      -0.185979
     21 H7         -4.1500    -1.3530    -3.6540 ha         1 MOL       0.170779
     22 C13        -6.6390    -0.9410    -1.4260 ca         1 MOL       0.116916
     23 H8         -6.5450    -1.8520    -3.3430 ha         1 MOL       0.173218
     24 C14         3.6190    -0.9440    -1.0890 ca         1 MOL       0.035736
     25 C15         3.8940    -2.3020    -0.9700 ca         1 MOL      -0.247899
     26 C16         4.6480    -0.0380    -0.8420 ca         1 MOL       0.119429
     27 C17         5.1440    -2.7470    -0.5820 ca         1 MOL      -0.185979
     28 H9          3.1280    -3.0170    -1.2060 ha         1 MOL       0.170779
     29 C18         5.9070    -0.4760    -0.4470 ca         1 MOL       0.166862
     30 C19         6.1530    -1.8370    -0.3170 ca         1 MOL       0.116916
     31 H10         5.3600    -3.7960    -0.5030 ha         1 MOL       0.173218
     32 O2         -7.9480    -1.2490    -1.2460 os         1 MOL      -0.356461
     33 O3         -3.9220     0.6530     0.3870 os         1 MOL      -0.324713
     34 O4          4.4330     1.2880    -1.0290 os         1 MOL      -0.324713
     35 O5          7.3910    -2.2770     0.0210 os         1 MOL      -0.356461
     36 C20        -0.5090    -3.2900    -0.5690 c3         1 MOL       0.165339
     37 H11        -1.5570    -3.5340    -0.7000 h1         1 MOL       0.017287
     38 C21        -8.8730    -0.2070    -1.5010 c3         1 MOL       0.074099
     39 C22       -10.2650    -0.7380    -1.2140 c3         1 MOL       0.005678
     40 H12        -8.7840     0.1030    -2.5390 h1         1 MOL       0.045809
     41 H13        -8.6440     0.6440    -0.8710 h1         1 MOL       0.045809
     42 C23       -11.3550     0.3050    -1.4740 c3         1 MOL       0.041101
     43 H14       -10.4360    -1.6170    -1.8290 hc         1 MOL       0.028135
     44 H15       -10.3030    -1.0660    -0.1790 hc         1 MOL       0.028135
```

45 C24	-12.7580	-0.2240	-1.1810 c3	1 MOL	-0.082126
46 H16	-11.1680	1.1860	-0.8630 hc	1 MOL	-0.001690
47 H17	-11.3040	0.6340	-2.5090 hc	1 MOL	-0.001690
48 H18	-13.5110	0.5340	-1.3730 hc	1 MOL	0.016896
49 H19	-12.9890	-1.0860	-1.8000 hc	1 MOL	0.016896
50 H20	-12.8530	-0.5290	-0.1430 hc	1 MOL	0.016896
51 C25	-4.0330	2.0660	0.3740 c3	1 MOL	0.020302
52 C26	-3.2690	2.6110	1.5660 c3	1 MOL	0.003620
53 H21	-5.0790	2.3440	0.4240 h1	1 MOL	0.052687
54 H22	-3.6200	2.4470	-0.5550 h1	1 MOL	0.052687
55 C27	-3.3320	4.1370	1.6520 c3	1 MOL	0.043631
56 H23	-3.6780	2.1690	2.4710 hc	1 MOL	0.031884
57 H24	-2.2340	2.2860	1.4960 hc	1 MOL	0.031884
58 C28	-2.5620	4.6910	2.8510 c3	1 MOL	-0.090112
59 H25	-2.9320	4.5690	0.7380 hc	1 MOL	0.003368
60 H26	-4.3700	4.4550	1.7140 hc	1 MOL	0.003368
61 H27	-2.6230	5.7740	2.8890 hc	1 MOL	0.018257
62 H28	-2.9590	4.3040	3.7840 hc	1 MOL	0.018257
63 H29	-1.5120	4.4200	2.8010 hc	1 MOL	0.018257
64 C29	4.1340	2.0470	0.1300 c3	1 MOL	0.020302
65 C30	3.9070	3.4840	-0.3000 c3	1 MOL	0.003620
66 H30	4.9580	1.9740	0.8300 h1	1 MOL	0.052687
67 H31	3.2450	1.6420	0.6040 h1	1 MOL	0.052687
68 C31	3.5620	4.3990	0.8770 c3	1 MOL	0.043631
69 H32	4.8040	3.8420	-0.7990 hc	1 MOL	0.031884
70 H33	3.1080	3.5040	-1.0350 hc	1 MOL	0.031884
71 C32	3.3370	5.8490	0.4490 c3	1 MOL	-0.090112
72 H34	2.6680	4.0290	1.3750 hc	1 MOL	0.003368
73 H35	4.3610	4.3620	1.6150 hc	1 MOL	0.003368
74 H36	3.0920	6.4760	1.3000 hc	1 MOL	0.018257
75 H37	4.2250	6.2600	-0.0210 hc	1 MOL	0.018257
76 H38	2.5210	5.9240	-0.2640 hc	1 MOL	0.018257
77 C33	7.7150	-2.2240	1.3990 c3	1 MOL	0.074099
78 C34	9.1400	-2.7210	1.5640 c3	1 MOL	0.005678
79 H39	7.0210	-2.8510	1.9530 h1	1 MOL	0.045809
80 H40	7.6150	-1.2070	1.7570 h1	1 MOL	0.045809
81 C35	9.5930	-2.7240	3.0250 c3	1 MOL	0.041101
82 H41	9.2090	-3.7230	1.1490 hc	1 MOL	0.028135
83 H42	9.7960	-2.0900	0.9710 hc	1 MOL	0.028135
84 C36	11.0290	-3.2190	3.1940 c3	1 MOL	-0.082126
85 H43	9.5080	-1.7200	3.4340 hc	1 MOL	-0.001690
86 H44	8.9260	-3.3520	3.6120 hc	1 MOL	-0.001690
87 H45	11.3260	-3.2120	4.2390 hc	1 MOL	0.016896
88 H46	11.1400	-4.2340	2.8250 hc	1 MOL	0.016896
89 H47	11.7270	-2.5910	2.6480 hc	1 MOL	0.016896
90 H48	-0.1110	-3.1040	-1.5690 h1	1 MOL	0.017287
91 C37	0.1940	-4.4950	0.0470 c3	1 MOL	-0.052341
92 H49	1.2590	-4.3310	0.1730 hc	1 MOL	0.014375
93 H50	-0.2260	-4.7160	1.0220 hc	1 MOL	0.014375
94 H51	0.0710	-5.3680	-0.5890 hc	1 MOL	0.014375
95 O6	6.8840	0.4250	-0.1730 os	1 MOL	-0.308121

```
  96 O7         -6.5770    0.1610    0.6800 os         1 MOL      -0.308121
  97 C38         7.7000    0.7900   -1.2740 c3         1 MOL       0.035368
  98 C39         8.7250    1.7960   -0.7860 c3         1 MOL      -0.022166
  99 H52         7.0780    1.2170   -2.0530 h1         1 MOL       0.065995
 100 H53         8.1840   -0.0970   -1.6680 h1         1 MOL       0.065995
 101 C40         9.6650    2.2610   -1.8990 c3         1 MOL       0.047503
 102 H54         8.2010    2.6490   -0.3620 hc         1 MOL       0.034854
 103 H55         9.2980    1.3470    0.0210 hc         1 MOL       0.034854
 104 C41        10.6990    3.2760   -1.4130 c3         1 MOL      -0.077518
 105 H56        10.1780    1.4010   -2.3250 hc         1 MOL      -0.002030
 106 H57         9.0830    2.7000   -2.7070 hc         1 MOL      -0.002030
 107 H58        11.3520    3.5880   -2.2210 hc         1 MOL       0.015744
 108 H59        10.2190    4.1640   -1.0130 hc         1 MOL       0.015744
 109 H60        11.3210    2.8550   -0.6280 hc         1 MOL       0.015744
 110 C42        -6.5720   -0.7470    1.7690 c3         1 MOL       0.035368
 111 C43        -7.3260   -0.1050    2.9190 c3         1 MOL      -0.022166
 112 H61        -5.5470   -0.9610    2.0510 h1         1 MOL       0.065995
 113 H62        -7.0460   -1.6730    1.4630 h1         1 MOL       0.065995
 114 C44        -7.3830   -1.0050    4.1550 c3         1 MOL       0.047503
 115 H63        -6.8460    0.8380    3.1670 hc         1 MOL       0.034854
 116 H64        -8.3330    0.1320    2.5850 hc         1 MOL       0.034854
 117 C45        -8.1420   -0.3630    5.3170 c3         1 MOL      -0.077518
 118 H65        -7.8550   -1.9500    3.8960 hc         1 MOL      -0.002030
 119 H66        -6.3720   -1.2460    4.4760 hc         1 MOL      -0.002030
 120 H67        -8.1670   -1.0210    6.1800 hc         1 MOL       0.015744
 121 H68        -7.6740    0.5680    5.6230 hc         1 MOL       0.015744
 122 H69        -9.1680   -0.1410    5.0390 hc         1 MOL       0.015744
@<TRIPOS>BOND
     1     1     5 1
     2     1     6 1
     3     1     8 1
     4     1    11 1
     5     2     4 1
     6     2     7 1
     7     2     8 1
     8     2    10 1
     9     3     9 2
    10     3    10 1
    11     3    11 1
    12     8    36 1
    13    10    12 2
    14    11    14 2
    15    12    13 1
    16    12    16 1
    17    14    15 1
    18    14    24 1
    19    16    17 ar
    20    16    18 ar
    21    17    19 ar
    22    17    33 1
    23    18    20 ar
```

24	18	21 1
25	19	22 ar
26	19	96 1
27	20	22 ar
28	20	23 1
29	22	32 1
30	24	25 ar
31	24	26 ar
32	25	27 ar
33	25	28 1
34	26	29 ar
35	26	34 1
36	27	30 ar
37	27	31 1
38	29	30 ar
39	29	95 1
40	30	35 1
41	32	38 1
42	33	51 1
43	34	64 1
44	35	77 1
45	36	37 1
46	36	90 1
47	36	91 1
48	38	39 1
49	38	40 1
50	38	41 1
51	39	42 1
52	39	43 1
53	39	44 1
54	42	45 1
55	42	46 1
56	42	47 1
57	45	48 1
58	45	49 1
59	45	50 1
60	51	52 1
61	51	53 1
62	51	54 1
63	52	55 1
64	52	56 1
65	52	57 1
66	55	58 1
67	55	59 1
68	55	60 1
69	58	61 1
70	58	62 1
71	58	63 1
72	64	65 1
73	64	66 1
74	64	67 1

```
  75    65    68 1
  76    65    69 1
  77    65    70 1
  78    68    71 1
  79    68    72 1
  80    68    73 1
  81    71    74 1
  82    71    75 1
  83    71    76 1
  84    77    78 1
  85    77    79 1
  86    77    80 1
  87    78    81 1
  88    78    82 1
  89    78    83 1
  90    81    84 1
  91    81    85 1
  92    81    86 1
  93    84    87 1
  94    84    88 1
  95    84    89 1
  96    91    92 1
  97    91    93 1
  98    91    94 1
  99    95    97 1
 100    96   110 1
 101    97    98 1
 102    97    99 1
 103    97   100 1
 104    98   101 1
 105    98   102 1
 106    98   103 1
 107   101   104 1
 108   101   105 1
 109   101   106 1
 110   104   107 1
 111   104   108 1
 112   104   109 1
 113   110   111 1
 114   110   112 1
 115   110   113 1
 116   111   114 1
 117   111   115 1
 118   111   116 1
 119   114   117 1
 120   114   118 1
 121   114   119 1
 122   117   120 1
 123   117   121 1
 124   117   122 1
@<TRIPOS>SUBSTRUCTURE
```

1 MOL 1 TEMP 0 **** **** 0 ROOT

3iso-h

@<TRIPOS>MOLECULE
MOL
103 106 1 0 0
SMALL
resp

@<TRIPOS>ATOM

1	C1	-0.9520	-0.2560	-1.0620	c3	1	MOL	-0.110762
2	C2	1.4200	-0.0880	-0.7540	c3	1	MOL	-0.110762
3	C3	0.0740	-1.3140	1.0780	c	1	MOL	0.461656
4	H1	2.3670	-0.2160	-1.2600	h1	1	MOL	0.113496
5	H2	-1.0900	0.8130	-0.9060	h1	1	MOL	0.113496
6	H3	-1.7080	-0.5550	-1.7720	h1	1	MOL	0.113496
7	H4	1.3220	0.9760	-0.5400	h1	1	MOL	0.113496
8	N1	0.3500	-0.4410	-1.6580	n3	1	MOL	-0.387660
9	O1	-0.0190	-1.9100	2.1150	o	1	MOL	-0.529550
10	C4	1.4100	-0.8740	0.5460	ce	1	MOL	-0.024678
11	C5	-1.1510	-0.9550	0.2770	ce	1	MOL	-0.024678
12	C6	2.4800	-1.2430	1.2460	cf	1	MOL	-0.106587
13	H5	2.2920	-1.8550	2.1090	ha	1	MOL	0.146460
14	C7	-2.3250	-1.1840	0.8540	cf	1	MOL	-0.106587
15	H6	-2.3000	-1.6050	1.8440	ha	1	MOL	0.146460
16	C8	3.9060	-0.9670	0.9700	ca	1	MOL	-0.010242
17	C9	4.3580	0.2770	0.5170	ca	1	MOL	-0.185060
18	C10	4.8340	-1.9700	1.2030	ca	1	MOL	-0.165876
19	C11	5.7020	0.5050	0.2790	ca	1	MOL	0.142228
20	C12	6.1810	-1.7470	0.9550	ca	1	MOL	-0.250742
21	H7	4.5080	-2.9280	1.5660	ha	1	MOL	0.156751
22	C13	6.6240	-0.5260	0.4960	ca	1	MOL	0.242953
23	H8	6.9060	-2.5250	1.1100	ha	1	MOL	0.180430
24	C14	-3.6790	-0.8810	0.3200	ca	1	MOL	-0.010242
25	C15	-4.1450	-1.3840	-0.8880	ca	1	MOL	-0.165876
26	C16	-4.5440	-0.1040	1.0890	ca	1	MOL	-0.185060
27	C17	-5.4270	-1.0920	-1.3270	ca	1	MOL	-0.250742
28	H9	-3.5190	-2.0220	-1.4820	ha	1	MOL	0.156751
29	C18	-5.8210	0.1940	0.6540	ca	1	MOL	0.142228
30	C19	-6.2680	-0.2980	-0.5700	ca	1	MOL	0.242953
31	H10	-5.7930	-1.4870	-2.2570	ha	1	MOL	0.180430
32	O2	7.9400	-0.3430	0.2260	os	1	MOL	-0.381897
33	O3	-7.5300	-0.0410	-1.0000	os	1	MOL	-0.381897
34	C20	0.5630	-1.5640	-2.5580	c3	1	MOL	-0.064718
35	H11	1.4340	-1.3220	-3.1570	h1	1	MOL	0.099004
36	H12	-0.2810	-1.5800	-3.2400	h1	1	MOL	0.099004
37	C21	0.7570	-2.9610	-1.9810	ca	1	MOL	0.016980
38	C22	-0.3300	-3.7700	-1.6660	ca	1	MOL	-0.120137
39	C23	2.0350	-3.4750	-1.7920	ca	1	MOL	-0.120137

40	C24	-0.1470	-5.0440	-1.1540	ca	1 MOL	-0.164547
41	H13	-1.3290	-3.4060	-1.8240	ha	1 MOL	0.135176
42	C25	2.2250	-4.7490	-1.2830	ca	1 MOL	-0.164547
43	H14	2.8920	-2.8740	-2.0420	ha	1 MOL	0.135176
44	C26	1.1330	-5.5370	-0.9590	ca	1 MOL	-0.087634
45	H15	-1.0020	-5.6510	-0.9130	ha	1 MOL	0.134316
46	H16	3.2230	-5.1250	-1.1440	ha	1 MOL	0.134316
47	H17	1.2770	-6.5270	-0.5640	ha	1 MOL	0.122838
48	C27	8.6940	0.3400	1.2080	c3	1 MOL	0.071828
49	C28	10.1130	0.4830	0.6910	c3	1 MOL	0.013243
50	H18	8.6750	-0.2300	2.1340	h1	1 MOL	0.041554
51	H19	8.2540	1.3110	1.4000	h1	1 MOL	0.041554
52	C29	11.0310	1.1980	1.6840	c3	1 MOL	0.040493
53	H20	10.5040	-0.5060	0.4680	hc	1 MOL	0.024505
54	H21	10.0830	1.0270	-0.2490	hc	1 MOL	0.024505
55	C30	12.4600	1.3470	1.1630	c3	1 MOL	-0.076437
56	H22	10.6270	2.1830	1.9090	hc	1 MOL	-0.001896
57	H23	11.0470	0.6520	2.6250	hc	1 MOL	-0.001896
58	H24	13.0900	1.8570	1.8860	hc	1 MOL	0.015152
59	H25	12.9060	0.3780	0.9610	hc	1 MOL	0.015152
60	H26	12.4830	1.9200	0.2410	hc	1 MOL	0.015152
61	C31	-7.7320	1.2260	-1.6010	c3	1 MOL	0.071828
62	C32	-9.1970	1.3380	-1.9800	c3	1 MOL	0.013243
63	H27	-7.0980	1.3080	-2.4800	h1	1 MOL	0.041554
64	H28	-7.4490	2.0070	-0.9060	h1	1 MOL	0.041554
65	C33	-9.5250	2.6680	-2.6600	c3	1 MOL	0.040493
66	H29	-9.4510	0.5110	-2.6370	hc	1 MOL	0.024505
67	H30	-9.7970	1.2210	-1.0820	hc	1 MOL	0.024505
68	C34	-11.0010	2.7880	-3.0390	c3	1 MOL	-0.076437
69	H31	-9.2560	3.4900	-2.0010	hc	1 MOL	-0.001896
70	H32	-8.9150	2.7810	-3.5540	hc	1 MOL	-0.001896
71	H33	-11.2070	3.7390	-3.5200	hc	1 MOL	0.015152
72	H34	-11.2940	1.9990	-3.7260	hc	1 MOL	0.015152
73	H35	-11.6370	2.7150	-2.1620	hc	1 MOL	0.015152
74	H36	3.6510	1.0690	0.3810	ha	1 MOL	0.146145
75	H37	-4.2260	0.2910	2.0370	ha	1 MOL	0.146145
76	O4	-6.6230	0.9870	1.4110	os	1 MOL	-0.308182
77	O5	6.2130	1.6760	-0.1510	os	1 MOL	-0.308182
78	C35	5.3620	2.7450	-0.4790	c3	1 MOL	0.095391
79	C36	6.2260	3.8830	-0.9930	c3	1 MOL	0.012836
80	H38	4.8020	3.0580	0.3980	h1	1 MOL	0.037198
81	H39	4.6510	2.4320	-1.2380	h1	1 MOL	0.037198
82	C37	5.4030	5.1100	-1.3890	c3	1 MOL	0.046874
83	H40	6.9440	4.1490	-0.2210	hc	1 MOL	0.021293
84	H41	6.7980	3.5250	-1.8440	hc	1 MOL	0.021293
85	C38	6.2700	6.2570	-1.9060	c3	1 MOL	-0.062200
86	H42	4.6810	4.8320	-2.1540	hc	1 MOL	-0.008547
87	H43	4.8250	5.4530	-0.5330	hc	1 MOL	-0.008547
88	H44	5.6630	7.1140	-2.1800	hc	1 MOL	0.012199
89	H45	6.9800	6.5820	-1.1500	hc	1 MOL	0.012199
90	H46	6.8350	5.9560	-2.7830	hc	1 MOL	0.012199

```
  91 C39       -7.5860    0.3140    2.2040 c3       1 MOL      0.095391
  92 C40       -8.3950    1.3600    2.9480 c3       1 MOL      0.012836
  93 H47       -7.0750   -0.3460    2.9000 h1       1 MOL      0.037198
  94 H48       -8.2210   -0.2910    1.5680 h1       1 MOL      0.037198
  95 C41       -9.4580    0.7400    3.8560 c3       1 MOL      0.046874
  96 H49       -7.7160    1.9730    3.5330 hc       1 MOL      0.021293
  97 H50       -8.8640    2.0170    2.2210 hc       1 MOL      0.021293
  98 C42      -10.2790    1.7900    4.6040 c3       1 MOL     -0.062200
  99 H51      -10.1250    0.1170    3.2640 hc       1 MOL     -0.008547
 100 H52       -8.9810    0.0780    4.5760 hc       1 MOL     -0.008547
 101 H53      -11.0250    1.3240    5.2410 hc       1 MOL      0.012199
 102 H54       -9.6450    2.4070    5.2330 hc       1 MOL      0.012199
 103 H55      -10.7980    2.4470    3.9120 hc       1 MOL      0.012199
@<TRIPOS>BOND
     1     1     5 1
     2     1     6 1
     3     1     8 1
     4     1    11 1
     5     2     4 1
     6     2     7 1
     7     2     8 1
     8     2    10 1
     9     3     9 2
    10     3    10 1
    11     3    11 1
    12     8    34 1
    13    10    12 2
    14    11    14 2
    15    12    13 1
    16    12    16 1
    17    14    15 1
    18    14    24 1
    19    16    17 ar
    20    16    18 ar
    21    17    19 ar
    22    17    74 1
    23    18    20 ar
    24    18    21 1
    25    19    22 ar
    26    19    77 1
    27    20    22 ar
    28    20    23 1
    29    22    32 1
    30    24    25 ar
    31    24    26 ar
    32    25    27 ar
    33    25    28 1
    34    26    29 ar
    35    26    75 1
    36    27    30 ar
    37    27    31 1
```

38	29	30 ar
39	29	76 1
40	30	33 1
41	32	48 1
42	33	61 1
43	34	35 1
44	34	36 1
45	34	37 1
46	37	38 ar
47	37	39 ar
48	38	40 ar
49	38	41 1
50	39	42 ar
51	39	43 1
52	40	44 ar
53	40	45 1
54	42	44 ar
55	42	46 1
56	44	47 1
57	48	49 1
58	48	50 1
59	48	51 1
60	49	52 1
61	49	53 1
62	49	54 1
63	52	55 1
64	52	56 1
65	52	57 1
66	55	58 1
67	55	59 1
68	55	60 1
69	61	62 1
70	61	63 1
71	61	64 1
72	62	65 1
73	62	66 1
74	62	67 1
75	65	68 1
76	65	69 1
77	65	70 1
78	68	71 1
79	68	72 1
80	68	73 1
81	76	91 1
82	77	78 1
83	78	79 1
84	78	80 1
85	78	81 1
86	79	82 1
87	79	83 1
88	79	84 1

```
  89    82    85 1
  90    82    86 1
  91    82    87 1
  92    85    88 1
  93    85    89 1
  94    85    90 1
  95    91    92 1
  96    91    93 1
  97    91    94 1
  98    92    95 1
  99    92    96 1
 100    92    97 1
 101    95    98 1
 102    95    99 1
 103    95   100 1
 104    98   101 1
 105    98   102 1
 106    98   103 1
@<TRIPOS>SUBSTRUCTURE
     1 MOL         1 TEMP              0 ****  ****    0 ROOT
```
